# A redox-regulated RCC1-like protein controls catalase activity in Arabidopsis

**DOI:** 10.1038/s41467-026-76792-x

**Published:** 2026-08-15

**Authors:** Zhoubo Hu, Sara Christina Stolze, Amna Mhamdi, Frank Van Breusegem, Hirofumi Nakagami, Roman Ulm

**Affiliations:** 1 Department of Plant Sciences, Section of Biology, Faculty of Sciences, University of Geneva, Geneva, Switzerland; 2 Max-Planck Institute for Plant Breeding Research, Cologne, Germany; 3 Department of Plant Biotechnology and Bioinformatics, Ghent University, Ghent, Belgium; 4 Center for Plant Systems Biology, VIB, Ghent, Belgium; 5 Institute of Genetics and Genomics of Geneva (iGE3), University of Geneva, Geneva, Switzerland

**Keywords:** Plant signalling, Abiotic, Plant physiology, Light responses

## Abstract

Reactive oxygen species (ROS) regulate plant growth and stress responses. Catalases play a central role in detoxifying hydrogen peroxide, predominantly within peroxisomes, yet key aspects of catalase regulation remain incompletely understood. Using affinity purification of the UV-B photoreceptor UVR8 coupled with mass spectrometry, we identified CATALASE-INTERACTING RCC1-LIKE 1 (CAIR1), which interacts with all three Arabidopsis catalases and their chaperone NO CATALASE ACTIVITY 1. Loss of CAIR1 reduces catalase activity and causes oxidative stress sensitivity, impaired root growth, and alkaline sensitivity, resembling *cat2* and *nca1* mutants. CAIR1 promotes peroxisomal import and proper localization of CAT2, preventing CAT2 aggregation and maintaining its activity. CAIR1 undergoes reversible redox-dependent oligomerization that enhances catalase binding, whereas mutation of Cys-356 and Cys-545 compromises this interaction and fails to rescue the oxidative stress sensitivity of *cair1* mutants. UV-B weakens CAIR1-catalase interactions and suppresses catalase activity, linking light signalling with redox homeostasis. These findings identify CAIR1 as a redox-responsive regulator of catalase localization and activity.

## Abstract (web summary)

Researchers identify CAIR1 as a redox-responsive regulator that promotes catalase localization and activity, helping plants maintain hydrogen peroxide balance and cellular redox homeostasis.

## Introduction

The build-up of reactive oxygen species (ROS) within various cellular compartments in response to stress can lead to oxidative stress, ultimately compromising both cellular and organellar integrity. Key ROS include singlet oxygen (^1^O_2_), superoxide anion (O_2_^•−^), hydrogen peroxide (H_2_O_2_), and hydroxyl radicals (•OH); species that can interconvert within the cellular environment. Despite their damaging potential, ROS also serve as important signaling molecules that initiate pathways involved in plant growth, development, and adaptation to stress^1–4^. As such, maintaining a delicate balance in ROS levels is essential. For ROS homeostasis, plants rely on a complex network of non-enzymatic antioxidants and processing enzymes such as glutathione, ascorbate, ascorbate peroxidases, superoxide dismutases, and catalases (CAT), all of which are crucial for sustaining ROS homeostasis^1,5^.

Peroxisomes are single-membrane organelles involved in a variety of metabolic processes. In plants, peroxisomes play essential roles in key metabolic pathways such as photorespiration and lipid mobilization through fatty acid β-oxidation^6–11^. Photorespiration is a metabolic process in plants whereby the enzyme ribulose-1,5-bisphosphate carboxylase/oxygenase (Rubisco) fixes oxygen instead of carbon dioxide, resulting in reduced photosynthetic efficiency and the production of harmful ROS in peroxisomes^12^. Fatty acid β-oxidation provides energy for post-germinative growth and development by breaking down fats stored as triacylglycerol in oil bodies of oilseed plants such as *Arabidopsis thaliana* (herein Arabidopsis)^13^. Both fatty acid β-oxidation and photorespiration generate H_2_O_2_ in peroxisomes, which is predominantly scavenged by catalases^14,15^. Catalases are tetrameric heme-containing antioxidant enzymes that catalyze the breakdown of H_2_O_2_, and thereby play a critical role in maintaining H_2_O_2_ homeostasis^16–19^. Import of proteins such as catalases into peroxisomes for these essential functions primarily depends on peroxisome targeting signals (PTS)^20^. Proteins with PTS require cytosolic PEROXIN-5 (PEX5) and PEX7 proteins for import^21–23^, whereas PTS-independent access to peroxisomes is regulated by “piggy-back” co-import with a peroxisomal-targeted, PTS-containing protein^24,25^. Outside of peroxisomes, catalases are localized to and perform roles in the nucleus and cytosol, as recently described^15,26–28^. Indeed, mammalian catalases play key roles in defending against acute oxidative stress in the cytosol^29^ and cytosolic catalase levels are regulated directly at the level of their peroxisomal import^30^.

As in most flowering plants, the Arabidopsis genome encodes three catalase isoforms, namely CAT1, −2, and −3^5,31^. Among these, CAT2 is the major isoform in leaves, accounting for approximately 90% of total catalase activity^5,6,32,33^. Arabidopsis catalases are imported into peroxisomes by a non-canonical import pathway^15,34^. Although the C-terminal 11 amino acids were shown to be required for plant catalase localization to peroxisomes in transient expression systems^35^, they are not essential for peroxisomal targeting in stably transformed Arabidopsis plants^19,28^. Furthermore, different interactors of catalases were identified and linked to the regulation of catalase activity, including calcium-regulated calmodulin (CaM), the zinc finger protein LESION SIMULATING DISEASE 1 (LSD1), NUCLEOREDOXIN 1 (NRX1), and NO CATALASE ACTIVITY 1 (NCA1) as positive regulators^15,27,36–39^. Notably, NCA1, a protein with an N-terminal RING-finger domain and a C-terminal tetratricopeptide repeat-like helical domain, interacts with catalases in the cytosol, where it chaperones catalases to maintain stability and activity, hence playing an important role in multiple stress responses, including in response to enhanced photorespiration^40–42^.

Mammalian and fungal Regulator of Chromatin Condensation 1 (RCC1) proteins are conserved eukaryotic chromatin-associated proteins consisting of seven RCC1-repeats that function as guanine nucleotide exchange factors (GEFs) for the small GTPase Ran. However, several proteins with one or more RCC1-like structural domains (RLDs) of 51–68 amino acid RCC1-like repeats exhibit diverse non-GEF functions^43^. The Arabidopsis genome encodes 24 RLD-containing proteins^44^, of which only a few are functionally characterized and shown to be involved in a diverse range of functions, including efficient splicing of *nad2* mRNA and accumulation of mitochondrial respiratory chain complex I^45,46^, plasticity of rosette size in response to nitrogen availability^47^, abscisic acid signaling^48^, cold acclimation and freezing tolerance^49^, gravity signaling and regulation of polar auxin transport^50^, and ultraviolet-B (UV-B) sensing and signaling^51,52^. The UV-B photoreceptor UV RESISTANCE LOCUS 8 (UVR8) is a member of the RCC1-like protein family in plants^51,53^ that, however, has no Ran-GEF activity^54^ and does not directly bind to chromatin^55^. The cytosolic homodimeric form of UVR8 monomerizes upon UV-B photon reception, after which it accumulates in the nucleus to regulate downstream gene expression, thus mediating UV-B-dependent photomorphogenic responses, including acclimation and UV-B stress protection^52^.

Here, we identify a previously uncharacterized RCC1-like protein, CATALASE-INTERACTING RCC1-LIKE 1 (CAIR1), as a regulator of catalase activity in Arabidopsis. CAIR1 associates with all three catalases and their chaperone NCA1 in the cytosol and is required for normal catalase function in vivo. Our results indicate that CAIR1 function is modulated by the cellular redox state, uncovering a regulatory link between catalase homeostasis and dynamic redox signaling.

## Results

### UVR8 co-purifies and interacts with AT5G11580, an RCC1-like family protein of unknown function

We generated an N-terminally GFP-tagged UVR8 genomic clone by fast-track BAC recombineering^56^ that complemented the *uvr8-6* null mutant allele (Supplementary Fig. 1A–C). We used this line for AP–MS using anti-GFP nanobodies/VHH coupled to agarose beads (GFP-trap®) for complex purification, which identified previously known UVR8 core photocycle proteins REPRESSOR OF UV-B PHOTOMORPHOGENESIS 1 (RUP1), RUP2, CONSTITUTIVELY PHOTOMORPHOGENIC 1 (COP1), as well as the COP1-associated SUPPRESSOR OF PHYA-105 (SPA) proteins SPA1-to-SPA4, specifically when plants were grown in the presence of supplementary UV-B (Supplementary Fig. 1D and Supplementary Data 1)^57–59^. In addition, a RCC1-like protein (AT5G11580; herein CAIR1, see below) of previously unknown function co-purified, both in the absence and presence of UV-B (Supplementary Fig. 1D). Consistently, CAIR1 interacted with UVR8 in co-immunoprecipitation (co-IP) assays when co-expressed in human embryonic kidney (HEK293) cells, as well as in luciferase complementation imaging (LCI) assays in *Nicotiana benthamiana* epidermal leaf cells (Supplementary Fig. 1E, F).

CAIR1 is a 553-amino acid protein that contains six predicted tandem RCC1 repeats but no additional predicted functional domain (Supplementary Fig. 2A, B). Phylogenetic analysis of CAIR1 protein sequences reveals that orthologs are present as one or two copies in angiosperms, gymnosperms, and ferns (Supplementary Fig. 2C). In contrast, no putative orthologs were detected in the chlorophytes, liverworts, mosses, and lycophytes sampled in this study (Supplementary Fig. 2C); however, broader taxonomic sampling and additional genomic resources will be required to fully assess its distribution across early-diverging plant lineages. To investigate the potential role of Arabidopsis CAIR1 in UVR8 signaling, we isolated the *cair1-1* mutant containing a T-DNA insertion in the second intron and generated the additional *cair1-2* and *cair1-3* null alleles by CRISPR/Cas9 (Supplementary Fig. 3). Loss and overexpression of CAIR1 did not obviously affect well-known UVR8–mediated UV-B responses such as hypocotyl growth inhibition, anthocyanin accumulation, accumulation of UV-absorbing metabolites, activation of UV-B–responsive marker genes (*RUP2*, *HY5*, and *CHS*), and UV-B acclimation (Supplementary Fig. 4). We thus identify CAIR1 as a previously undescribed interactor of the UVR8 photoreceptor, with our findings indicating that its functional relevance may extend beyond the canonical UVR8 signaling pathway underlying UV-B–induced photomorphogenesis.

### CAIR1 (AT5G11580) interacts with catalases and their chaperone NCA1

**Fig. 1:**
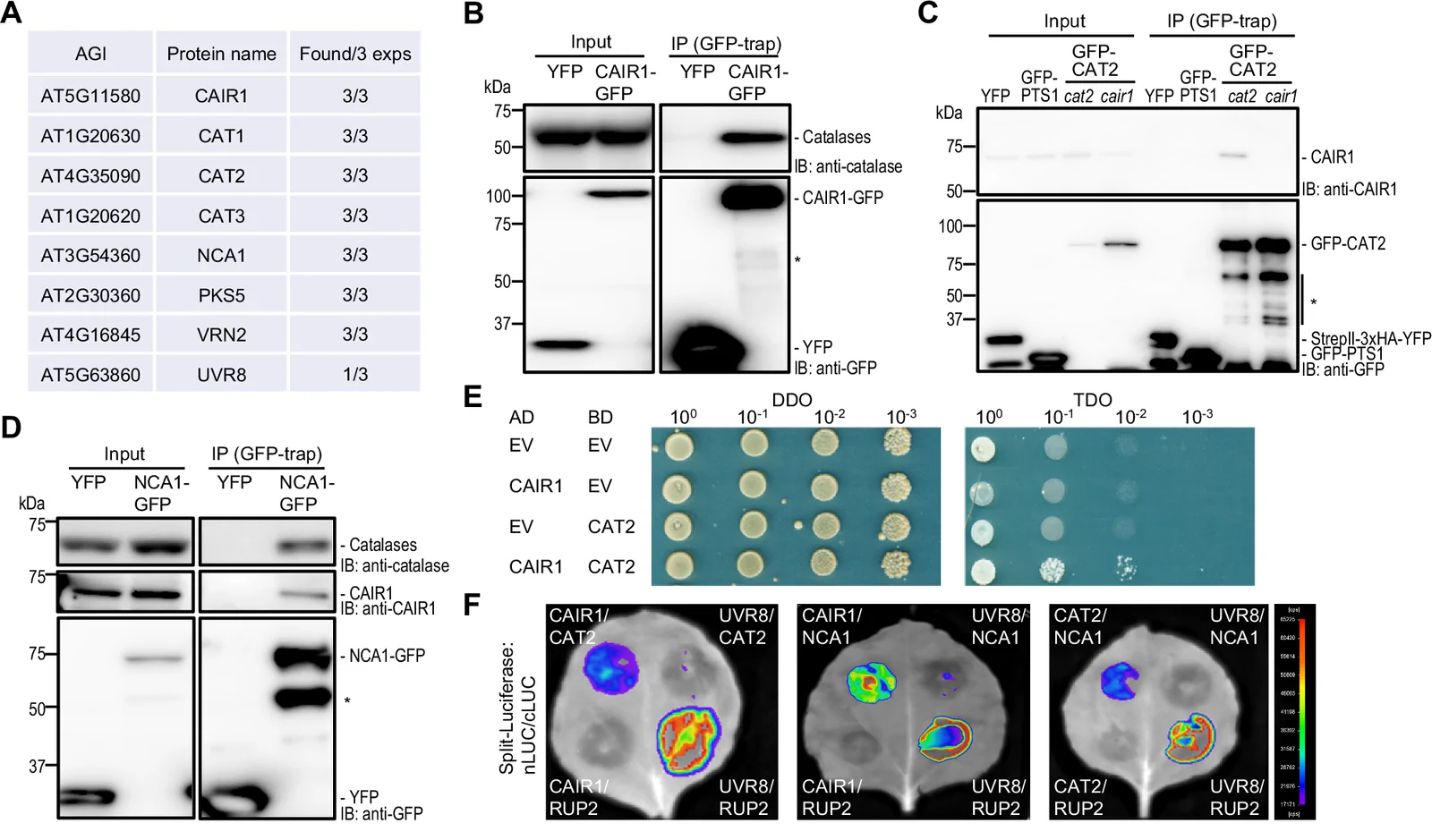
CAIR1 interacts with catalases and NCA1. **A** Summary of proteins identified in three independent AP–MS experiments of CAIR1 complexes (Supplementary Data 1). Depicted is a concise list of candidate proteins significantly enriched in all three CAIR1 AP–MS experiments compared to the negative controls (wild type and Pro_35S_:StrepII-3xHA-YFP). Note that the 3 experiments represent AP–MS with *cair1-1*/Pro_35S_:YFP-CAIR1, *cair1-1*/Pro_35S_:CAIR1-YFP, and genomic complementation line *cair1-1*/Pro_CAIR1_:CAIR1-GFP; UVR8 co-purified with the latter. AGI = Arabidopsis Gene Identifier. **B** Co-IP assay of endogenous catalases using GFP-trap purification from extracts of 15-d-old Pro_35S_:StrepII-3xHA-YFP (YFP) and *cair1-1*/Pro_CAIR1_:CAIR1-GFP (CAIR1-GFP) transgenic seedlings. **C** Co-IP assay of endogenous CAIR1 using GFP-trap purification from extracts of 15-d-old Pro_35S_:StrepII-3xHA-YFP (YFP), Pro_35S_:GFP-PTS1 (GFP-PTS1), *cat2-2*/Pro_CAT2_:GFP-CAT2 (GFP-CAT2 *cat2*), and *cair1-1*/Pro_CAT2_:GFP-CAT2 (GFP-CAT2 *cair1*) transgenic seedlings. **D** Co-IP assay of endogenous CAIR1 and catalases using GFP-trap purification from extracts of 15-d-old Pro_35S_:StrepII-3xHA-YFP (YFP) and *nca1-1*/Pro_NCA1_:NCA1-GFP (NCA1-GFP) transgenic seedlings. **B**–**D** YFP and/or GFP-PTS1 transgenic lines were used as negative controls. IB, immunoblotting; IP, immunoprecipitation; asterisks (*) indicate likely degradation products that evolved during immunoprecipitation. **E** Interaction of CAIR1 with CAT2 in a yeast two-hybrid growth assay. Tenfold serial dilutions of transformed yeast spotted on SD/−Trp/−Leu (DDO; nonselective for interaction) and SD/−Trp/−Leu/−His (TDO; selective) plates. AD, activation domain; BD, DNA binding domain; EV, empty vector. **F** LCI assay using indicated combinations of 35S promoter–driven CAIR1-nLUC, cLUC-CAT2, cLUC-NCA1, UVR8-nLUC, and cLUC-RUP2 transient expression in *N. benthamiana* leaves. Results shown are representative of 3 independent repetitions.

To gain insight into the molecular function of CAIR1, we generated Pro_35S_:YFP-CAIR1 and Pro_35S_:CAIR1-YFP lines in the respective *cair1-1* null mutant background, as well as a genomic complementation line containing a C-terminal GFP tag. AP–MS was performed using GFP-trap agarose beads on proteins extracted from 15-d-old transgenic plants. As controls, protein extracts from wild-type and Pro_35S_:StrepII-3xHA-YFP (herein “YFP”) seedlings were processed in parallel to account for potential common contaminant (non-specifically binding) proteins. CAIR1-GFP expressed from the genomic complementation construct indeed reciprocally co-purified UVR8, but in all three cases (Pro_35S_:YFP-CAIR1, Pro_35S_:CAIR1-YFP, as well as genomic Pro_CAIR1_:CAIR1-GFP), tagged CAIR1 powerfully co-purified the three catalases CAT1, CAT2, and CAT3, as well as NCA1 (Fig. 1A and Supplementary Data 1); hence the name CATALASE INTERACTING RCC1-LIKE 1 (CAIR1) assigned for the previously undescribed AT5G11580. In agreement with the AP–MS data, CAIR1-GFP efficiently co-immunoprecipitated endogenous catalases (Fig. 1B), which were detected with an anti-catalase antibody that cross-reacts with all three catalases^32^. Moreover, immunoprecipitation of GFP-CAT2 and NCA1-GFP from functional genomic complementation lines *nca1-1/*Pro_NCA1_:NCA1-GFP and *cat2-2/*Pro_CAT2_:GFP-CAT2 (Supplementary Fig. 5A–D), respectively, both co-immunoprecipitated endogenous CAIR1 (Fig. 1C, D). Additionally, AP–MS of NCA1-GFP and GFP-CAT2 further corroborate that CAIR1 forms a complex with catalases and NCA1 (Supplementary Data 1; and see below). In contrast, CAIR1 was not co-immunoprecipitated with YFP alone and a peroxisomally localized GFP (GFP-PTS1), both providing negative controls (Fig. 1C, D and Supplementary Data 1). We further tested whether CAIR1 may affect the NCA1-catalase interaction using a *cair1-1*/Pro_NCA1_:NCA1-GFP line. However, the absence of CAIR1 did not substantially affect the co-IP of catalases with NCA1-GFP (Supplementary Fig. 6).

To complement the AP–MS and co-IP findings, we performed yeast two-hybrid (Y2H) assays to test for potential direct protein–protein interactions. Y2H assays confirmed that CAIR1 interacts with CAT1, CAT2, and CAT3, but notably did not show CAIR1 interaction with NCA1 (Fig. 1E and Supplementary Fig. 7A). However, interaction between NCA1 and CAT2 was detectable (Supplementary Fig. 7A). In an additional orthogonal assay, heterologously expressed GFP-CAIR1 (but not GFP alone) in HEK293 cells co-immunoprecipitated co-expressed Flag-CAT2 and Flag-NCA1 (Supplementary Fig. 7B, C). LCI assays in *N. benthamiana* epidermal leaf cells further indicated that CAIR1 interacts with both CAT2 and NCA1 (Fig. 1F). By contrast, no interactions were identified in the LCI assays for negative control combinations of CAIR1 with RUP2, or CAT2 and NCA1 with either UVR8 or RUP2, although UVR8 and RUP2 exhibited strong interaction, thus supporting functional UVR8 and RUP2 expression (Fig. 1F). Altogether, we conclude that Arabidopsis CAIR1 interacts and forms stable complexes with catalases and their chaperone NCA1.

### CAIR1 localizes to the cytosol, where it interacts with CAT2

**Fig. 2:**
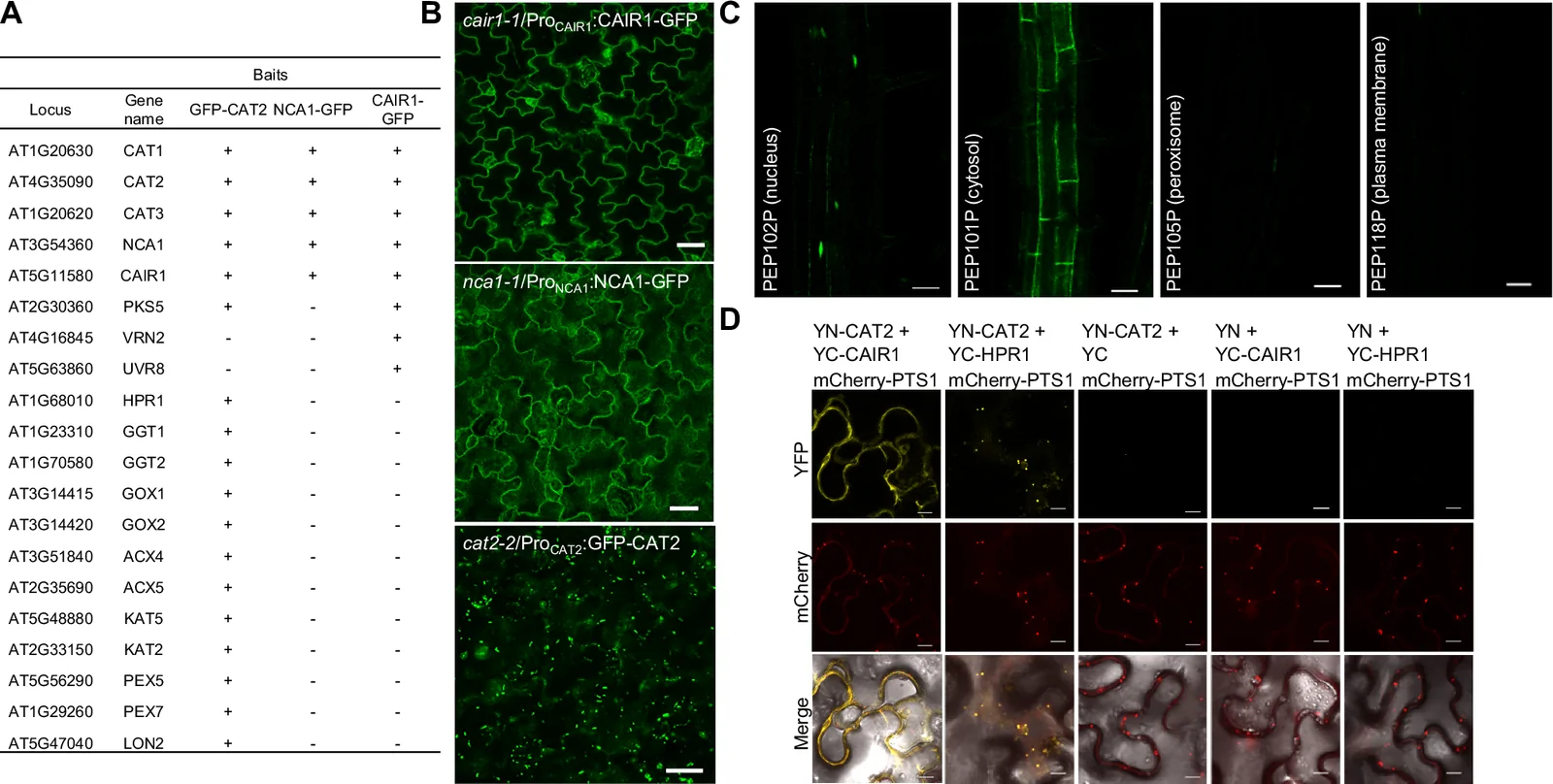
CAIR1 localizes to the cytosol where it interacts with CAT2. **A** List of proteins identified in the purified GFP-CAT2, NCA1-GFP, and CAIR1-GFP complexes. Representation of peptide peaks of different subunits measured by LC-MS/MS (Supplementary Data 1) is indicated schematically (+). **B** Subcellular localization of CAIR1-GFP, NCA1-GFP, and GFP-CAT2 in stably transformed 5-d-old Arabidopsis seedlings. Scale bars: 30 μm. **C** CAIR1 localizes to nuclei and cytosol but not to peroxisomes or plasma membrane. CAIR1 fused with sfGFP^11^ at the C-terminus was introduced into transgenic Arabidopsis expressing sfGFP1-10^OPT^ targeted to peroxisomes (PEP105P), nuclei (PEP102P), cytosol (PEP101P), and plasma membrane (PEP118P). GFP signals were examined using confocal laser scanning microscopy. Scale bar: 30 μm. **D** BiFC assay in *N. benthamiana* leaves transiently expressing split-YFP fusion proteins YFPN-CAT2 (YN-CAT2), YFPC-HPR1 (YC-HPR1), and YFPC-CAIR1 (YC-CAIR1). Depicted are YN-CAT2–YC-HPR1 interaction in peroxisomes, and YN-CAT2–YC-CAIR1 interaction in the cytosol, together with empty vector control combinations (YN, YC). The peroxisomal maker mCherry-PTS1 was co-expressed using Pro_35S_:mCherry-PTS1(SKL). Scale bars: 10 μm. **B**–**D** Representative data from three independent repetitions.

In addition to showing that CAIR1 forms a complex which includes catalases and NCA1 (Fig. 2A, Supplementary Data 1), both NCA1-GFP and GFP-CAT2 AP–MS provided a comprehensive insight into the CAT2 interactome. For AP–MS of GFP-CAT2, a peroxisomal marker line (Pro_35S_:GFP-PTS1)^60^ was used as an additional negative control next to wild type and a YFP-expressing line to reliably and robustly identify CAT2 interactors, including those in the peroxisome. In total, 82 proteins were enriched in GFP-CAT2 purifications compared with wild type, YFP, and GFP-PTS1 controls, many of which have been experimentally shown to localize to peroxisomes or possess a putative PTS (Supplementary Fig. 8A, Supplementary Data 1 and 3). Next to the top co-purifying proteins CAIR1, NCA1, CAT1, and CAT3, as based on fold-enrichment, several previously known CAT2 interactors were identified in the GFP-CAT2 AP–MS analysis, including PEX5^19,61^, ACYL-COA OXIDASE 4 (ACX4) and ACX5^62^, GLYCOLATE OXIDASE 1 (GOX1), GOX2^63,64^, and *S*-NITROSOGLUTATHIONE REDUCTASE (GSNOR1)^65,66^ (Fig. 2A). In addition, we identified the peroxisomal HYDROXYPYRUVATE REDUCTASE 1 (HPR1) as a potential, previously undocumented interactor of CAT2 (Fig. 2A). While GFP-CAT2 co-immunoprecipitated endogenous HPR1 as well as CAIR1 (Supplementary Fig. 8B), neither CAIR1-GFP nor NCA1-GFP pulled down HPR1, despite robust co-IP of catalases (Supplementary Fig. 8C, D). These results are consistent with the AP–MS data and the subcellular localizations of these proteins, and they suggest that CAIR1 may interact specifically with non-peroxisomally localized catalases. It is moreover noteworthy that AP–MS of CAIR1-GFP co-immunoprecipitated CATs and UVR8, but UVR8 and CAT could not be found in GFP-CAT2 and GFP-UVR8 AP–MS, respectively, despite clear co-IP of CAIR1 in both cases (Fig. 2A and Supplementary Fig. 1D), suggesting the absence of a ternary UVR8-CAIR1-CAT protein complex.

Although catalase activity and localization have been found in various cellular compartments, including the cytosol, mitochondria, chloroplasts, and nucleus, catalases are predominantly localized in peroxisomes^6,15,28^. Native promoter–driven CAIR1-GFP displayed cytosolic localization, mirroring the cytosolic localization of NCA1-GFP (Fig. 2B). Despite the demonstrated interaction of CAT2 with both CAIR1 and NCA1, GFP-CAT2 exhibited primarily peroxisomal localization (Fig. 2B). To further investigate the subcellular localization of CAIR1 with higher sensitivity, we used the self-assembling split superfolder GFP (sfGFP^OPT^) system^28,67^. The co-expression of CAIR1-sfGFP11 with sfGFP1-10 β-strand (sfGFP1-10^OPT^) targeted to the cytosol and nucleus resulted in reconstituted sfGFP fluorescence signals detectable by confocal laser scanning microscopy, which were not observed with peroxisome and plasma membrane-targeted sfGFP1-10^OPT^ (Fig. 2C), suggesting that CAIR1 is mainly localized to the cytosol and nucleus. It is of note that, in addition to NCA1 cytosolic and catalase peroxisomal localization, nuclear localization has also been described for both^28,41^. Thus, to reveal the subcellular localization of the CAIR1–CAT2 interaction, we used the bimolecular fluorescent complementation (BiFC) assay^68^ in transiently transformed *N. benthamiana* epidermal leaf cells. Reconstituted cytosolic YFP signals were detected when YC-CAIR1 and YN-CAT2 were co-expressed, indicating interaction of CAIR1 and CAT2 in the cytosol; however, YFP signal indicating interaction was not observed in nuclei or peroxisomes (Fig. 2D). This specific cytosolic interaction was further supported by comparing it to the sharply contrasting localization of YN-CAT2–YC-HPR1 interaction detected only in peroxisomes (Fig. 2D). We conclude that, whereas HPR1 identified in our GFP-CAT2 AP–MS is a previously undescribed interactor of CAT2 in peroxisomes, the CAIR1 interaction with CAT2 is—like the NCA1–CAT2 interaction—confined to the cytosol.

### *cair1* mutants show reduced catalase activity associated with catalase aggregation

**Fig. 3:**
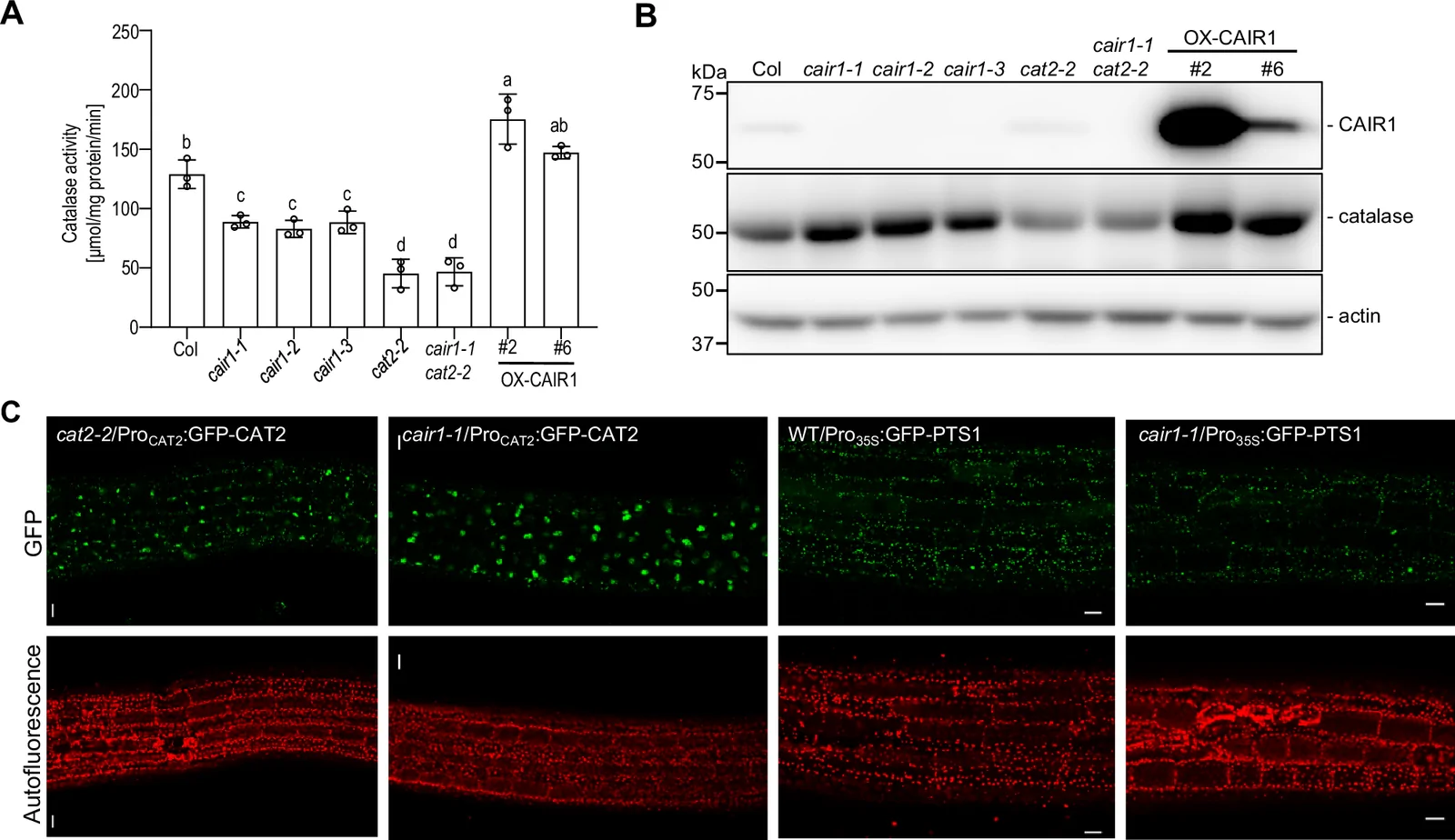
*cair1* show low catalase activity, associated with catalase aggregation. **A** Catalase activity in 5-d-old seedlings of wild type (Col), *cair1-1*, *cair1-2*, *cair1-3*, *cat2-2*, *cair1-1* *cat2-2*, and two CAIR1 complementation lines (OX-CAIR1 #2 and #6). Data shown are means ± SD (*n* = 3 biological replicates). Shared letters indicate no significant differences among means (*P* > 0.05; two-way ANOVA, Tukey’s test). **B** Immunoblot analysis of catalase protein levels in 5-d-old Col, *cair1-1*, *cair1-2*, *cair1-3*, *cat2-2*, *cair1-1 cat2-2*, and OX-CAIR1 #2 and #6 seedlings. Actin protein levels are shown as a loading control. **C** GFP-CAT2 forms aggregates in peroxisomes when expressed in the *cair1-1* mutant background, but not in *cat2-2*. GFP-PTS1 serves as a control. The GFP signal was investigated in 7-d-old seedlings. Scale bars: 30 μm. This experiment was performed three times with similar results.

Given the clear CAIR1–CAT interaction, we tested whether CAIR1 affects catalase activity. Indeed, catalase activities of 5-d-old *cair1-1, cair1-2*, and *cair1-3* seedlings were reduced when compared to those in wild type and two complementation lines (Fig. 3A). In contrast to *nca1*^41^, the reduced catalase activities in the *cair1* mutant alleles were not associated with altered catalase protein levels (Fig. 3B and Supplementary Fig. 3C, D). Thus, to better understand the reason for reduced catalase activity, we further assessed whether CAIR1 affected CAT2 subcellular localization. Interestingly, GFP-CAT2 formed aggregates in the *cair1* background but not in *cat2* complementation lines (Fig. 3C). In contrast, the peroxisome marker GFP-PTS1 showed no difference in subcellular localization when expressed in *cair1* versus in wild type (Fig. 3C). We conclude that CAIR1 interaction with CAT2 in the cytosol ensures proper peroxisomal localization of CAT2.

### *cair1* mutants show hypersensitivity to photorespiration stress and to alkaline growth conditions

Plant catalases have key roles in detoxifying H_2_O_2_ generated during photorespiration and fatty acid β-oxidation in peroxisomes^15^. *cat2* mutants conditionally accumulate elevated H_2_O_2_ levels, which leads to activation of cell death in leaves, particularly when grown under long-day (LD) conditions^69,70^. In contrast to *cat2*, neither *cair1* mutants nor two *CAIR1*-overexpression lines (*cair1-1*/Pro_35S_:CAIR1; OX-CAIR1) displayed noticeable leaf lesions when grown in LD (Supplementary Fig. 9A), a *cat2* mutant phenotype under LD associated with oxidative stress-induced salicylic acid (SA) signaling^71^. Although macroscopic leaf lesions could not be detected in *cair1*, *cair1* and *cair1 cat2* showed elevated expression of the SA marker genes *PR1* and *PR2* under LD in comparison to wild type and *cat2*, respectively (Supplementary Fig. 9B). Interestingly, however, under LD conditions with acute photorespiration stress, such as when 2-week-old plants grown in LD under high CO_2_ that inhibited photorespiration were transferred to air with ambient CO_2_ levels, *cair1* enhanced the *cat2* phenotype (Supplementary Fig. 9C–E). Indeed, *cair1 cat2* displayed leaf lesions after 3 d following transfer to ambient CO_2_, whereas *cat2* leaf lesions were only detectable after 7 d (Supplementary Fig. 9D, E). It is also noteworthy that the *cair1 cat2* double mutant displayed a smaller rosette size than *cat2* (Supplementary Fig. 9D, E), suggesting reduced CAT1 and CAT3 activity in the absence of CAIR1.

**Fig. 4:**
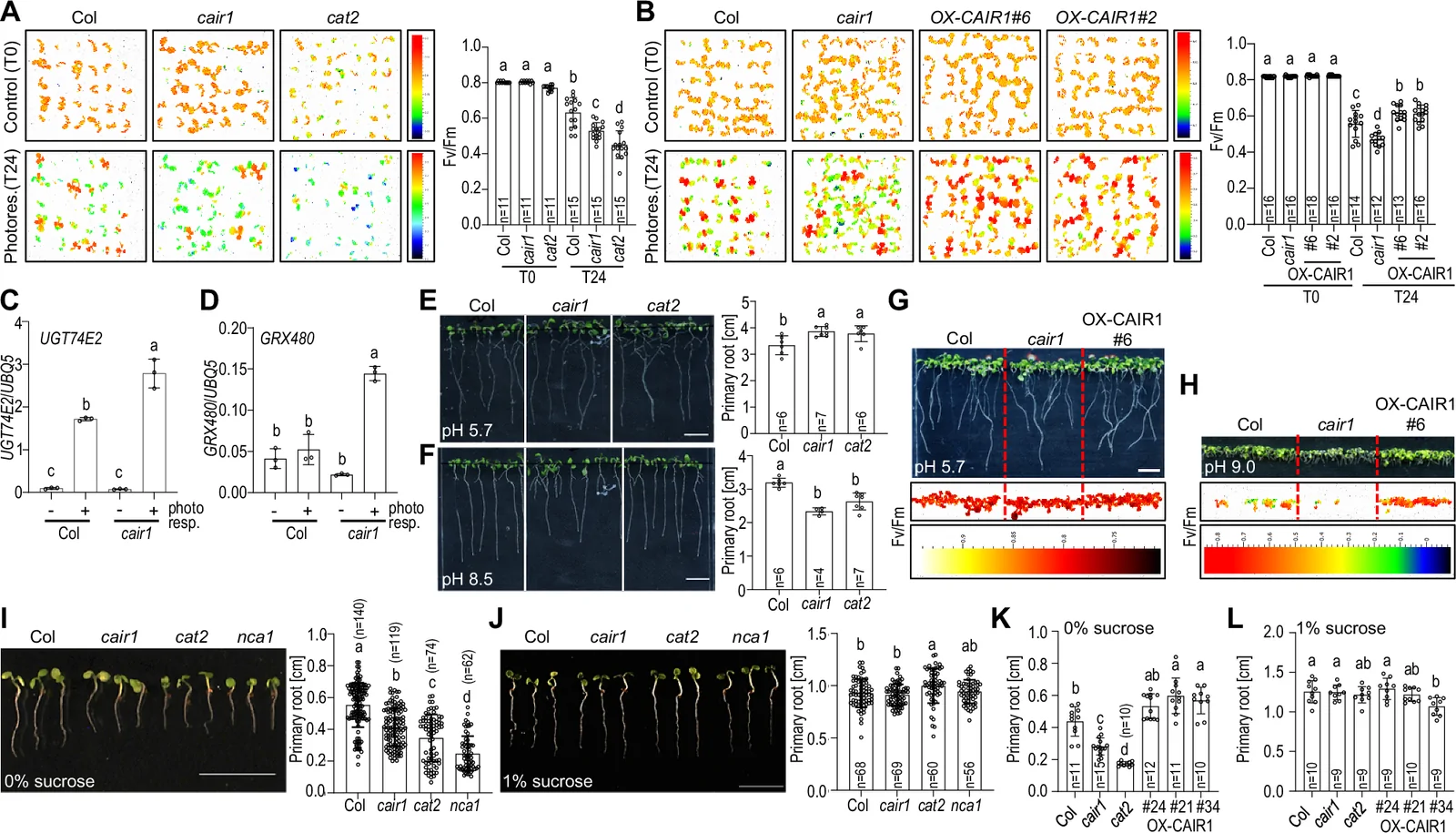
*cair1* is hypersensitive to enhanced photorespiration and alkaline pH. **A, B** Response to photorespiration–promoting conditions (gas limitation). (*Left*) Representative false-color *F*v/*F*m images of 10-d-old wild-type (Col), *cair1-1* (*cair1*), *cat2-2* (*cat2*), and *cair1-1*/Pro_35S_:CAIR1 overexpression lines (OX-CAIR1 #6 and #2) before (T0) and after 24 h of treatment (T24). Blue and red indicate low and high *F*v/*F*m, respectively. (*Right*) Quantitative *F*v/*F*m measurements (*n* ≥ 11 plants). **C, D** RT-qPCR analysis of *UGT74E2* and *GRX480* expression 7-d-old LD–grown Col and *cair1* seedlings after 24 h under control (−) or enhanced photorespiration (+) conditions (*n* = 3 biological replicates). Primary root length at pH 5.7 (**E**) or pH 8.5 (**F**). Col, *cair1*, and *cat2* seedlings were grown for 5 d on ½ MS containing 1% sucrose (pH 5.7), transferred to medium at pH 5.7 or 8.5, and analyzed 5 d (images) or 8 d (length, *n* ≥ 4 roots) after transfer. Scale bars: 1 cm. *cair1* is hypersensitive at pH 9.0. Five-d-old Col, *cair1*, and OX-CAIR1 #6 seedlings were transferred from ½ MS medium with 1% sucrose (pH 5.7) to medium at pH 5.7 (**G**) or pH 9.0 (**H**). Representative images (*top*) and false-color *F*v/*F*m images (*middle*) were acquired 5 d after transfer. Scale bars: 0.5 cm. *cair1* and *cat2* exhibit sucrose-dependent root elongation. Seedlings were grown for 5 d in LD on ½ MS medium without (**I**) or with (**J**) 1% sucrose. Representative images and root length measurements are shown (*n* ≥ 56 roots). **K, L** CAIR1-overexpressing lines (OX-CAIR1 #24, #21, and #34) have longer primary roots than Col, *cair1*, and *cat2* on sucrose-free medium (*n* ≥ 9 roots). **A**–**L** Data are individual biological replicates with means ± SD. Representative images are from three independent experiments. Shared letters indicate no significant differences among means (*P* > 0.05; two-way ANOVA, Tukey’s test).

We further assessed if CAIR1 has a functional role under enhanced photorespiration and associated oxidative stress. We measured the maximum quantum yield of photosystem II (*F*v/*F*m) before and after 1 d of gas limitation (resulting in low CO_2_/high O_2_ conditions), causing enhanced photorespiration. Although less pronounced than *cat2*, *cair1* mutants displayed reduced *F*v/*F*m relative to the wild type under 24-h gas exchange limitation conditions that enhanced photorespiration (Fig. 4A), whereas two CAIR1-overexpression lines conversely showed slightly higher *F*v/*F*m than wild type (Fig. 4B). Moreover, *cair1 cat2* showed further reduced *F*v/*F*m relative to *cat2* under photorespiration-promoting conditions (Supplementary Fig. 10A, B). In agreement with enhanced oxidative stress, *cair1* showed enhanced expression of two H_2_O_2_-inducible marker genes, namely *UGT74E2* and *GRX480*^72^, after 24 h of enhanced photorespiration (Fig. 4C, D).

*nca1* mutants are more sensitive to alkaline growth conditions (high external pH) that induce the production of ROS in different cellular compartments, including peroxisomes^42^. To investigate this sensitivity in wild-type, *cair1*, and *cat2*, we transferred 4-d-old seedlings from pH 5.7 growth medium to pH 8.5 growth medium. Like *nca1-1*^42^, *cat2*, and *cair1* showed reduced primary root growth compared to that in wild type at pH 8.5, which did not occur at pH 5.7 (Fig. 4E, F). Moreover, at the higher pH of 9.0, survival of *cair1* was reduced in comparison to wild type (determined by absence of *F*v/*F*m measurements), whereas a CAIR1-overexpressing line showed enhanced alkalinity tolerance in the same conditions (Fig. 4G, H). We conclude that, as a positive regulator of catalase activity, CAIR1 contributes to resilient growth under alkaline conditions.

During seedling establishment, fatty acid β-oxidation in peroxisomes generates H_2_O_2_ that must be efficiently removed by catalase activity. On sucrose-free growth medium where fatty acid β-oxidation must fuel growth, the loss of CAT2 function results in reduced root elongation^33,73^. We thus compared the growth of wild type, *cat2*, *nca1, cair1*, and OX-CAIR1 seedlings on sucrose-free and sucrose-supplemented medium. After 5 d, *cat2*, *nca1*, and *cair1* seedlings exhibited significantly shorter primary roots than wild type, specifically in the absence of sucrose (Fig. 4I, J), whereas CAIR1-overexpressing lines showed longer primary roots under these conditions (Fig. 4K, L). By contrast, exogenous sucrose fully restored root growth in all mutants (Fig. 4J, L). These results further indicate that CAIR1 is required for catalase activity to tolerate H_2_O_2_ generated during fatty acid β-oxidation.

### CAIR1 is an unstable protein under oxidative stress

**Fig. 5:**
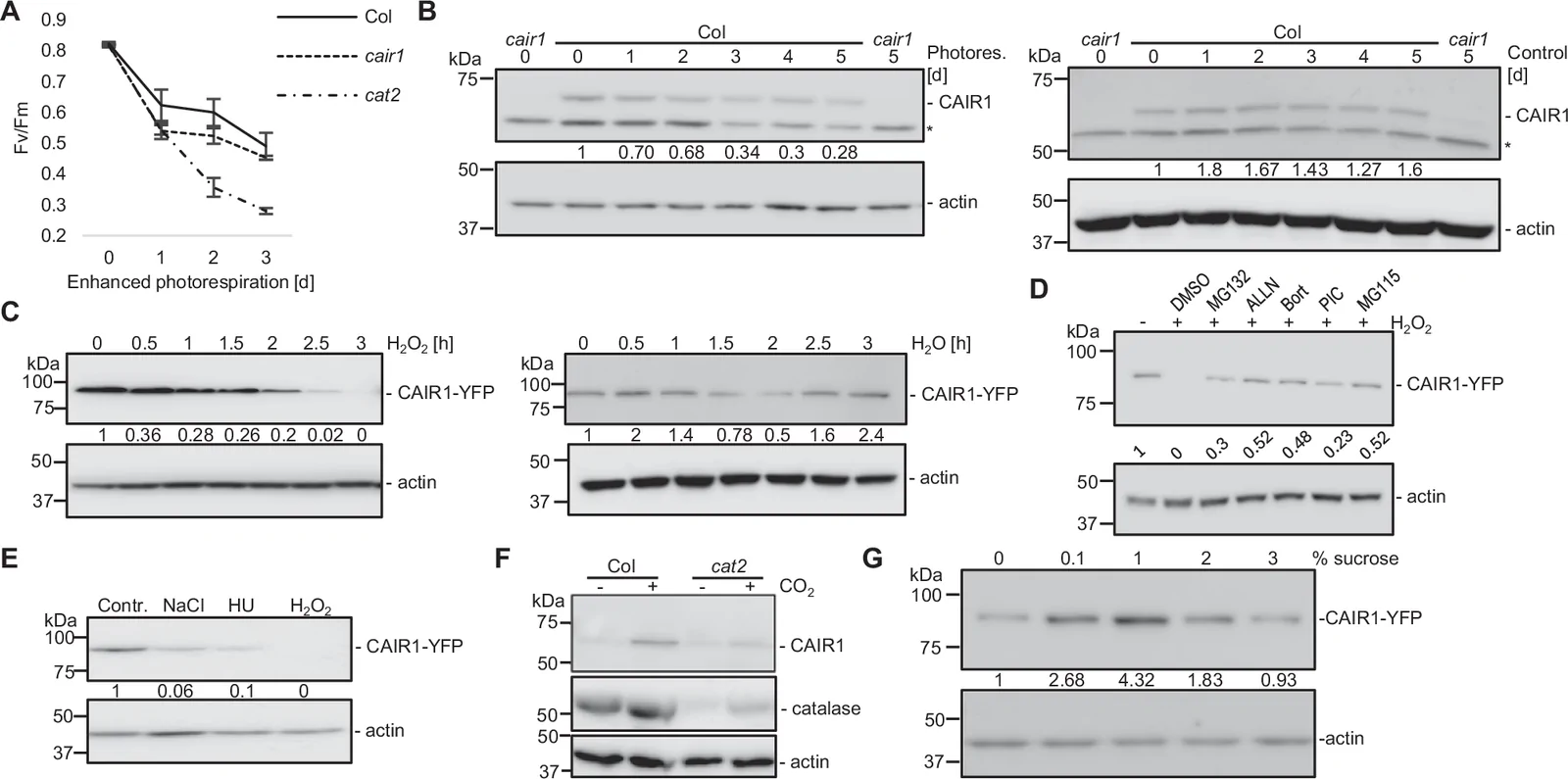
Proteasomal degradation of CAIR1 in response to ROS. **A** *cair1* mutant is sensitive to short-term enhanced photorespiration. *F*v/*F*m values of 10-d-old Col, *cair1-1* (*cair1*), and *cat2-2* (*cat2*) seedlings grown under long-day (LD) conditions following transfer to enhanced photorespiration conditions for the indicated time period [d]. Means ± SD are shown (*n* = 3 plants). **B** Endogenous CAIR1 proteins are degraded after 3 d of enhanced photorespiration treatment. Immunoblot analysis of endogenous CAIR1 in 6-d-old Col and *cair1* seedlings following their transfer from a CO_2_-enriched chamber to conditions promoting enhanced photorespiration (Photores. [d]), or not (Control). **C** Exogenous H_2_O_2_ leads to destabilization of CAIR1-YFP protein. Immunoblot analysis of CAIR-YFP in 3-d-old *cair1-1*/Pro_35S_:CAIR1-YFP #23 seedlings following their transfer to water supplemented with 20 mM H_2_O_2_ or water only (mock control) for the indicated time [h]. **D** Immunoblot analysis of CAIR-YFP in 3-d-old Pro_35S_:CAIR1-YFP seedlings that were pre-treated with 200 µM MG132, ALLN, bortezomib (Bort), protease inhibitor cocktail tablets (PIC), MG115, or DMSO (mock control) for 4 h in water followed by treatment with 20 mM H_2_O_2_ for 3 h (+ H_2_O_2_). **E** Salt stress, hydroxyurea, and H_2_O_2_ treatments similarly destabilize CAIR1-YFP. Immunoblot analysis of CAIR-YFP in 3-d-old Pro_35S_:CAIR1-YFP seedlings following their transfer to liquid MS medium supplemented with 100 mM NaCl (NaCl), 3 mM HU (HU), or 20 mM H_2_O_2_ (H_2_O_2_) compared to straight liquid MS (Contr.) for 3 h. **F** CO_2_ supplementation stabilizes CAIR1 levels. Immunoblot analysis of CAIR1 and catalase in 5-d-old Col and *cat2* seedlings grown in LD with (+) or without (−) CO_2_ supplementation. **G** The presence of sucrose stabilizes CAIR1-YFP protein level and inhibits oligomerization of CAIR1-YFP. Immunoblot analysis of CAIR-YFP in 6-d-old Pro_35S_:CAIR1-YFP seedlings cultured in media containing various sucrose concentrations (%). **B**–**G** Actin was used as loading control.

Chlorophyll fluorescence (*F*v/*F*m) measurements comparing oxidative stress responses of *cair1* and wild type suggested that CAIR1 played a functional role mainly during the first 24–48 h of enhanced photorespiration (Fig. 5A). Interestingly, extended exposure to enhanced photorespiration as well as exogenous H_2_O_2_ treatment led to the degradation of endogenous CAIR1 (Fig. 5B, C), which was blocked with proteasome inhibitors (Fig. 5D). Moreover, we assessed the impact of additional stress factors on CAIR1 stability and observed that both salt and hydroxyurea (HU; a catalase inhibitor)^74^ treatments destabilized CAIR1 (Fig. 5E). By contrast, exogenous sucrose up to 2%^73^ and CO_2_ supplementation, conditions known to reduce ROS production, both led to CAIR1 stabilization (Fig. 5F, G). Taking these data together, we thus conclude that ROS destabilizes CAIR1 through proteasomal degradation.

### Redox-dependent oligomerization of CAIR1 modulates its interaction with catalases

**Fig. 6:**
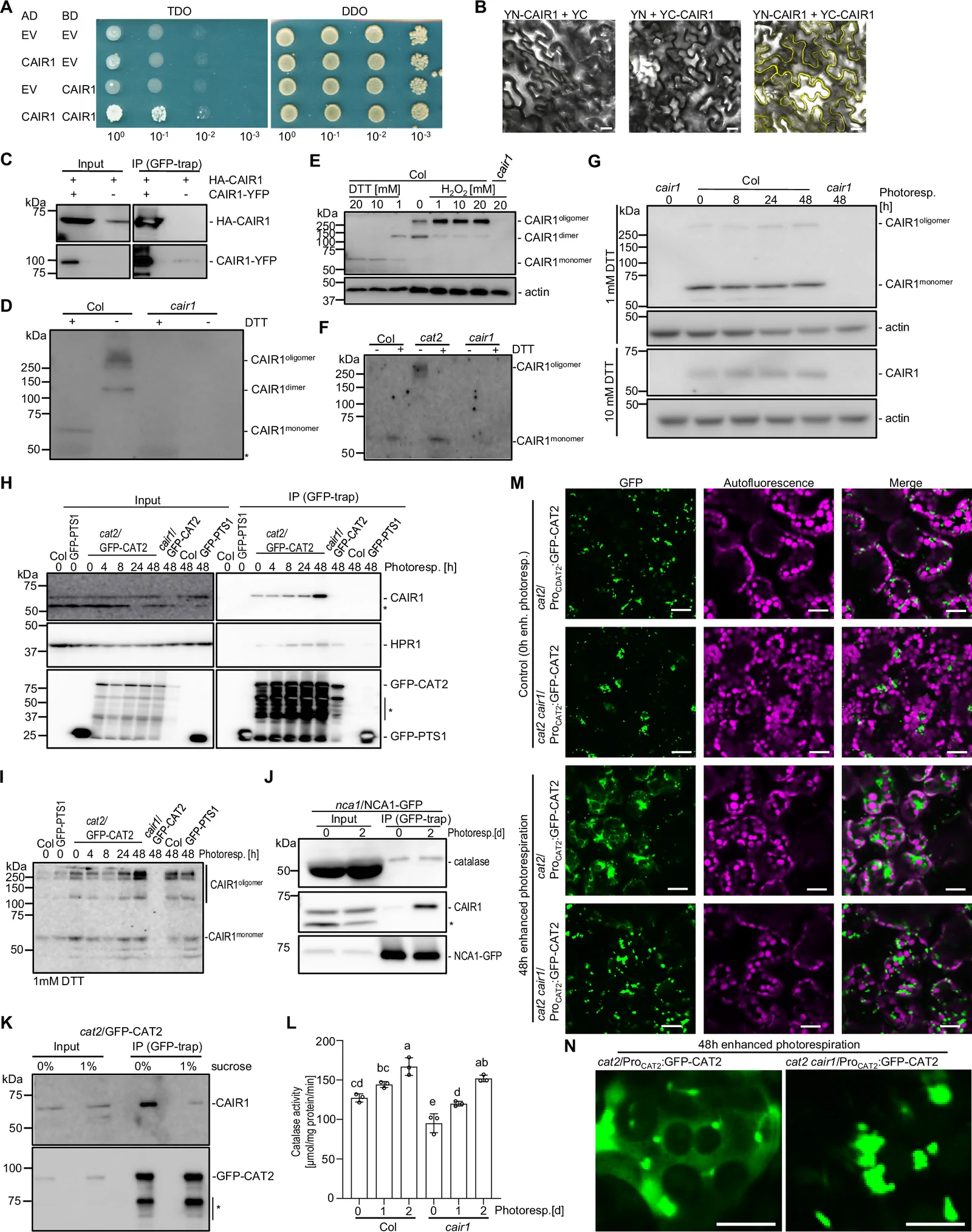
ROS promotes CAIR1 oligomerization and regulates its interaction with catalase. **A** Yeast two-hybrid assay showing CAIR1 self-interaction. Serial dilutions of transformed yeast spotted on SD/−Trp/−Leu (DDO; nonselective for interaction) and SD/−Trp/−Leu/−His (TDO; selective). AD, activation domain; BD, DNA-binding domain; EV, empty vector. **B** BiFC assay in *N. benthamiana* leaves showing cytosolic interaction between YN-CAIR1 and YC-CAIR1. Scale bars: 30 μm. **C** Co-IP assay using Pro_35S_:CAIR1-YFP and Pro_35S_:3xHA-CAIR1 showing CAIR1 self-interaction. **D, E** Immunoblot analysis showing redox-dependent CAIR1 oligomerization. Proteins from 5-d-old Col and *cair1* seedlings were extracted with (+) or without (−) 5 mM DTT (**D**), or treated with the indicated concentrations of DTT or H_2_O_2_ (**E**). Actin served as a loading control. **(F)** Increased CAIR1 oligomerization in *cat2* compared with Col in LD. Protein extractions performed with (+) or without (–) DTT. **G** Enhanced photorespiration promotes CAIR1 oligomerization. Col and *cair1* seedlings were treated for the indicated times before protein extraction with 1 or 10 mM DTT. **H, I** ROS enhances the interaction between CAIR1 and CAT2. Co-IP of CAIR1 and HPR1 with GFP-CAT2 from 10-d-old *cat2*/Pro_CAT2_:GFP-CAT2 seedlings exposed to enhanced photorespiration for the indicated durations (**H**), with corresponding CAIR1 oligomerization shown in (**I**). Proteins were extracted without DTT. **J** Co-IP assay showing enhanced interaction between NCA1 and CAIR1, but not between NCA1 and catalases, following 2 d of enhanced photorespiration Proteins were extracted without DTT from 10-d-old *nca1-1*/Pro_NCA1_:NCA1-GFP. **K** Sucrose reduces the interaction between CAIR1 and CAT2. Co-IP was performed using 5-d-old *cat2*/GFP-CAT2 seedlings grown on 0% or 1% sucrose. Proteins were extracted without DTT. **L** Catalase activity in 5-d-old Col and *cair1* seedlings after 0, 1, or 2 d of enhanced photorespiration. Means ± SD are shown (*n* = 3 biological replicates). Shared letters indicate no significant differences among means (*P* > 0.05; two-way ANOVA, Tukey’s test). **M, N** Confocal imaging of GFP-CAT2 localization in 12-d-old *cat2*/Pro_CAT2_:GFP-CAT2 and *cat2 cair1*/Pro_CAT2_:GFP-CAT2 seedlings exposed or not to 2 d of enhanced photorespiration. Representative data of three independent repetitions. **N** Higher magnification of a subpart in (**M**). Scale bars: 20 µm (**M**), 10 µm (**N**). **H**, **K** Asterisks indicate degradation products.

In Y2H and BiFC assays, CAIR1 showed interaction with itself, suggesting that CAIR1 oligomerizes in vivo (Fig. 6A, B). Indeed, HA-CAIR1 co-immunoprecipitated with CAIR1-YFP in extracts from a Pro_35S_:CAIR1-YFP Pro_35S_:HA-CAIR1 double transgenic line (Fig. 6C). Interestingly, anti-CAIR1 immunoblot analysis of extracts prepared under non-reducing conditions in the absence of dithiothreitol (DTT) detected bands at sizes approximate to that of CAIR1 dimers and higher order oligomeric forms that were absent in *cair1* (Fig. 6D). Based on this observation, we further investigated whether CAIR1 oligomerization is dependent on its redox status by treating protein extracts from 5-d-old seedlings with varying concentrations of DTT as a reducing agent and H_2_O_2_ as an oxidizing agent. Notably, only the monomeric form of CAIR1 was detected in the presence of 10 and 20 mM DTT (Fig. 6E). However, as oxidative conditions intensified, higher order oligomeric forms of CAIR1 emerged (at sizes approximate to that of homodimeric and -tetrameric forms), indicating that CAIR1 oligomerization is redox-dependent (Fig. 6E). This response was specific for CAIR1, since, in sharp contrast to YFP-CAIR1, no redox-dependent oligomerization could be observed for YFP-HA, HPR1, GFP-CAT2, NCA1-GFP, or the related RCC1-like family members GFP-UVR8 and YFP-AT5G16040 (Supplementary Fig. 11A–C). Consistent with its elevated endogenous ROS levels, *cat2* displayed enhanced CAIR1 oligomerization compared to that in wild type (Fig. 6F). Similarly, enhanced photorespiration, which is associated with elevated levels of endogenous ROS, also increased CAIR1 oligomerization (Fig. 6G).

We further investigated whether redox-dependent oligomerization of CAIR1 influences its interaction with CAT2. Indeed, enhanced photorespiration increased interaction of CAIR1 with GFP-CAT2 and NCA1-GFP as determined by co-IP assays (Fig. 6H-J), but did not affect the interaction between NCA1-GFP and catalases (Fig. 6J). Interestingly, the interaction of GFP-CAT2 with HPR1 also increased under these conditions (Fig. 6H). We also found that exogenous sucrose, which reduces ROS production during seedling establishment^73^, significantly inhibited the interaction of GFP-CAT2 with CAIR1 (Fig. 6K). We then assessed whether catalase activity is altered under growth conditions associated with elevated ROS levels. Catalase activity indeed increased under enhanced photorespiration in both wild type and *cair1*, although overall catalase activity remained lower in *cair1* (Fig. 6L). Moreover, incubation under enhanced photorespiration conditions for 2 d resulted in the CAIR1-dependent, altered subcellular localization of GFP-CAT2, manifesting as enhanced cytosolic localization (Fig. 6M, N). Our findings suggest that ROS reversibly regulates CAIR1 oligomerization state and its interaction with catalases, which affects catalase subcellular localization.

### Cys-356 and Cys-545 contribute to redox-regulated CAIR1 oligomerization and function

**Fig. 7:**
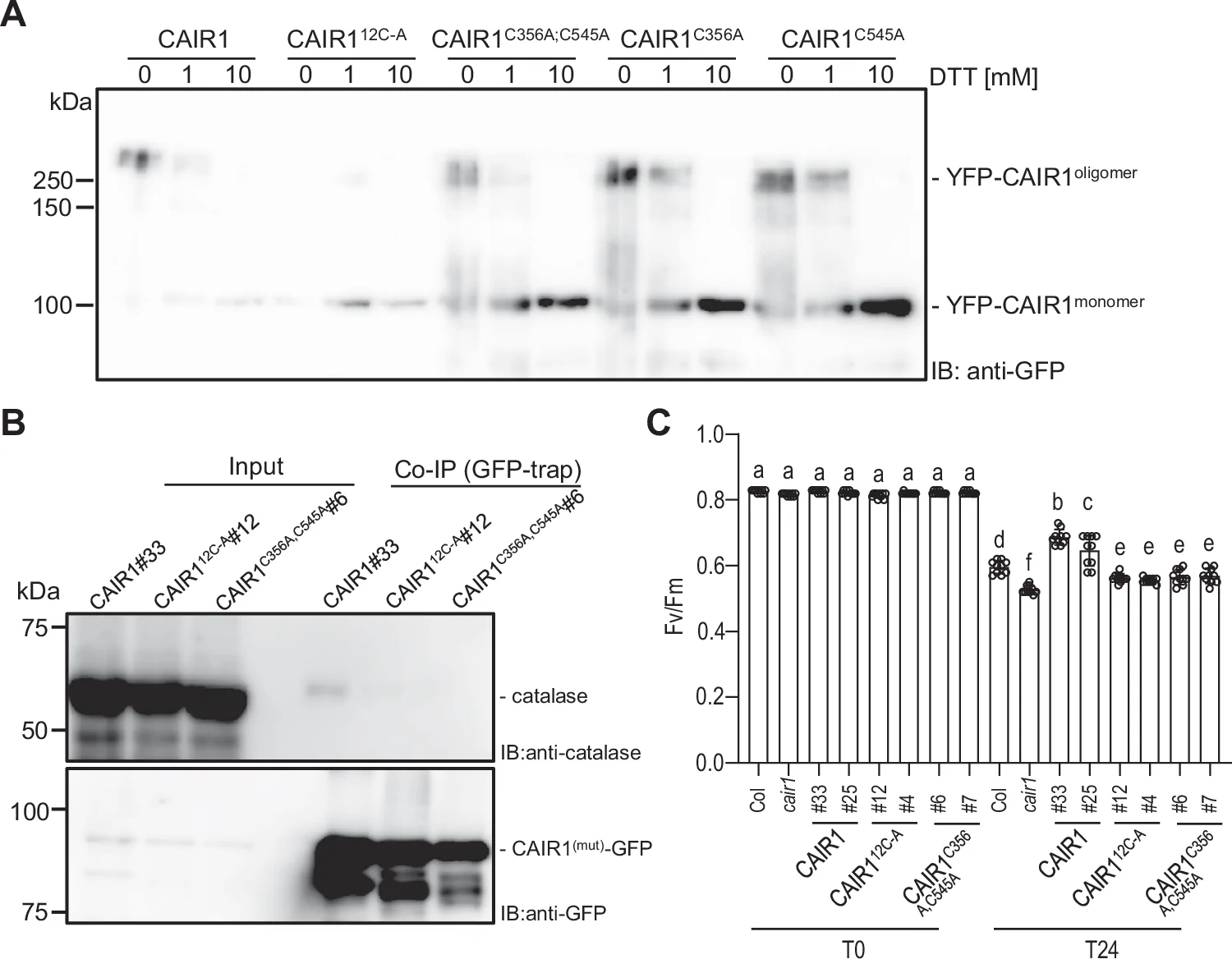
Cys-356 and Cys-545 contribute to CAIR1 function. **A** Immunoblot analysis of YFP-CAIR1, showing CAIR1 oligomeric state in 5-d-old seedlings expressing the CAIR1 variants CAIR1^12C-A^, CAIR1^C356A;C545A^, CAIR1^C356A^, or CAIR1^C545A^ with a YFP-tag under control of the 35S-promoter in the *cair1-1* background, with protein extractions performed with different concentrations of DTT. **B** Co-IP assay of catalase using GFP-trap purification, showing that cysteine mutation at C356 and C545 disturbs ROS-dependent interaction of CAT2 and CAIR1. Proteins were extracted from 10-d-old *cair1-1*/Pro_35S_:YFP-CAIR1 #33 (CAIR1#33), *cair1-1*/Pro_35S_:YFP-CAIR1^C12-A^ #12 (CAIR1^C12-A^#12), and *cair1-1*/Pro_35S_:YFP-CAIR1^C356A,C545A^ #6 (CAIR1^C356A,C545A^#6) seedlings exposed to 2 d enhanced photorespiration conditions using lysis buffer lacking DTT. **C** The cysteine of CAIR1 at C356 and C545 are required for oxidative response. Response to photorespiration-promoting growth conditions. *F*v/*F*m values of 10-d-old wild-type (Col), *cair1-1* (*cair1*), *cair1-1*/Pro_35S_:YFP-CAIR1 lines #25 and #33 (CAIR1 #25 and #33), *cair1-1*/Pro_35S_:YFP-CAIR1^C12-A^ lines #4 and #12 (CAIR1^C12-A^ #12 and #4), and *cair1−1*/Pro_35S_:YFP-CAIR1^C356A,C545A^ lines #6 and #7 (CAIR1^C356A,C545A^ #6 and #7) seedlings before (T0) and after 24 h of photorespiration–promoting growth conditions (T24). Individual data points and means ± SD are shown (*n* = 10 plants). Shared letters indicate no significant differences among means (*P* > 0.05; two-way ANOVA, Tukey’s test).

ROS-induced redox changes can oxidize cysteine thiols (RSH/RS-), producing highly reactive sulfenic acids (RSOH). These intermediates can then react with additional thiol groups to form disulfide bonds, promoting redox-dependent homo- or hetero-oligomerization of proteins^75–78^. CAIR1 contains 12 cysteines (Supplementary Fig. 12A), and mutation of each of these to alanine (CAIR1^12C-A^) abolished redox-dependent oligomerization in *N. benthamiana* transient assays and reduced it in Arabidopsis, thus supporting CAIR1 oligomerization via disulfide bond-formation (Supplementary Fig. 12B, C). Among the 12 cysteines, AlphaFold3 structural analysis predicted 5 cysteines to be in the hydrophobic core and thus unlikely to be involved in redox regulation (C204, C270, C291, C330, C390), and 7 cysteines to be surface exposed (C36, C96, C116, C145, C356, C424, C545), with in particular Cys-356 and Cys-545 residues apparently well-conserved across plants (Supplementary Fig. 2C and 12D). Interestingly, mutating them both to alanine (CAIR1^C356A,C545A^) reduced CAIR1 oligomerization in response to elevated ROS, as demonstrated in Arabidopsis and in *N. benthamiana* transient expression assays (Fig. 7A and Supplementary Fig. 12E). However, CAIR1^12C-A^ with all 12 cysteines mutated to alanines still showed ROS-mediated CAIR1 degradation and sucrose-mediated CAIR1 stabilization (Supplementary Fig. 12F, G). We then tested whether cysteine-dependent oligomerization is required for the cellular redox-enhanced interaction between CAIR1 and catalase in Arabidopsis. In comparison to CAIR1, CAIR1^C356A,C545A^ and CAIR1^12C-A^ indeed exhibited reduced interaction with catalases under stress conditions (Fig. 7B). Furthermore, expressing CAIR1^12C-A^ or CAIR1^C356A,C545A^ in *cair1-1* only partially rescued the *cair1* mutant phenotype under enhanced photorespiration conditions (Fig. 7C). Together, these findings indicate that cellular redox status may regulate CAIR1 oligomerization involving Cys-356 and Cys-545, thereby modulating the CAIR1–catalase interaction and maintaining ROS homeostasis. However, ROS-mediated CAIR1 degradation occurs independently of its cysteine residues.

### CAIR1 mediates UV-B–induced inhibition of catalase activity

CAIR1, although identified as a previously undescribed interactor of the UVR8 photoreceptor, does not appear to influence UVR8-mediated UV-B-induced photomorphogenesis based on the standard phenotypic assays analyzed in this study. We therefore investigated whether UV-B alters CAIR1-mediated regulation of catalase activity. Wild-type plants grown in the presence of UV-B showed reduced catalase activity compared to those grown in absence of UV-B, whereas UV-B had no or a reduced effect on catalase activity in *cair1* and *uvr8* mutants, respectively (Supplementary Fig. 13A). The UV-B effect on catalase activity was independent of any detectable effect on catalase protein abundance (Supplementary Fig. 13B). However, plants grown under UV-B showed reduced interaction between CAIR1 and CAT2 in co-IP assays compared to plants grown in absence of UV-B (Supplementary Fig. 13C). Interestingly, both endogenous CAIR1 and OX-CAIR1 protein levels increased under UV-B treatment (Supplementary Fig. 13D, E). In contrast, CAIR1 and CAIR1-YFP oligomerized less under UV-B, and this reduction was partially dependent on UVR8 (Supplementary Fig. 13F, G). Collectively, these findings suggest that CAIR1 and UVR8 mediate UV-B–induced inhibition of catalase activity through suppression of the CAIR1–CAT2 interaction.

## Discussion

**Fig. 8:**
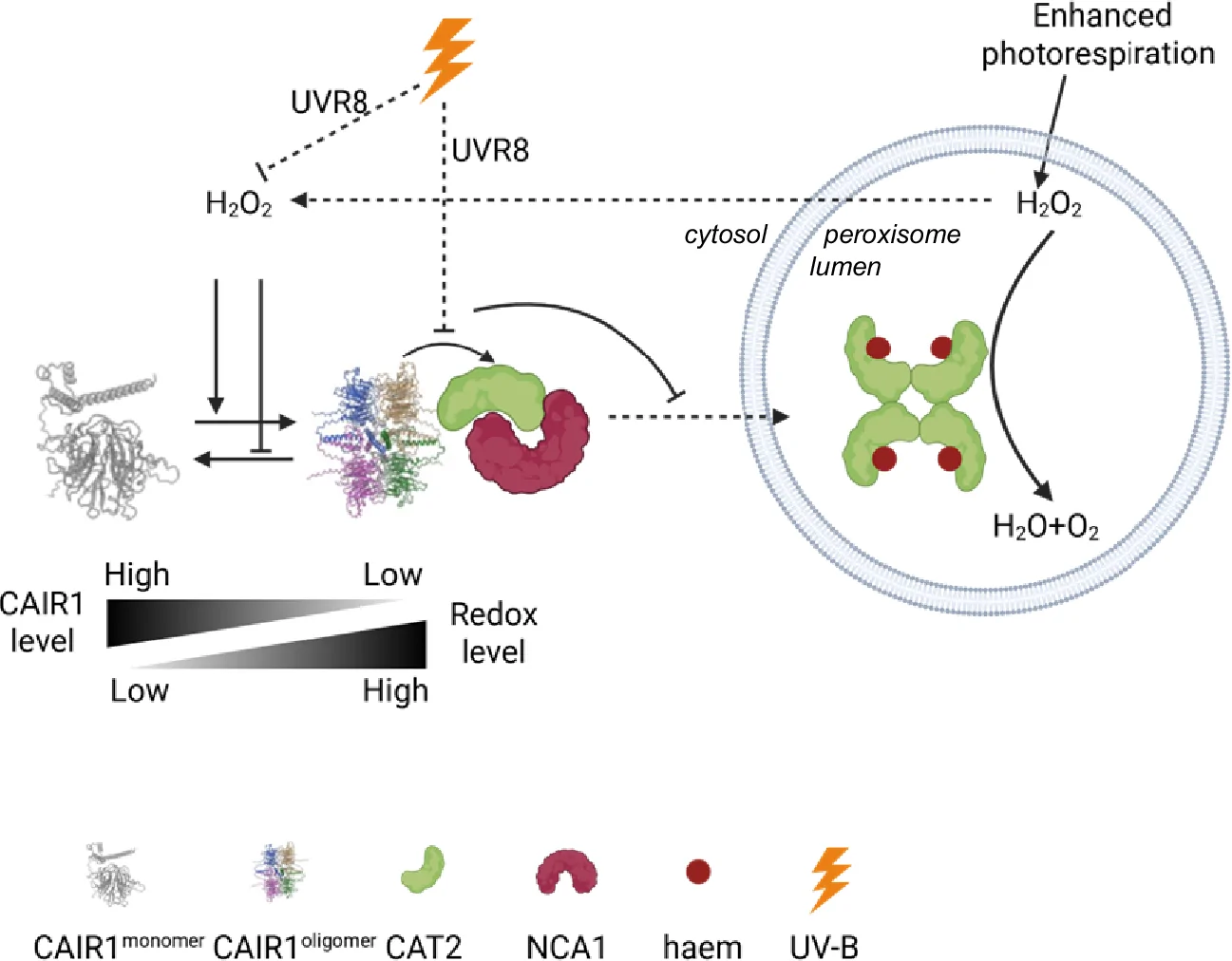
Working model of CAIR1-mediated regulation of catalase activity in Arabidopsis. Cellular ROS levels in Arabidopsis are tightly regulated through CAIR1 reversible oligomerization, which modulates catalase activity: (*Left of diagram*) Typically, catalase activity in peroxisomes converts potentially damaging H_2_O_2_ to benign H_2_O and O_2_. Elevated H_2_O_2_ in peroxisomes results from enhanced photorespiration. The mechanism by which H_2_O_2_ is transmitted between compartments, such as from peroxisomes to the cytosol or other organelles, remains unknown (indicated as a dashed arrow). Elevated H_2_O_2_ in the cytosol promotes CAIR1 oligomerization (indicated as CAIR1^oligomer^), as mediated by the C356 and C545 residues, and accelerates CAIR1 degradation. CAIR1 oligomerization enhances CAIR1 interaction with catalases (CAT2) and its chaperone NCA1 in the cytoplasm, thereby inhibiting catalase peroxisomal import and increasing catalase activity in the cytosol. (*Top right of diagram*) Under UV-B, UVR8 interaction with CAIR1 mediates inhibition of catalase activity by weakening the interaction between CAIR1 and CAT2. It remains unclear whether UV-B regulates ROS levels through UVR8 to indirectly suppress the CAIR1–CAT2 interaction, or whether UV-B directly inhibits the CAIR1–CAT2 interaction in a UVR8-dependent manner (as indicated by dashed inhibitory lines). Overall, these molecular interactions result in an inverse relationship between CAIR1 level and redox level in the cytosol, which is inversely regulated by UV-B-activated UVR8. Created in BioRender; Ulm, R. (2026), https://BioRender.com/g8xt8i9.

The interplay between ROS metabolism, peroxisomal function, and catalase activity is central to cellular redox homeostasis and stress responses. Catalases decompose H_2_O_2_ into water and molecular oxygen, and their function in peroxisomes controls cellular H_2_O_2_ levels. In this study, we identified CAIR1 through its co-purification with GFP-UVR8 by AP–MS. CAIR1 is a previously functionally uncharacterized Arabidopsis RCC1-Like protein family member that interacts with catalases and NCA1 in the cytosol and positively regulates catalase activity, likely by regulating catalase entry into peroxisomes. CAIR1 undergoes reversible redox-dependent oxidation involving Cys356 and Cys545, resulting in changes in its oligomeric state. This redox-controlled oligomerization modulates CAIR1–catalase interactions and thereby fine-tunes catalase activity in response to the cellular redox environment (Fig. 8).

Although the importance of catalases in peroxisomes is well established, a mechanistic understanding of their peroxisomal import remains incomplete. Plant catalases lack a canonical C-terminal PTS1 motif, yet their import depends on PEX5, the receptor for PTS1-containing matrix proteins^6,19,34^, which is consistent with our observation that PEX5 co-purified with GFP-CAT2 (Supplementary Data 1). Studies in fungi have shown that weak, non-canonical PTS1 signals delay peroxisomal import, thus allowing proper folding and preventing aggregation of catalase within the peroxisomal matrix^61^. The same may apply in plants, given that artificial addition of a canonical PTS1 to Arabidopsis CAT2 failed to fully restore catalase activity or mutant phenotypes^28^. Although plant non-canonical PTS1 signals differ from those in fungi and mammals and remain poorly defined^6^, the available evidence indicates that import kinetics are critical for catalase functionality.

Consistent with the model that catalase import kinetics are important, we observed enhanced aggregation of GFP-CAT2 in *cair1* mutants, whereas the localization of GFP-PTS1 was unaffected. This suggests that CAIR1 forms a cytosolic complex with CAT2 that regulates the rate of catalase import into peroxisomes, which appears essential for proper enzymatic activity. Notably, PEX5 was detected only in GFP-CAT2 AP–MS, but not in CAIR1-GFP or NCA1-GFP datasets, suggesting that CAIR1 and NCA1 transiently associate with CAT2 in the cytosol and thus delay its interaction with PEX5, preventing premature peroxisomal import. Thus, basal catalase activity likely depends on tightly controlled import kinetics mediated by CAIR1.

Our data indicate that the CAIR1–CAT2–NCA1 complex localizes to the cytosol, where NCA1 functions as a chaperone required for catalase folding and stability^40–42^. Interestingly, CAIR1 apparently does not affect the interaction of NCA1 with catalases, and catalase protein levels are not altered in *cair1* or a CAIR1-overexpression line. These observations suggest that CAIR1 plays no major role in NCA1-mediated catalase folding or stability but likely affects peroxisomal import subsequently; however, the exact molecular mechanism remains to be determined.

Under conditions leading to enhanced photorespiration, cytosolic accumulation of CAT2 was strictly CAIR1 dependent (Fig. 6M, N). This may reflect direct CAIR1-mediated retention of CAT2 in the cytosol under elevated ROS or indirect modulation of the peroxisomal import machinery. In support of redox-sensitive import control, PEX5 activity has been shown to decrease under oxidative stress in yeast^79,80^. Similarly, in mammalian cells, oxidative stress induces phosphorylation of Pex14, a core component of the peroxisomal translocation machinery, thus impairing Pex5–Pex14 interactions, suppressing catalase import, and increasing cytosolic catalase levels^26,29,30^. These observations suggest that regulated cytosolic localization of catalases is evolutionarily conserved, although the molecular mechanisms differ among organisms. How CAIR1 mechanistically mediates catalase retention in the cytosol, and the relative contributions of cytosolic versus peroxisomal catalase to oxidative stress tolerance in plants, remain open questions.

In addition to redox-dependent regulation, our data reveal that CAIR1 also mediates UV-B–induced inhibition of catalase activity. CAIR1 was identified in both the CAT2 and UVR8 complexes. However, CAT2 did not co-purify with GFP-UVR8, nor did UVR8 with GFP-CAT2, suggesting that CAIR1 interacts with UVR8 and CAT2 in a mutually exclusive manner. Although CAIR1 interacts with the UV-B photoreceptor UVR8, it does not appear to participate in canonical UVR8-dependent photomorphogenic responses. Instead, UV-B exposure suppresses catalase activity in a manner that requires both CAIR1 and UVR8, without substantially affecting catalase protein abundance. This reduction in activity is accompanied by a UV-B-induced decrease in the CAIR1–CAT2 interaction, suggesting that disruption of this complex is a key mechanism underlying catalase inhibition. Notably, UV-B treatment stabilizes CAIR1 protein levels and reduces CAIR1 oligomerization in a UVR8-dependent manner; however, the biological relevance of this remains presently unclear. Together, these findings identify a UV-B–responsive regulatory module in which UVR8 and CAIR1 collectively attenuate catalase activity. An important unresolved question is whether UV-B-activated UVR8 modulates cellular ROS homeostasis, such as via activation of antioxidant pathways, and indirectly weakens the CAIR1–CAT2 interaction, or whether UVR8 directly interferes with the CAIR1–CAT2 complex upon UV-B perception.

Beyond their cytotoxic effects, ROS function as key signaling molecules in plant development and stress responses^1,81^. Fluctuations in cellular redox status in response to environmental stresses affect protein functions through redox-dependent oxidation^4,76,82–84^. For example, activity and stability of redox-sensitive transcription factors are affected by the redox state of the cell^82,83,85–88^. Given its central role in H_2_O_2_ homeostasis, catalase localization and activity must be tightly regulated^89^. Our data indicate that CAIR1, together with the catalase chaperone NCA1, assembles into a cytosolic regulatory complex that fine-tunes catalase activity. Our findings suggest that CAIR1 regulation operates through two distinct redox-sensitive layers. First, under rising cellular H_2_O_2_ levels, CAIR1 responds to cellular redox changes, possibly through intermolecular disulfide bond formation involving Cys356- and Cys545, driving the formation of CAIR1 oligomers, thereby enhancing its interaction with catalases and subsequent peroxisomal import rates. Second, during prolonged oxidative stress, CAIR1 protein abundance decreases, suggesting that higher or chronic ROS levels might initiate post-translational destruction pathways, possibly involving oxidation-dependent ubiquitination. As a result, CAIR1 levels drop when ROS levels are excessive or sustained over time, limiting its ability to facilitate catalase activation. Together, these dual mechanism enables plants to dynamically adjust their H_2_O_2_ scavenging capacity and maintain redox homeostasis under fluctuating environmental conditions.

AP–MS–based interactome analysis further expands our understanding of the protein networks associated with catalases. Our GFP-CAT2 AP–MS confirmed known interactors and identified numerous further candidates, including many peroxisomal enzymes. This is consistent with early ultrastructural observations of enzyme-rich crystalline cores within peroxisomes^90,91^. Notably, loss of CAT2 affects the activities of multiple peroxisomal enzymes in addition to catalase, including ACX, GOX, enoyl-CoA hydratase (ECH), L-hydroxyacyl-CoA dehydrogenase (HAD), KAT, isocitrate lyase (ICL), and malate synthase (MLS)^62,64,73,92^, although whether these effects are direct or indirect remains unclear.

In conclusion, CAIR1 is a previously undescribed cytosolic interactor of catalases that, likely in coordination with catalase maturation by NCA1, modulates catalase import dynamics into peroxisomes, thereby positively regulating catalase activity. The interaction with catalases is fine-tuned by reversible oligomerization of CAIR1, a process apparently governed by the cellular redox state. These findings suggest that CAIR1 functions as a ROS-responsive regulator, contributing to H_2_O_2_ homeostasis by modulating catalase activity under varying environmental conditions.

## Methods

### Plant material and growth conditions

*cair1-1* (GABI_438B07), *cat2-2* (SALK_076998)^69^, *nca1-1* (N68673)^41^, *uvr8-6* (SALK_033468)^59^, and *rup1 rup2*^58^ mutant lines as well as the Pro_35S_:GFP-PTS1^60^, sfGFP1-10^OPT^ targeted to peroxisomes (PX-sfGFP1-10; Pro_UBQ10_:sfGFP1-10^OPT^-SKL), cytoplasm (CYTO-sfGFP1-10; Pro_UBQ10_:sfGFP1-10^OPT^), nuclei (NU-sfGFP1-10; Pro_UBQ10_:sfGFP1-10^OPT^-NLS), and plasma membrane (PM-sfGFP1-10; Pro_UBQ10_:PIP2:sfGFP1-10^OPT^)^67^, and Pro_35S_:StrepII-3xHA-YFP transgenic lines^93^ are all in the Columbia (Col) background. *cair1-1 cat2-2* was generated by genetic crossing and genotyped by PCR (Supplementary Data 2).

As the T-DNA in *cair1-1* is in an intron and a second isoform of *CAIR1* is predicted in its second gene model (AT5G11580.2), we additionally generated two *cair1* null mutant alleles using CRISPR-Cas9. The guide oligonucleotides (Supplementary Data 2) were cloned into the binary vector pHEE401E binary vector following the published method^94^. The resulting constructs pHEE401E-CAIR1#1 and pHEE401E-CAIR1#2 were transformed into the wild type and *cair1-1* background, respectively. *cair1-2*/*at5g11580-2* contains a 278-bp deletion in the 5th exon in the *cair1-1* mutant background (thus knocking out both AT5G11580.1 and potential AT5G11580.2), and *cair1-3/at5g11580-3* contains an early frameshift mutation due to a single base pair insertion at position 92 after the start ATG (Supplementary Fig. 3).

For AP–MS experiments, plants were grown on soil under long-day (LD) conditions in a GroBank growth chamber (CLF Plant Climatics) (120 µmol m^−2^ s^−1^ white light, with or without 1.5 µmol m^−2^ s^−1^ UV-B)^95^. After three weeks, the plants exposed to UV-B were subjected to an additional 15 min of broadband UV-B irradiation before rosette tissue was harvested.

For experiments under aseptic conditions in vitro, Arabidopsis seeds were surface sterilized with chlorine gas and sown on half-strength Murashige & Skoog basal salt (MS) medium containing 0.5% or 0.3% (w/v) phytagel (Sigma) without sucrose. After 2 d of stratification at 4 °C, seedlings were grown under LD conditions in a standard growth chamber (MLR-350, Sanyo; 16-h/8-h light/dark cycle) with 66 µmol m^−2^ s^−1^ white light (measured with a LI-250 light meter; LI-COR Biosciences).

For the catalase lesion, seeds were sown on soil and stratified for 2 d at 4 °C before transfer to LD conditions (16-h/8-h light/dark cycle, 120 µmol m^−2^ s^−1^ white light) in a CLF GroBank growth chamber at ambient 420 ppm CO_2_ or in an Incrementum 1400 (Bronson Climate BV) supplemented with CO_2_ at 3000 ppm.

To induce oxidative stress through enhanced photorespiration, Petri dishes with plants grown on half-strength MS medium without sucrose for 7 d were sealed with three layers of Parafilm M (Amcor) to restrict the ambient air influx and exposed to continuous light (200 µmol m^−2^ s^−1^ white light) to prevent the confounding effects of night-time respiration on gas homeostasis.

### Plasmid construction and generation of transgenic plants

The coding sequences of *CAIR1*, *CAT1*, *CAT2*, *CAT3*, *NCA1*, and *HPR1* were amplified with corresponding primers respectively (Supplementary Data 2) from cDNA and cloned into entry vector pDONR207 using Gateway technology (ThermoFisher Scientific) to produce pDONR207-CAIR1-no stop codon, pDONR207-CAIR1, pDONR207-CAT1, pDONR207-CAT2, pDONR207-CAT3, pDONR207-NCA1, and pDONR207-HPR1. Cysteine mutant versions of CAIR1 were constructed using a two-step PCR-based site-directed mutagenesis protocol. First, the 5′-segment of CAIR1 cDNA was amplified with the CAIR1GWF primer and site-directed mutagenesis primers, which introduced Cysteine (C) to Alanine (A) amino acid exchanges. The PCR amplified and gel-purified 5′ cDNA segments were then used as 5′ primers with the CAIR1GWR primer to amplify the mutagenized full-length CAIR1 C-A cDNA versions, which were then cloned into pDONR207 with Gateway technology. This step was then repeated to produce pDONR207-CAIR1^12C-A^ and pDONR207-CAIR1^C356A;C545A^. To construct AD-CAIR1, BD-CAT1, BD-CAT2, BD-CAT3, and BD-NCA1, the entry vectors pDONR207-CAIR1, pDONR207-CAT1, pDONR207-CAT2, pDONR207-CAT3, and pDONR207-NCA1 were cloned with Gateway technology into pGADT7-GW-rfb^96^ and pGBKT7-GW^96^, respectively. To construct YFPN-CAT2, YFPC-HPR1, and YFPC-CAIR1, pDONR207-CAIR1, pDONR207-CAT2, and pDONR207-HPR1 were cloned into YFN43^97^ and YFC43^97^ with Gateway technology. To construct cLUC-CAT2, cLUC-NCA1, and CAIR1-nLUC, the coding sequence of CAT2, NCA1, and CAIR1 was obtained using corresponding primers, then cloned into pCAMBIA1300-cLUC^98^ and pCAMBIA 1300-nLUC^98^ with Gibson cloning, respectively. To construct Pro_35S_:CAIR1-YFP, Pro_35S_:YFP-CAIR1, Pro_35S_:YFP-CAIR1^12C-A^, Pro_35S_:YFP-CAIR1^C356A;C545A^, and Pro_35S_:CAIR1, pDONR207-CAIR1-no stop codon, pDONR207-CAIR1, pDONR207-CAIR1^12C-A^, and pDONR207-CAIR1^C356A;C545A^ were cloned into Pro_35S_:R1-R2-EYFP-Tnos (pGWB541), Pro_35S_:EYFP-R1-R2-Tnos (pGWB542), Pro_35S_:R1-R2-Tnos (pGWB402), and Pro_35S_:3xHA-R1-R2-Tnos (pGWB415)^99^, respectively, with Gateway technology.

To construct Pro_35S_:mCherry-PTS1(SKL), the mCherry-PTS1 fragment was amplified with primers mCherryF and mCherryPTS1R (Supplementary Data 2) from pBluescipt-C-term-mCherryStop-SpectR^56^ and cloned into entry vector pDONR207 using Gateway technology (ThermoFisher Scientific) to produce pDONR207-mCherry-PTS1. Then pDONR207-mCherry-PTS1 was cloned into Pro_35S_:R1-R2-Tnos (pGWB402)^99^ to produce Pro_35S_:mCherry-PTS1(SKL) with Gateway technology (ThermoFisher Scientific).

To generate Flag-CAT2, Flag-NCA1, GFP-CAIR1, and UVR8 constructs for expression in HEK293 cells, the coding sequences of *CAT2* and *NCA1* were cloned into the p3xFLAG-CMV-10 vector (Life Science Market). The coding sequence of *CAIR1* was cloned into the pEGFP-C1 vector (Axybio). The coding sequence of *UVR8*, including the stop codon, was cloned into the pEGFP-N1 vector (Axybio). All constructs were generated using Gibson assembly, and all primers used for cloning are listed in Supplementary Data 2.

To construct Pro_CAIR1_:CAIR1-GFP, three fragments were first amplified and cloned into entry vectors using MultiSite Gateway Recombination Cloning Technology (primers listed in Supplementary Data 2). A fragment containing the 5ʹ regulatory sequence of *CAIR1* and the *CAIR1* genomic sequence (2846 bp) was amplified and cloned into pDONR P4-P1R, the coding sequence of GFP, including stop codon, was amplified from N-SpR-GFP plasmid^56^ and cloned into pDONR221, and a fragment containing *CAIR1* 3ʹ regulatory sequence (2973 bp) was amplified and cloned into pDONR P2R-P3.

To construct Pro_NCA1_:NCA1-GFP, two fragments were first amplified and cloned into Gateway entry vectors (primers listed in Supplementary Data 2). A fragment containing the 5ʹ regulatory sequence and genomic sequence of NCA1 (3666 bp) was amplified and cloned into pDONR P4-P1R, and a fragment containing the NCA1 3ʹ regulatory sequence (1587 bp) was amplified and cloned into pDONR P2R-P3.

To construct Pro_CAT2_:GFP-CAT2, three fragments were first amplified and cloned into Gateway entry vectors (primers listed in Supplementary Data 2). A fragment containing the 5ʹ regulatory sequence of *CAT2* (943 bp) was amplified and cloned into pDONR P4-P1R, the coding sequence of GFP without a stop codon was amplified from N-SpR-GFP plasmid^56^ and cloned into pDONR221, and a fragment containing the *CAT2* genomic sequence, including 3ʹ regulatory sequence (3203 bp) was amplified and cloned into pDONR P2R-P3.

The entry vectors pDONR P4-P1R, pDONR221, and pDONR P2R-P3 carrying the above relevant fragments to generate Pro_CAIR1_:CAIR1-GFP, Pro_NCA1_:NCA1-GFP, and Pro_CAT2_:GFP-CAT2 genomic clones, respectively, were cloned into pFR7m34GW^100^ by MultiSite Gateway cloning technology.

To construct Pro_UVR8_:GFP-UVR8, an *E. coli* strain with BAC clone MGI19 carrying the *UVR8* gene was obtained from the Arabidopsis Biological Resource Center (ABRC). BAC recombineering^56^ was used to add a GFP tag at the N terminus of UVR8 within the *UVR8* genomic clone.

To construct CAIR1-sfGFP11, the coding sequence of *CAIR1* was amplified from cDNA with primers CAIR1F and CAIR1superfoldGFPR (Supplementary Data 2) and cloned into entry vector pDONR207 using Gateway technology (ThermoFisher Scientific) to produce pDONR207-CAIR1-sfGFP11. Then pDONR207-CAIR1-sfGFP11 was cloned into Pro_35S_:R1-R2-Tnos (pGWB402)^99^.

All resulting binary vectors were introduced into the agrobacterial strain GV3101 and transformed into the corresponding mutant background or wild type by the floral dip method^101^. Homozygous lines with single-locus insertions were identified and used in the T3 generation.

### Immunoblot analysis, co-immunoprecipitation

Rabbit polyclonal antibodies were generated against a synthetic peptide derived from the CAIR1 protein sequence (amino acids 373–387: C + RKISGGSSRFRDPVQ) and were affinity-purified against the peptide (Eurogentec).

For immunoblot analysis, total proteins were extracted by homogenizing samples in extraction buffer (50 mM Tris-HCl [pH 7.5], 10% [v/v] glycerol, 1 mM EDTA, 150 mM NaCl, 0.1% [v/v] Triton X-100) supplemented with freshly added 5 mM DTT, 1 mM PMSF, and 20 µM Sigma plant protease inhibitor cocktail. Protein concentrations were determined using the Bradford assay, following the manufacturer’s instructions. Aliquots of 20 µg protein were separated on 10% SDS-polyacrylamide gels and transferred to PVDF membranes using the iBlot 2 Dry Blotting System. GFP and YFP fusion proteins were detected using the Living Colors® GFP Monoclonal Antibody (Clontech, dilution 1:5000) in TBST buffer. UVR8 was detected using anti-UVR8^426–440^ (ref. ^59^) in TBST buffer. Endogenous catalase and HPR1 proteins were detected using anti-catalase (Agrisera, product no. AS09 501) and anti-HPR (Agrisera, product no. AS11 1797) antibodies in PBST and TBST buffers, respectively, following the manufacturer’s protocols. Horseradish peroxidase (HRP)–conjugated anti-rabbit and anti-mouse immunoglobulins (Dako A/S) were used as secondary antibodies. Protein bands were visualized using ECL Select Western Blotting Detection Reagent (GE Healthcare) and analyzed with an Amersham Imager 680 camera system (GE Healthcare). For loading control, the membrane was stripped with 0.2 M NaOH for 20 min and then probed with anti-actin antibody (Sigma).

For co-IP and affinity purification, protein extraction was performed as described above for immunoblot analysis. Total protein extracts were incubated with 40 µL GFP-Trap agarose (ChromoTek) affinity resin for 1 h at 4 °C, after ensuring equal protein concentrations. The GFP-Trap was then washed four times with washing buffer (50 mM Tris-HCl [pH 7.5], 150 mM NaCl). For co-immunoprecipitation, 40 µL of 1× Laemmli buffer was added to the samples, which were then boiled at 98 °C for 7 min. The samples were subsequently separated by SDS-PAGE for further analysis. Immunoblot analyses and co-IP assays were repeated three times with similar results.

### Affinity purification–mass spectrometry (AP–MS)

Approximately 15 g of freshly harvested leaf tissue per sample was ground in liquid nitrogen and extracted with 30 mL of lysis buffer (50 mM Tris-HCl [pH 7.5], 10% [v/v] glycerol, 1 mM EDTA, 150 mM NaCl, 1% [v/v] Triton X-100). The extracts were centrifuged at full speed (7200 × *g*) for 20 min at 4 °C. From each sample’s supernatant, technical triplicates of 20 mg of total protein each were generated and adjusted to an equal volume of 10 mL in lysis buffer and used for immunoprecipitation. The extracts were incubated for 1 h at 4 °C with 50 µL GFP-Trap® Agarose resin (ChromoTek), which had been prewashed three times with 1.5 mL extraction buffer and resuspended in 1 mL lysis buffer before addition to the protein samples. The resins were pelleted at 500 rpm at 4 °C and washed four times with 10 mL washing buffer (50 mM Tris–HCl [pH 7.5], 300 mM NaCl). Then, the resin was resuspended in 1 mL washing buffer, transferred to 1.5-mL Eppendorf tubes, and pelleted again. As controls, protein extracts were prepared in triplicate from wild type (Col) and Pro_35S_:StrepII-3xHA-YFP^93^ plants of the same age, harvested and processed in parallel with the Pro_UVR8_:GFP-UVR8, Pro_35S_:CAIR1-YFP, Pro_35S_:YFP-CAIR1, Pro_CAIR1_:CAIR1-GFP, and Pro_NCA1_:NCA1-GFP samples. For GFP-CAT2 purification, the co-purifying proteins obtained with the peroxisomal marker line Pro_35S_:GFP-PTS1^60^ were included as an additional control

Sample preparation and LC-MS/MS data acquisition: Enriched proteins were submitted to an on-bead digestion using trypsin. In brief, dry beads were re-dissolved in 25 µL digestion buffer 1 (50 mM Tris, pH 7.5, 2 M urea, 1 mM DTT, 5 ng/µL trypsin) and incubated for 30 min at 32 °C in a Thermomixer with 400 rpm. Next, beads were pelleted, and the supernatant was transferred to a fresh tube. Digestion buffer 2 (50 mM Tris, pH 7.5, 2 M urea, 5 mM CAA) was added to the beads, and after mixing, the beads were pelleted, and the collected supernatant was combined with the previous one. The combined supernatants were then incubated o/n at 32 °C in a Thermomixer set to 400 rpm, with samples protected from light during incubation. The digestion was stopped by adding 1 µL TFA and desalted with C18 Empore disk membranes according to the StageTip protocol^102^.

Dried peptides were re-dissolved in 2% (v/v) ACN, 0.1% (v/v) TFA (10 µL) for analysis. Samples were analyzed using an EASY-nLC 1200 (Thermo Fisher) coupled to a Q Exactive Plus mass spectrometer (Thermo Fisher) or using an EASY-nLC 1000 (Thermo Fisher) coupled to a Q Exactive mass spectrometer (Thermo Fisher). Peptides were separated on 16-cm frit-less silica emitters (New Objective, 75-µm inner diameter), packed in-house with reversed-phase ReproSil-Pur C18 AQ 1.9 µm resin (Dr. Maisch, High Performance LC GmbH). Peptides were loaded on the column and eluted for 115 min using a segmented linear gradient of 5% to 95% solvent B (0 min: 5% B; 0–5 min: 5% B; 5–65 min: 20% B; 65–90 min: 35% B; 90–100 min: 55% B; 100–105 min: 95% B; 105–115 min: 95% B) (solvent A 0% ACN, 0.1% [v/v] FA; solvent B 80% [v/v] ACN, 0.1% [v/v] FA) at a flow rate of 300 nL/min. Mass spectra were acquired in data-dependent acquisition mode with a TOP15 method. MS spectra were acquired in the Orbitrap analyzer with a mass range of 300–1750 m/z at a resolution of 70,000 FWHM and a target value of 3 × 10^6 ^ions. Precursors were selected with an isolation window of 1.3 m/z (Q Exactive Plus) or 2.0 m/z (Q Exactive). HCD fragmentation was performed at a normalized collision energy of 25. MS/MS spectra were acquired with a target value of 10^5^ ions at a resolution of 17,500 FWHM, a maximum injection time (max.) of 55 ms, and a fixed first mass of m/z 100. Peptides with a charge of +1, greater than 6, or with unassigned charge state were excluded from fragmentation for MS2, dynamic exclusion for 30 s prevented repeated selection of precursors. Alternatively, samples were analyzed using an Ultimate 3000 RSLC nano (Thermo Fisher) coupled to an Orbitrap Exploris 480 mass spectrometer equipped with a FAIMS Pro interface for Field asymmetric ion mobility separation (Thermo Fisher). Peptides were pre-concentrated on an Acclaim PepMap 100 pre-column (75 µM × 2 cm, C18, 3 µM, 100 Å, Thermo Fisher) using the loading pump and buffer A** (water, 0.1% [v/v] TFA) with a flow of 7 µL/min for 5 min. Peptides were separated on 16-cm frit-less silica emitters (New Objective, 75-µm inner diameter), packed in-house with reversed-phase ReproSil-Pur C18 AQ 1.9 µm resin (Dr. Maisch, High Performance LC GmbH). Peptides were loaded on the column and eluted for 130 min using a segmented linear gradient of 5% to 95% solvent B (0 min: 5% B; 0–5 min: 5% B; 5–65 min: 20% B; 65–90 min: 35% B; 90–100 min: 55% B; 100–105 min: 95% B; 105–115 min: 95% B; 115–115.1 min: 5% B; 115.1–130 min: 5% B) (solvent A 0% ACN, 0.1% [v/v] FA; solvent B 80% [v/v] ACN, 0.1% [v/v] FA) at a flow rate of 300 nL/min. Mass spectra were acquired in data-dependent acquisition mode with a TOP_S method using a cycle time of 2 s. For field asymmetric ion mobility separation (FAIMS), two compensation voltages (−45 and −60) were applied, and the cycle time was set to 1 s for each experiment. MS spectra were acquired in the Orbitrap analyzer with a mass range of 320–1200 m/z at a resolution of 60,000 FWHM and a normalized AGC target of 300%. Precursors were filtered using the MIPS option (MIPS mode = peptide), and the intensity threshold was set to 5000. Precursors were selected with an isolation window of 1.6 m/z. HCD fragmentation was performed at a normalized collision energy of 30%. MS/MS spectra were acquired with a target value of 75% ions at a resolution of 15,000 FWHM, an inject time set to auto, and a fixed first mass of m/z 120. Peptides with a charge of +1, greater than 6, or with unassigned charge state were excluded from fragmentation for MS2.

Data analysis: Raw data were processed using MaxQuant software (version 1.6.3.4, http://www.maxquant.org/)^103^ with label-free quantification (LFQ) and iBAQ enabled^104^. MS/MS spectra were searched by the Andromeda search engine against a combined database containing the sequences from Arabidopsis (TAIR10_pep_20101214; ftp://ftp.arabidopsis.org/home/tair/Proteins/TAIR10_protein_lists/) and sequences of 248 common contaminant proteins and decoy sequences. Trypsin specificity was required, and a maximum of two missed cleavages was allowed. Minimal peptide length was set to seven amino acids. Carbamidomethylation of cysteine residues was set as fixed, oxidation of methionine, and protein N-terminal acetylation as variable modifications. Peptide-spectrum-matches and proteins were retained if they were below a false discovery rate of 1%. Statistical analysis of the MaxLFQ values was carried out using Perseus (version 1.5.8.5, http://www.maxquant.org/). Quantified proteins were filtered for reverse hits and hits “identified by site”, MaxLFQ values were log2 transformed. After grouping samples by condition, only those proteins were retained for the subsequent analysis that had two valid values in one of the conditions. Two-sample *t*-tests were performed using a permutation-based FDR of 5%. Alternatively, quantified proteins were grouped by condition, and only those hits were retained that had 3 valid values in one of the conditions. Missing values were imputed from a normal distribution (1.8 downshift, separately for each column). The Perseus output was exported and further processed using Excel. Candidates with LFQ intensity value > 20 and log2 ratio > 2 compared to both WT and “YFP” AP-controls were retained (Supplementary Data 1).

The MS data have been deposited in the ProteomeXchange Consortium via the PRIDE partner repository with the dataset identifier PXD073328.

### RNA extraction and reverse transcription quantitative PCR (RT-qPCR)

Total RNA from plant tissues was isolated using the Plant RNeasy kit (Qiagen), including DNase treatment, following the manufacturer’s protocol. cDNA synthesis was carried out using the Taqman Reverse Transcription Reagent kit (Thermo Fisher Scientific). Each RT-qPCR reaction contained cDNA equivalent to 3 ng of RNA, synthesized with a 1:1 mixture of oligo(dT) primers and random hexamers. RT-qPCR was performed using PowerUp SYBR Green Master Mix (Thermo Fisher Scientific) on a QuantStudio™ 5 Real-Time PCR System, and Ct values were calculated using QuantStudio Design and Analysis Software v1.4.3 (Thermo Fisher Scientific). Gene expression levels were calculated using the ΔΔCT method^105^, with *PP2AA3* (*PROTEIN PHOSPHATASE 2A SUBUNIT A3*, AT1G13320) or *UBQ5* (*UBIQUITIN 5*, AT3G62250) as reference genes^106^. Each experiment included three independent biological replicates.

### Transient protein expression, BiFC, and LCI assay in *Nicotiana benthamiana*

To transiently express proteins in *N. benthamiana*, constructs were introduced into Agrobacterium strain GV3101, and the OD values were monitored using a spectrophotometer, after which cultures were co-infiltrated alongside silencing inhibitor P19^107^ into young leaves of *N. benthamiana*. Plants were grown under LD conditions (16-h light/8-h dark) for 2 d. For the BiFC assay, the signal was visualized and documented using a confocal microscope (Leica SP8). For the LCI assay, luciferin (1 mM) was sprayed evenly onto infiltrated leaves and kept in darkness for 7 min, after which LUC activity was monitored with LB985 NightShade with indiGo (v2.485) software (Berthold Tech).

### Visualization of domain structure and structure modeling

The domain structures of RCC1 family proteins were visualized using TBtools-II (v2.485)^108^. The CAIR1 structure was modeled using AlphaFold 3^109^. Three-dimensional structural models were visualized using PyMOL (v2.5.2) software.

### Phylogenetic analysis

To identify RCC1-like proteins, BLAST searches were performed in Phytozome (v13) using the Arabidopsis CAIR1 protein sequence as a query against the following selected genomes: *Arabidopsis thaliana* (Araport11), *Glycine max* (Wm82.a6.v1), *Populus trichocarpa* (v4.1), *Sorghum bicolor* (v5.1), *Oryza sativa* (v7.0), *Zea mays* (RefGen_V4), *Brachypodium distachyon* (v2.1), *Hordeum vulgare* (Morex V3), *Thuja plicata* (v3.1), *Marchantia polymorpha* (v3.1), *Ceratopteris richardii* (v2.1), *Selaginella moellendorffii* (v1.0), *Chlamydomonas reinhardtii* (v5.6), and *Physcomitrium patens* (v7.1). Additionally, RCC1-like protein sequences for *Ginkgo biloba* L. were retrieved from the Ginkgo Genome Database (version 2019). After compiling the retrieved RCC1-like protein sequences, we retained only the primary isoform for each gene and excluded sequences containing a C-terminal FYVE zinc finger domain from downstream analysis. Protein sequences were aligned in MAFFT v7^110^ using the automatic strategy selection mode (*--auto*). Maximum Likelihood phylogenetic reconstruction was next performed with IQ-TREE 2^111^ using the best-fitting substitution model selected by ModelFinder. Node support was evaluated using 1,000 ultrafast bootstrap replicates and 1000 SH-aLRT replicates. The resulting phylogenetic tree was visualized and modified using iTOL v7 (https://itol.embl.de/).

### Catalase activity measurements

Catalase activity was measured as previously established^112,113^. Eighty milligrams of 5-d-old seedlings was homogenized in 1.5 mL extraction buffer (100 mM PBS, pH 7.0) for 5 min at 4 °C. The homogenate was centrifuged at 12,000 × *g* for 15 min at 4 °C, and the supernatant was collected. Hundred microliters of crude protein extract was diluted to a total volume of 3 mL reaction mixture in 100 mM PBS (pH 7.0) containing 30 mM H_2_O_2_. Catalase activity was measured spectrophotometrically at room temperature by monitoring the decrease in absorbance at 240 nm resulting from the decomposition of H_2_O_2_. Protein concentrations were determined using the Bio-Rad Protein Assay Dye Reagent Concentrate, following the manufacturer’s instructions.

### Fv/Fm measurement

The maximum quantum efficiency of PSII (*F*v/*F*m) was assayed by measuring plant chlorophyll fluorescence using a PSI (Photon Systems Instruments) FluorCam 800 MF and the FluorCam7 (version 1.2.5.3) software. Before measurements, plates were incubated in darkness for 7 min which was confirmed to be sufficient to reach steady state under the plant growth conditions used^114^. Maximum fluorescence in the dark (*F*m) was measured using saturating pulses of 2000 µmol m^−2^ s^−1^ (white light, 400–720 nm) for 960 ms, followed by orange-red light (620 nm) detection pulses (10 µs). *F*v/*F*m was calculated as *F*v/*F*m = (*F*m − *F*o)/*F*m, where *F*m is the maximal fluorescence, and *F*o is the minimal fluorescence in the dark-adapted state^115^.

### Hypocotyl length measurements

Seedlings were grown for 5 d under white light (3.6 µmol m^−2^ s^−1^) supplemented with UV-B (1.5 µmol m^−2^ s^−1^) (+), or not (−)^116^, after which approximately 30 seedlings per genotype or condition were aligned on an agar plate and scanned. The length of individual hypocotyls was quantified using ImageJ software (1.50i).

### Extraction and quantification of anthocyanins

Seedlings were grown for 4 d in white light (3.6 µmol m^−2^ s^−1^) supplemented with UV-B (1.5 µmol m^−2^ s^−1^) (+), or not (−)^116^, and approximately 20 mg of seedlings were collected for each genotype and condition. Samples were frozen in liquid nitrogen, ground to a fine powder, and extracted with 400 µL of extraction buffer (99% [v/v] methanol, 1% [v/v] HCl). Extraction was carried out for at least 1 h by incubating the samples on a rotary shaker at 4 °C. Following centrifugation for 5 min, the absorbance of 200 µL of the clarified supernatant was measured at 530 nm and 655 nm with a TECAN Spark 10 M Multimode Microplate Reader. Relative anthocyanin content was calculated using the formula: (A530–0.25*A655)/seedling mass (mg).

### Extraction of phenolic compounds and measurements of absorption spectra

Phenolic compounds were extracted from 50 mg of fresh plant material homogenized with a Silamat S6 (Ivodar Vivadent) in 400 μL of 80% (v/v) methanol^117^. The homogenate was incubated for 10 min at 70 °C with shaking at 800 × *g*, followed by centrifugation at 12,000 × *g* for 10 min at room temperature (RT). Subsequently, 200 µL of the clear supernatant was transferred to a transparent 96-well plate (UV-SATR MICROPLATE, Greiner), and absorbance spectra were recorded using a TECAN Spark 10 M Multimode Microplate Reader.

### Yeast growth assay

The AD and BD constructs were co-transformed into the yeast strain Y2Hgold (Takara) and selected on SD/–Leu/–Trp double-dropout (DDO) plates. To assess the protein interactions, the transformed yeasts were placed on SD/–Leu/–Trp/–His triple-dropout (TDO) plates. The interactions were observed after 4 d at 30 °C.

### Confocal laser-scanning microscopy

The localization of YFP- or GFP-tagged proteins was imaged using a Leica TCS SP8 confocal microscope (Leica, Bensheim, Germany). GFP and YFP were excited with an Argon laser at 488 nm, and the emitted fluorescence was detected between 493 and 550 nm.

### Statistical analysis

All data were analyzed and graphed with GraphPad Prism software (version 11.0.0).

### Reporting summary

Further information on research design is available in the Nature Portfolio Reporting Summary linked to this article.

## Supplementary information

Supplementary Information

Description of Additional Supplementary Files

Supplementary Data 1

Supplementary Data 2

Supplementary Data 3

Reporting Summary

Transparent Peer Review file

## Source data

Source Data File

## Data Availability

The data supporting the findings in this study are available and described within the paper and its supplementary files. The MS data have been deposited in the ProteomeXchange Consortium via the PRIDE partner repository with the dataset identifier PXD073328. Source data are provided with this paper.

## References

[1] Mittler, R., Zandalinas, S. I., Fichman, Y. & Van Breusegem, F. Reactive oxygen species signalling in plant stress responses. *Nat. Rev. Mol. Cell Biol.* **23**, 663–679 (2022). 35760900 10.1038/s41580-022-00499-2

[2] Apel, K. & Hirt, H. Reactive oxygen species: metabolism, oxidative stress, and signal transduction. *Annu. Rev. Plant Biol.* **55**, 373–399 (2004). 15377225 10.1146/annurev.arplant.55.031903.141701

[3] Mittler, R. ROS are good. *Trends Plant Sci.* **22**, 11–19 (2017). 27666517 10.1016/j.tplants.2016.08.002

[4] Chi, Y. H. et al. Redox-dependent functional switching of plant proteins accompanying with their structural changes. *Front. Plant Sci.* **4**, 277 (2013). 10.3389/fpls.2013.00277 PMC3724125 23898340

[5] Mhamdi, A. et al. Catalase function in plants: a focus on Arabidopsis mutants as stress-mimic models. *J. Exp. Bot.* **61**, 4197–4220 (2010). 20876333 10.1093/jxb/erq282

[6] Mhamdi, A., Noctor, G. & Baker, A. Plant catalases: peroxisomal redox guardians. *Arch. Biochem. Biophys.* **525**, 181–194 (2012). 22546508 10.1016/j.abb.2012.04.015

[7] Pan, R., Liu, J., Wang, S. & Hu, J. Peroxisomes: versatile organelles with diverse roles in plants. *N. Phytol.* **225**, 1410–1427 (2020). 10.1111/nph.16134 31442305

[8] Hu, J. et al. Plant peroxisomes: biogenesis and function. *Plant Cell* **24**, 2279–2303 (2012). 22669882 10.1105/tpc.112.096586 PMC3406917

[9] Del Rio, L. A. & Lopez-Huertas, E. ROS generation in peroxisomes and its role in cell signaling. *Plant Cell Physiol.* **57**, 1364–1376 (2016). 27081099 10.1093/pcp/pcw076

[10] Kao, Y. T., Gonzalez, K. L. & Bartel, B. Peroxisome function, biogenesis, and dynamics in plants. *Plant Physiol.* **176**, 162–177 (2018). 29021223 10.1104/pp.17.01050 PMC5761812

[11] Molina-Moya, E., Rodriguez-Gonzalez, A., Pelaez-Vico, M. A., Sandalio, L. M. & Romero-Puertas, M. C. Peroxisomal-dependent signalling and dynamics modulate plant stress responses: reactive oxygen and nitrogen species as key molecules. *J. Exp. Bot.* **76**, 3706–3721 (2025). 39989015 10.1093/jxb/eraf072 PMC12404776

[12] Foyer, C. H., Bloom, A. J., Queval, G. & Noctor, G. Photorespiratory metabolism: genes, mutants, energetics, and redox signaling. *Annu. Rev. Plant Biol.* **60**, 455–484 (2009). 19575589 10.1146/annurev.arplant.043008.091948

[13] Theodoulou, F. L. & Eastmond, P. J. Seed storage oil catabolism: a story of give and take. *Curr. Opin. Plant Biol.* **15**, 322–328 (2012). 22516438 10.1016/j.pbi.2012.03.017

[14] Tuzet, A., Rahantaniaina, M. S. & Noctor, G. Analyzing the function of catalase and the ascorbate-glutathione pathway in H_2_O_2_ processing: insights from an experimentally constrained kinetic model. *Antioxid. Redox Signal.* **30**, 1238–1268 (2019). 30044135 10.1089/ars.2018.7601

[15] Baker, A. et al. Catalase: a critical node in the regulation of cell fate. *Free Radic. Biol. Med.* **199**, 56–66 (2023). 36775107 10.1016/j.freeradbiomed.2023.02.009

[16] Scandalios, J. G., Tong, W.-F. & Roupakias, D. G. Cat3, a third gene locus coding for a tissue-specific catalase in maize: genetics, intracellular location, and some biochemical properties. *Mol. Gen. Genet.* **179**, 33–41 (1980).

[17] Reumann, S., Ma, C., Lemke, S. & Babujee, L. AraPerox. A database of putative Arabidopsis proteins from plant peroxisomes. *Plant Physiol.* **136**, 2587–2608 (2004). 15333753 10.1104/pp.104.043695 PMC523325

[18] Kato, N., Nelson, G. & Lauersen, K. J. Subcellular localizations of catalase and exogenously added fatty acid in *Chlamydomonas reinhardtii*. *Cells* **10**, 1940 (2021). 34440712 10.3390/cells10081940 PMC8391285

[19] Kamigaki, A. et al. Identification of peroxisomal targeting signal of pumpkin catalase and the binding analysis with PTS1 receptor. *Plant J.* **33**, 161–175 (2003). 12943550 10.1046/j.0960-7412.2003.001605.x

[20] Cross, L. L., Ebeed, H. T. & Baker, A. Peroxisome biogenesis, protein targeting mechanisms and PEX gene functions in plants. *Biochim. Biophys. Acta* **1863**, 850–862 (2016). 26408938 10.1016/j.bbamcr.2015.09.027

[21] Gould, S. J., Keller, G. A., Hosken, N., Wilkinson, J. & Subramani, S. A conserved tripeptide sorts proteins to peroxisomes. *J. Cell Biol.* **108**, 1657–1664 (1989). 2654139 10.1083/jcb.108.5.1657 PMC2115556

[22] Kato, A., Hayashi, M., Kondo, M. & Nishimura, M. Targeting and processing of a chimeric protein with the N-terminal presequence of the precursor to glyoxysomal citrate synthase. *Plant Cell* **8**, 1601–1611 (1996). 8837511 10.1105/tpc.8.9.1601 PMC161301

[23] Nito, K., Hayashi, M. & Nishimura, M. Direct interaction and determination of binding domains among peroxisomal import factors in *Arabidopsis thaliana*. *Plant Cell Physiol.* **43**, 355–366 (2002). 11978862 10.1093/pcp/pcf057

[24] Glover, J. R., Andrews, D. W. & Rachubinski, R. A. *Saccharomyces cerevisiae* peroxisomal thiolase is imported as a dimer. *Proc. Natl. Acad. Sci. USA* **91**, 10541–10545 (1994). 7937990 10.1073/pnas.91.22.10541 PMC45057

[25] Lee, M. S., Mullen, R. T. & Trelease, R. N. Oilseed isocitrate lyases lacking their essential type 1 peroxisomal targeting signal are piggybacked to glyoxysomes. *Plant Cell* **9**, 185–197 (1997). 9061950 10.1105/tpc.9.2.185 PMC156910

[26] He, A., Dean, J. M. & Lodhi, I. J. Peroxisomes as cellular adaptors to metabolic and environmental stress. *Trends Cell Biol.* **31**, 656–670 (2021). 33674166 10.1016/j.tcb.2021.02.005 PMC8566112

[27] Lin, C. C., Foyer, C. H., Wright, M. & Baker, A. Redox regulation of LSD1/CATALASE 2 phase separation condensates controls location and functions. *N. Phytol.* **247**, 2824–2838 (2025). 10.1111/nph.70374 PMC12371180 40686090

[28] Al-Hajaya, Y., Karpinska, B., Foyer, C. H. & Baker, A. Nuclear and peroxisomal targeting of catalase. *Plant Cell Environ.* **45**, 1096–1108 (2022). 35040158 10.1111/pce.14262 PMC9305541

[29] Dubreuil, M. M. et al. Systematic identification of regulators of oxidative stress reveals non-canonical roles for peroxisomal import and the pentose phosphate pathway. *Cell Rep.* **30**, 1417–1433 e1417 (2020). 32023459 10.1016/j.celrep.2020.01.013 PMC7184925

[30] Okumoto, K. et al. The peroxisome counteracts oxidative stresses by suppressing catalase import via Pex14 phosphorylation. *Elife* **9**, e55896 (2020). 10.7554/eLife.55896 PMC7498260 32831175

[31] Frugoli, J. A. et al. Catalase is encoded by a multigene family in *Arabidopsis thaliana* (L.) Heynh. *Plant Physiol.* **112**, 327–336 (1996). 8819328 10.1104/pp.112.1.327 PMC157953

[32] Su, T. et al. The Arabidopsis catalase triple mutant reveals important roles of catalases and peroxisome-derived signaling in plant development. *J. Integr. Plant Biol.* **60**, 591–607 (2018). 29575603 10.1111/jipb.12649

[33] Yang, Z., Mhamdi, A. & Noctor, G. Analysis of catalase mutants underscores the essential role of CATALASE2 for plant growth and day length-dependent oxidative signalling. *Plant Cell Environ.* **42**, 688–700 (2019). 30291629 10.1111/pce.13453

[34] Oshima, Y. et al. Plant catalase is imported into peroxisomes by Pex5p but is distinct from typical PTS1 import. *Plant Cell Physiol.* **49**, 671–677 (2008). 18308759 10.1093/pcp/pcn038

[35] Fujikawa, Y. et al. Effect of mutation of C-terminal and heme binding region of *Arabidopsis* catalase on the import to peroxisomes. *Biosci. Biotechnol. Biochem.* **83**, 322–325 (2019). 30295129 10.1080/09168451.2018.1530094

[36] Li, Y., Chen, L., Mu, J. & Zuo, J. LESION SIMULATING DISEASE1 interacts with catalases to regulate hypersensitive cell death in Arabidopsis. *Plant Physiol.* **163**, 1059–1070 (2013). 23958864 10.1104/pp.113.225805 PMC3793025

[37] Lv, T. et al. The calmodulin-binding protein IQM1 interacts with CATALASE2 to affect pathogen defense. *Plant Physiol.* **181**, 1314–1327 (2019). 31548265 10.1104/pp.19.01060 PMC6836832

[38] Kneeshaw, S. et al. Nucleoredoxin guards against oxidative stress by protecting antioxidant enzymes. *Proc. Natl. Acad. Sci. USA*. **114**, 8414–8419 (2017). 28724723 10.1073/pnas.1703344114 PMC5547615

[39] Yang, T. & Poovaiah, B. W. Hydrogen peroxide homeostasis: activation of plant catalase by calcium/calmodulin. *Proc. Natl. Acad. Sci. USA* **99**, 4097–4102 (2002). 11891305 10.1073/pnas.052564899 PMC122654

[40] Liu, J. et al. Two NCA1 isoforms interact with catalase in a mutually exclusive manner to redundantly regulate its activity in rice. *BMC Plant Biol.* **19**, 105 (2019). 30885124 10.1186/s12870-019-1707-0 PMC6421683

[41] Hackenberg, T. et al. Catalase and NO CATALASE ACTIVITY1 promote autophagy-dependent cell death in Arabidopsis. *Plant Cell* **25**, 4616–4626 (2013). 24285797 10.1105/tpc.113.117192 PMC3875739

[42] Li, J. et al. A chaperone function of NO CATALASE ACTIVITY1 is required to maintain catalase activity and for multiple stress responses in Arabidopsis. *Plant Cell* **27**, 908–925 (2015). 25700484 10.1105/tpc.114.135095 PMC4558663

[43] Hadjebi, O., Casas-Terradellas, E., Garcia-Gonzalo, F. R. & Rosa, J. L. The RCC1 superfamily: from genes, to function, to disease. *Biochim. Biophys. Acta* **1783**, 1467–1479 (2008). 18442486 10.1016/j.bbamcr.2008.03.015

[44] Tilbrook, K. et al. The UVR8 UV-B photoreceptor: perception, signaling and response. *Arabidopsis Book* **11**, e0164 (2013). 23864838 10.1199/tab.0164 PMC3711356

[45] Su, C. et al. RUG3 and ATM synergistically regulate the alternative splicing of mitochondrial *nad2* and the DNA damage response in *Arabidopsis thaliana*. *Sci. Rep.* **7**, 43897 (2017). 28262819 10.1038/srep43897 PMC5338318

[46] Kühn, K. et al. The RCC1 family protein RUG3 is required for splicing of *nad2* and complex I biogenesis in mitochondria of *Arabidopsis thaliana*. *Plant J.* **67**, 1067–1080 (2011). 21623974 10.1111/j.1365-313X.2011.04658.x

[47] Duarte, G. T. et al. Plasticity of rosette size in response to nitrogen availability is controlled by an RCC1-family protein. *Plant Cell Environ.* **44**, 3398–3411 (2021). 34228823 10.1111/pce.14146

[48] Ji, H. et al. The RCC1 family protein SAB1 negatively regulates ABI5 through multidimensional mechanisms during postgermination in Arabidopsis. *N. Phytol.* **222**, 907–922 (2019). 10.1111/nph.15653 30570158

[49] Ji, H. et al. The Arabidopsis RCC1 family protein TCF1 regulates freezing tolerance and cold acclimation through modulating lignin biosynthesis. *PLoS Genet.* **11**, e1005471 (2015). 26393916 10.1371/journal.pgen.1005471 PMC4579128

[50] Furutani, M. et al. Polar recruitment of RLD by LAZY1-like protein during gravity signaling in root branch angle control. *Nat. Commun.* **11**, 76 (2020). 31900388 10.1038/s41467-019-13729-7 PMC6941992

[51] Rizzini, L. et al. Perception of UV-B by the *Arabidopsis* UVR8 protein. *Science* **332**, 103–106 (2011). 21454788 10.1126/science.1200660

[52] Podolec, R., Demarsy, E. & Ulm, R. Perception and signaling of ultraviolet-B radiation in plants. *Annu. Rev. Plant Biol.* **72**, 793–822 (2021). 33636992 10.1146/annurev-arplant-050718-095946

[53] Kliebenstein, D. J., Lim, J. E., Landry, L. G. & Last, R. L. *Arabidopsis* UVR8 regulates ultraviolet-B signal transduction and tolerance and contains sequence similarity to human regulator of chromatin condensation 1. *Plant Physiol.* **130**, 234–243 (2002). 12226503 10.1104/pp.005041 PMC166556

[54] Brown, B. A. et al. A UV-B-specific signaling component orchestrates plant UV protection. *Proc. Natl. Acad. Sci. USA*. **102**, 18225–18230 (2005). 16330762 10.1073/pnas.0507187102 PMC1312397

[55] Binkert, M. et al. Revisiting chromatin binding of the Arabidopsis UV-B photoreceptor UVR8. *BMC Plant Biol.* **16**, 42 (2016). 26864020 10.1186/s12870-016-0732-5 PMC4750278

[56] Hu, Z. et al. Gene modification by fast-track recombineering for cellular localization and isolation of components of plant protein complexes. *Plant J.* **100**, 411–429 (2019). 31276249 10.1111/tpj.14450 PMC6852550

[57] Heijde, M. et al. Constitutively active UVR8 photoreceptor variant in Arabidopsis. *Proc. Natl. Acad. Sci. USA*. **110**, 20326–20331 (2013). 24277841 10.1073/pnas.1314336110 PMC3864333

[58] Gruber, H. et al. Negative feedback regulation of UV-B-induced photomorphogenesis and stress acclimation in Arabidopsis. *Proc. Natl. Acad. Sci. USA* **107**, 20132–20137 (2010). 21041653 10.1073/pnas.0914532107 PMC2993346

[59] Favory, J. J. et al. Interaction of COP1 and UVR8 regulates UV-B-induced photomorphogenesis and stress acclimation in Arabidopsis. *EMBO J.* **28**, 591–601 (2009). 19165148 10.1038/emboj.2009.4 PMC2657586

[60] Mano, S. et al. Distribution and characterization of peroxisomes in Arabidopsis by visualization with GFP: dynamic morphology and actin-dependent movement. *Plant Cell Physiol.* **43**, 331–341 (2002). 11917088 10.1093/pcp/pcf037

[61] Williams, C., Bener Aksam, E., Gunkel, K., Veenhuis, M. & Van Der Klei, I. J. The relevance of the non-canonical PTS1 of peroxisomal catalase. *Biochim. Biophys. Acta* **1823**, 1133–1141 (2012). 22546606 10.1016/j.bbamcr.2012.04.006

[62] Yuan, H.-M., Liu, W.-C. & Lu, Y.-T. CATALASE2 coordinates SA-mediated repression of both auxin accumulation and JA biosynthesis in plant defenses. *Cell Host Microbe* **21**, 143–155 (2017). 28182949 10.1016/j.chom.2017.01.007

[63] Zhang, Z. et al. Association-dissociation of glycolate oxidase with catalase in rice: a potential switch to modulate intracellular H_2_O_2_ levels. *Mol. Plant* **9**, 737–748 (2016). 26900141 10.1016/j.molp.2016.02.002

[64] Yang, T. et al. Consecutive oxidative stress in CATALASE2-deficient Arabidopsis negatively regulates Glycolate Oxidase1 activity through S-nitrosylation. *Physiol. Plant* **177**, e70040 (2025). 39777728 10.1111/ppl.70040

[65] Chen, L. et al. Transnitrosylation mediated by the non-canonical catalase ROG1 regulates nitric oxide signaling in plants. *Dev. Cell* **53**, 444–457 e445 (2020). 32330424 10.1016/j.devcel.2020.03.020

[66] Zhang, Q. et al. S-nitrosylation may inhibit the activity of COP1 in plant photomorphogenesis. *Biochem. Biophys. Res. Commun.* **719**, 150096 (2024). 38749091 10.1016/j.bbrc.2024.150096

[67] Park, E., Lee, H.-Y., Woo, J., Choi, D. & Dinesh-Kumar, S. P. Spatiotemporal monitoring of *Pseudomonas syringae* effectors via type III secretion using split fluorescent protein fragments. *Plant Cell* **29**, 1571–1584 (2017). 28619883 10.1105/tpc.17.00047 PMC5559745

[68] Kerppola, T. K. Visualization of molecular interactions by fluorescence complementation. *Nat. Rev. Mol. Cell Biol.* **7**, 449–456 (2006). 16625152 10.1038/nrm1929 PMC2512262

[69] Queval, G. et al. Conditional oxidative stress responses in the *Arabidopsis* photorespiratory mutant *cat2* demonstrate that redox state is a key modulator of daylength-dependent gene expression, and define photoperiod as a crucial factor in the regulation of H_2_O_2_-induced cell death. *Plant J.* **52**, 640–657 (2007). 17877712 10.1111/j.1365-313X.2007.03263.x

[70] Kaurilind, E., Xu, E. & Brosche, M. A genetic framework for H_2_O_2_ induced cell death in *Arabidopsis thaliana*. *BMC Genom.* **16**, 837 (2015). 10.1186/s12864-015-1964-8 PMC4619244 26493993

[71] Chaouch, S. et al. Peroxisomal hydrogen peroxide is coupled to biotic defense responses by ISOCHORISMATE SYNTHASE1 in a daylength-related manner. *Plant Physiol.* **153**, 1692–1705 (2010). 20543092 10.1104/pp.110.153957 PMC2923881

[72] Tognetti, V. B. et al. Perturbation of indole-3-butyric acid homeostasis by the UDP-glucosyltransferase UGT74E2 modulates Arabidopsis architecture and water stress tolerance. *Plant Cell* **22**, 2660–2679 (2010). 20798329 10.1105/tpc.109.071316 PMC2947170

[73] Liu, W. C. et al. CATALASE2 functions for seedling postgerminative growth by scavenging H_2_O_2_ and stimulating ACX2/3 activity in Arabidopsis. *Plant Cell Environ.* **40**, 2720–2728 (2017). 28722222 10.1111/pce.13031

[74] Juul, T. et al. The in vivo toxicity of hydroxyurea depends on its direct target catalase. *J. Biol. Chem.* **285**, 21411–21415 (2010). 20452979 10.1074/jbc.M110.103564 PMC2898382

[75] Cremers, C. M. & Jakob, U. Oxidant sensing by reversible disulfide bond formation. *J. Biol. Chem.* **288**, 26489–26496 (2013). 23861395 10.1074/jbc.R113.462929 PMC3772196

[76] Roy, D. et al. Redox-regulated Aux/IAA multimerization modulates auxin responses. *Science* **389**, eadu1470 (2025). 40504951 10.1126/science.adu1470

[77] Buskiewicz, I. A. et al. Reactive oxygen species induce virus-independent MAVS oligomerization in systemic lupus erythematosus. *Sci. Signal.* **9**, ra115 (2016). 27899525 10.1126/scisignal.aaf1933 PMC5321043

[78] Huang, M. et al. Reactive oxygen species regulate nucleostemin oligomerization and protein degradation. *J. Biol. Chem.* **286**, 11035–11046 (2011). 21242306 10.1074/jbc.M110.208470 PMC3064158

[79] Apanasets, O. et al. PEX5, the shuttling import receptor for peroxisomal matrix proteins, is a redox-sensitive protein. *Traffic* **15**, 94–103 (2014). 24118911 10.1111/tra.12129

[80] Walton, P. A., Brees, C., Lismont, C., Apanasets, O. & Fransen, M. The peroxisomal import receptor PEX5 functions as a stress sensor, retaining catalase in the cytosol in times of oxidative stress. *Biochim. Biophys. Acta Mol. Cell Res.* **1864**, 1833–1843 (2017). 28760655 10.1016/j.bbamcr.2017.07.013

[81] Mhamdi A., Van Breusegem F. Reactive oxygen species in plant development. *Development* **145**, dev164376 (2018). 10.1242/dev.164376 30093413

[82] Wei, X. et al. H_2_O_2_ negatively regulates aluminum resistance via oxidation and degradation of the transcription factor STOP1. *Plant Cell* **36**, 688–708 (2024). 37936326 10.1093/plcell/koad281 PMC10896299

[83] Tian, Y. et al. Hydrogen peroxide positively regulates brassinosteroid signaling through oxidation of the BRASSINAZOLE-RESISTANT1 transcription factor. *Nat. Commun.* **9**, 1063 (2018). 29540799 10.1038/s41467-018-03463-x PMC5852159

[84] Garcia-Santamarina, S., Boronat, S. & Hidalgo, E. Reversible cysteine oxidation in hydrogen peroxide sensing and signal transduction. *Biochemistry* **53**, 2560–2580 (2014). 24738931 10.1021/bi401700f

[85] Shaikhali, J. et al. The redox-sensitive transcription factor Rap2.4a controls nuclear expression of 2-Cys peroxiredoxin A and other chloroplast antioxidant enzymes. *BMC Plant Biol.* **8**, 48 (2008). 18439303 10.1186/1471-2229-8-48 PMC2386467

[86] Shaikhali, J. et al. Redox-mediated mechanisms regulate DNA binding activity of the G-group of basic region leucine zipper (bZIP) transcription factors in Arabidopsis. *J. Biol. Chem.* **287**, 27510–27525 (2012). 22718771 10.1074/jbc.M112.361394 PMC3431687

[87] Lee, E. S. et al. Redox-dependent structural switch and CBF activation confer freezing tolerance in plants. *Nat. Plants* **7**, 914–922 (2021). 34155371 10.1038/s41477-021-00944-8

[88] He, H., Van Breusegem, F. & Mhamdi, A. Redox-dependent control of nuclear transcription in plants. *J. Exp. Bot.* **69**, 3359–3372 (2018). 29659979 10.1093/jxb/ery130

[89] Foyer, C. H. et al. On the move: redox-dependent protein relocation in plants. *J. Exp. Bot.* **71**, 620–631 (2020). 31421053 10.1093/jxb/erz330

[90] Lodhi, I. J. & Semenkovich, C. F. Peroxisomes: a nexus for lipid metabolism and cellular signaling. *Cell Metab.* **19**, 380–392 (2014). 24508507 10.1016/j.cmet.2014.01.002 PMC3951609

[91] Rhodin, J. Electron microscopy of the kidney. *Am. J. Med.* **24**, 661–675 (1958). 13520766 10.1016/0002-9343(58)90373-5

[92] Eastmond, P. J. MONODEHYROASCORBATE REDUCTASE4 is required for seed storage oil hydrolysis and postgerminative growth in Arabidopsis. *Plant Cell* **19**, 1376–1387 (2007). 17449810 10.1105/tpc.106.043992 PMC1913749

[93] Lapin, D. et al. A coevolved EDS1-SAG101-NRG1 module mediates cell death signaling by TIR-domain immune receptors. *Plant Cell* **31**, 2430–2455 (2019). 31311833 10.1105/tpc.19.00118 PMC6790079

[94] Wang, Z. P. et al. Egg cell-specific promoter-controlled CRISPR/Cas9 efficiently generates homozygous mutants for multiple target genes in Arabidopsis in a single generation. *Genome Biol.* **16**, 144 (2015). 26193878 10.1186/s13059-015-0715-0 PMC4507317

[95] Arongaus, A. B. et al. Arabidopsis RUP2 represses UVR8-mediated flowering in noninductive photoperiods. *Genes Dev.* **32**, 1332–1343 (2018). 30254107 10.1101/gad.318592.118 PMC6169840

[96] Lu, Q. et al. Arabidopsis homolog of the yeast TREX-2 mRNA export complex: components and anchoring nucleoporin. *Plant J.* **61**, 259–270 (2010). 19843313 10.1111/j.1365-313X.2009.04048.x

[97] Belda-Palazon, B. et al. Aminopropyltransferases involved in polyamine biosynthesis localize preferentially in the nucleus of plant cells. *PLoS One* **7**, e46907 (2012). 23056524 10.1371/journal.pone.0046907 PMC3466176

[98] Chen, H. et al. Firefly luciferase complementation imaging assay for protein-protein interactions in plants. *Plant Physiol.* **146**, 368–376 (2008). 18065554 10.1104/pp.107.111740 PMC2245818

[99] Nakagawa, T. et al. Improved Gateway binary vectors: high-performance vectors for creation of fusion constructs in transgenic analysis of plants. *Biosci. Biotechnol. Biochem.* **71**, 2095–2100 (2007). 17690442 10.1271/bbb.70216

[100] Kalmbach, L. et al. Putative pectate lyase PLL12 and callose deposition through polar CALS7 are necessary for long-distance phloem transport in Arabidopsis. *Curr. Biol.* **33**, 926–939 e929 (2023). 36805125 10.1016/j.cub.2023.01.038

[101] Clough, S. J. & Bent, A. F. Floral dip: a simplified method for Agrobacterium-mediated transformation of *Arabidopsis thaliana*. *Plant J.* **16**, 735–743 (1998). 10069079 10.1046/j.1365-313x.1998.00343.x

[102] Rappsilber, J., Ishihama, Y. & Mann, M. Stop and go extraction tips for matrix-assisted laser desorption/ionization, nanoelectrospray, and LC/MS sample pretreatment in proteomics. *Anal. Chem.* **75**, 663–670 (2003). 12585499 10.1021/ac026117i

[103] Cox, J. & Mann, M. MaxQuant enables high peptide identification rates, individualized p.p.b.-range mass accuracies and proteome-wide protein quantification. *Nat. Biotechnol.* **26**, 1367–1372 (2008). 19029910 10.1038/nbt.1511

[104] Tyanova, S., Temu, T. & Cox, J. The MaxQuant computational platform for mass spectrometry-based shotgun proteomics. *Nat. Protoc.* **11**, 2301–2319 (2016). 27809316 10.1038/nprot.2016.136

[105] Livak, K. J. & Schmittgen, T. D. Analysis of relative gene expression data using real-time quantitative PCR and the 2−ΔΔCT method. *Methods* **25**, 402–408 (2001). 11846609 10.1006/meth.2001.1262

[106] Czechowski, T., Stitt, M., Altmann, T., Udvardi, M. K. & Scheible, W. R. Genome-wide identification and testing of superior reference genes for transcript normalization in Arabidopsis. *Plant Physiol.* **139**, 5–17 (2005). 16166256 10.1104/pp.105.063743 PMC1203353

[107] Lombardi, R. et al. High-level HIV-1 Nef transient expression in *Nicotiana benthamiana* using the P19 gene silencing suppressor protein of Artichoke Mottled Crinckle Virus. *BMC Biotechnol.* **9**, 96 (2009). 19930574 10.1186/1472-6750-9-96 PMC2785776

[108] Chen, C. et al. TBtools-II: A “one for all, all for one” bioinformatics platform for biological big-data mining. *Mol. Plant* **16**, 1733–1742 (2023). 37740491 10.1016/j.molp.2023.09.010

[109] Abramson, J. et al. Accurate structure prediction of biomolecular interactions with AlphaFold 3. *Nature* **630**, 493–500 (2024). 38718835 10.1038/s41586-024-07487-w PMC11168924

[110] Katoh, K. & Standley, D. M. MAFFT multiple sequence alignment software version 7: improvements in performance and usability. *Mol. Biol. Evol.* **30**, 772–780 (2013). 23329690 10.1093/molbev/mst010 PMC3603318

[111] Minh, B. Q. et al. IQ-TREE 2: new models and efficient methods for phylogenetic inference in the genomic era. *Mol. Biol. Evol.* **37**, 1530–1534 (2020). 32011700 10.1093/molbev/msaa015 PMC7182206

[112] Aebi H. Catalase in vitro. In: *Methods in Enzymology* (Academic Press, 1984). 10.1016/s0076-6879(84)05016-3 6727660

[113] Noctor, G., Mhamdi, A. & Foyer, C. H. Oxidative stress and antioxidative systems: recipes for successful data collection and interpretation. *Plant Cell Environ.* **39**, 1140–1160 (2016). 26864619 10.1111/pce.12726

[114] Murchie, E. H. & Lawson, T. Chlorophyll fluorescence analysis: a guide to good practice and understanding some new applications. *J. Exp. Bot.* **64**, 3983–3998 (2013). 23913954 10.1093/jxb/ert208

[115] Baker, N. R. Chlorophyll fluorescence: a probe of photosynthesis in vivo. *Annu Rev. Plant Biol.* **59**, 89–113 (2008). 18444897 10.1146/annurev.arplant.59.032607.092759

[116] Oravecz, A. et al. CONSTITUTIVELY PHOTOMORPHOGENIC1 is required for the UV-B response in Arabidopsis. *Plant Cell* **18**, 1975–1990 (2006). 16829591 10.1105/tpc.105.040097 PMC1533968

[117] Leonardelli, M. et al. Photoreceptor-induced sinapate synthesis contributes to photoprotection in Arabidopsis. *Plant Physiol.* **196**, 1518–1533 (2024). 38918833 10.1093/plphys/kiae352 PMC11444301

