# Supplementary material for: A redox-regulated RCC1-like protein controls catalase activity in Arabidopsis
Source: Nat Commun. 2026 Aug 15;17:9807. doi: 10.1038/s41467-026-76792-x (PMC13575196; doi:10.1038/s41467-026-76792-x)
Supplement: Supplementary file 1 [file 41467_2026_76792_MOESM1_ESM.pdf]

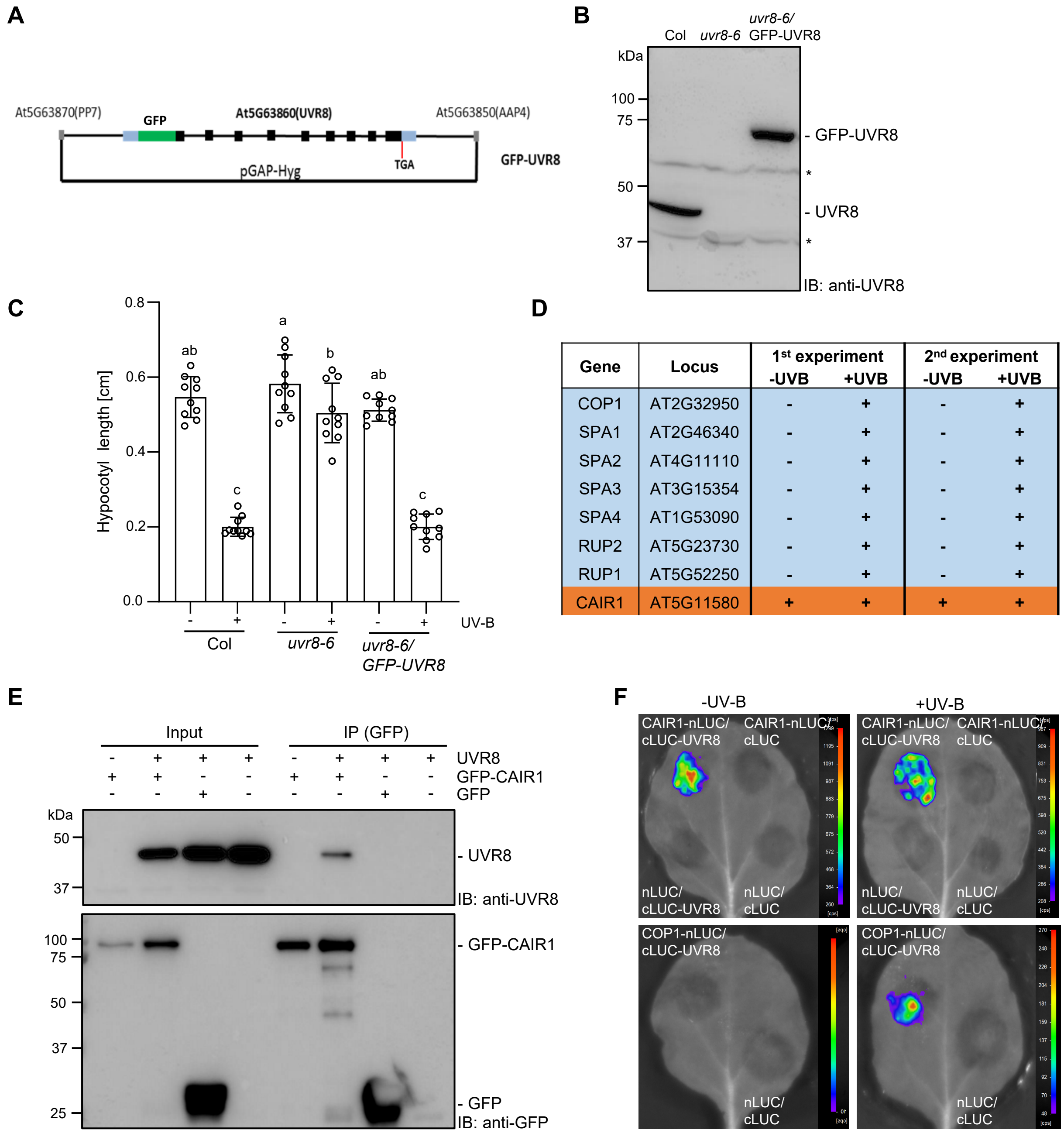

Gene	Locus	1st experiment -UVB	1st experiment +UVB	2nd experiment -UVB	2nd experiment +UVB
COP1	AT2G32950	-	+	-	+
SPA1	AT2G46340	-	+	-	+
SPA2	AT4G11110	-	+	-	+
SPA3	AT3G15354	-	+	-	+
SPA4	AT1G53090	-	+	-	+
RUP2	AT5G23730	-	+	-	+
RUP1	AT5G52250	-	+	-	+
CAIR1	AT5G11580	+	+	+	+

Supplementary Fig. 1. UVR8 interacts with a protein of unknown function (AT5G11580).
(A) Schematic representation of the Pro$_{UVR8}$:GFP-UVR8 genomic construct. Black boxes: exons of *UVR8*; black lines: introns, intergenic regions to neighboring genes At5g63870 (*PP7; SERINE/THREONINE PHOSPHATASE 7*) and At5G63850 (*AAP4¸ AMINO ACID PERMEASE 4*), as well as pGAP-Hyg vector backbone; green box: GFP coding sequence; blue boxes: 5′ and 3′ UTRs of *UVR8*. Translational start ATG provided by inserted GFP sequence; *UVR8* TGA stop codon is indicated. **(B)** Immunoblot analysis of UVR8 and GFP-UVR8 levels in wild-type (Col), *uvr8-6*, and *uvr8-6*/Pro$_{UVR8}$:GFP-UVR8 line #16 (*uvr8-6*/GFP-UVR8) seedlings. Asterisks (*) indicate cross-reacting unspecific bands. **(C)** Quantification of hypocotyl length of Col, *uvr8-6* and *uvr8-6*/GFP-UVR8 seedlings grown in white light (3.6 µmol m^{-2} s^{-1}) supplemented (+) or not (-) with UV-B (1.5 µmol m^{-2} s^{-1}). Data points and means ± SD are shown (n = 10 seedlings). Shared letters indicate no significant differences among means ($P > 0.05$; two-way ANOVA, Tukey's test). **(D)** Selected proteins identified in two independent AP–MS experiments of GFP-UVR8 complexes from plants grown in white light in the absence (-UV-B) or presence (+UV-B) of supplementary UV-B; "+" and "-" signs indicate, respectively, enrichment or not in the AP–MS analysis. Known UVR8 interactors and complex constituents are highlighted with a light blue background and the previously undescribed CAIR1 (AT5G11580) in orange. **(E)** Co-IP assay of endogenous UVR8 using GFP-trap purification, showing co-IP of UVR8 with GFP-CAIR1 but not GFP when co-expressed in HEK293 cells. IB, immunoblotting; IP, immunoprecipitation. **(F)** Transient expression in *N. benthamiana* leaves for LCI assay of the interaction of cLUC-UVR8 with co-expressed CAIR1-nLUC and COP1-nLUC (positive control in presence of UV-B), as well as the respective empty vector negative controls (nLUC and cLUC). Before imaging luciferase activity, the leaves were exposed to UV-B for 10 min (+UV-B), or not (-UV-B).

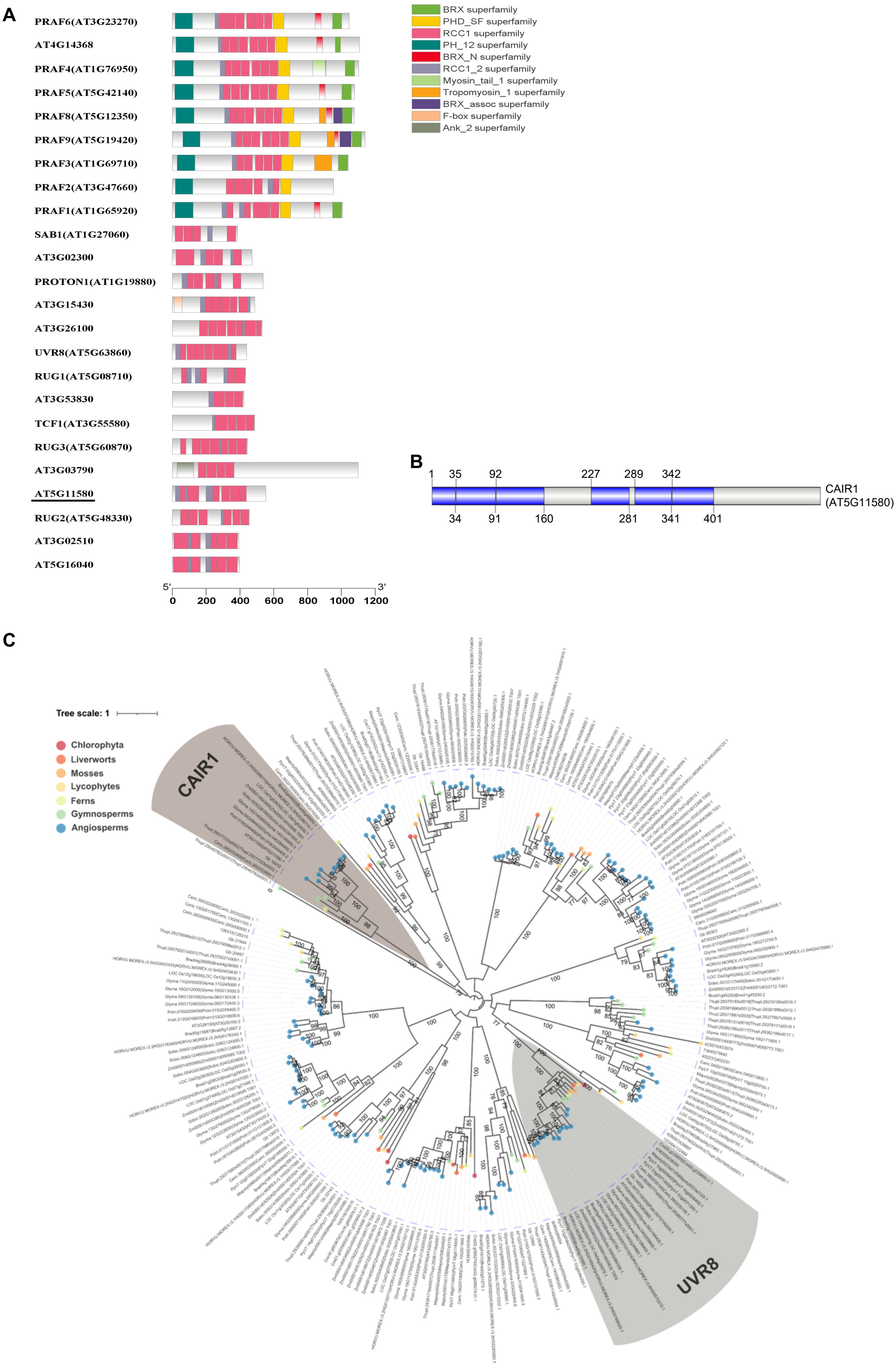

Supplementary Fig. 2. CAIR1 (AT5G11580) is a previously uncharacterized RCC1-like family member.

Supplementary Fig. 2. CAIR1 (AT5G11580) is a previously uncharacterized RCC1-like family member.
(A) Domain structures of Arabidopsis RCC1-like family members. The sequences of 24 RCC1 family proteins were downloaded from TAIR and the domain architectures were retrieved from the Pfam protein families database (https://www.ncbi.nlm.nih.gov/Structure/bwrpsb/bwrpsb.cgi). Phylogenetic tree and structures were visualized schematically using TBtools-II (https://github.com/CJ-Chen/TBtools-II/releases). **(B)** CAIR1 features six RCC1 domains and an extended C-terminal region. The 553 amino acid–long CAIR1 sequence was obtained from TAIR. The domain was subsequently analyzed using the EBI database (https://www.ebi.ac.uk), and the results were visualized using IBS 2.0 (https://ibs.renlab.org/#/server). First (top) and last (bottom) amino acid residue for each RCC1 domain is given. **(C)** Maximum-likelihood phylogenetic tree of the RCC1 family across Viridiplantae, excluding the nine PRAF proteins (see panel A). The tree was rooted arbitrarily for graphical display purposes. Both CAIR1 and UVR8 form well-supported monophyletic clades in the phylogenetic tree, with maximal bootstrap support (BS = 100%). AT: *Arabidopsis thaliana*, Glyma: *Glycine max*, Potri: *Populus trichocarpa*, Sobic: *Sorghum bicolor*, Os: *Oryza sativa*, Zm: *Zea mays*, Bradi: *Brachypodium distachyon*, HORVU: *Hordeum vulgare*, Gb: *Ginkgo biloba*, Thupl: *Thuja plicata*, Mapoly: *Marchantia polymorpha*, Pp: *Physcomitrium patens,* Ceric: *Ceratopteris richardii*, numbers only: *Selaginella moellendorffii*, and Cre: *Chlamydomonas reinhardtii*.

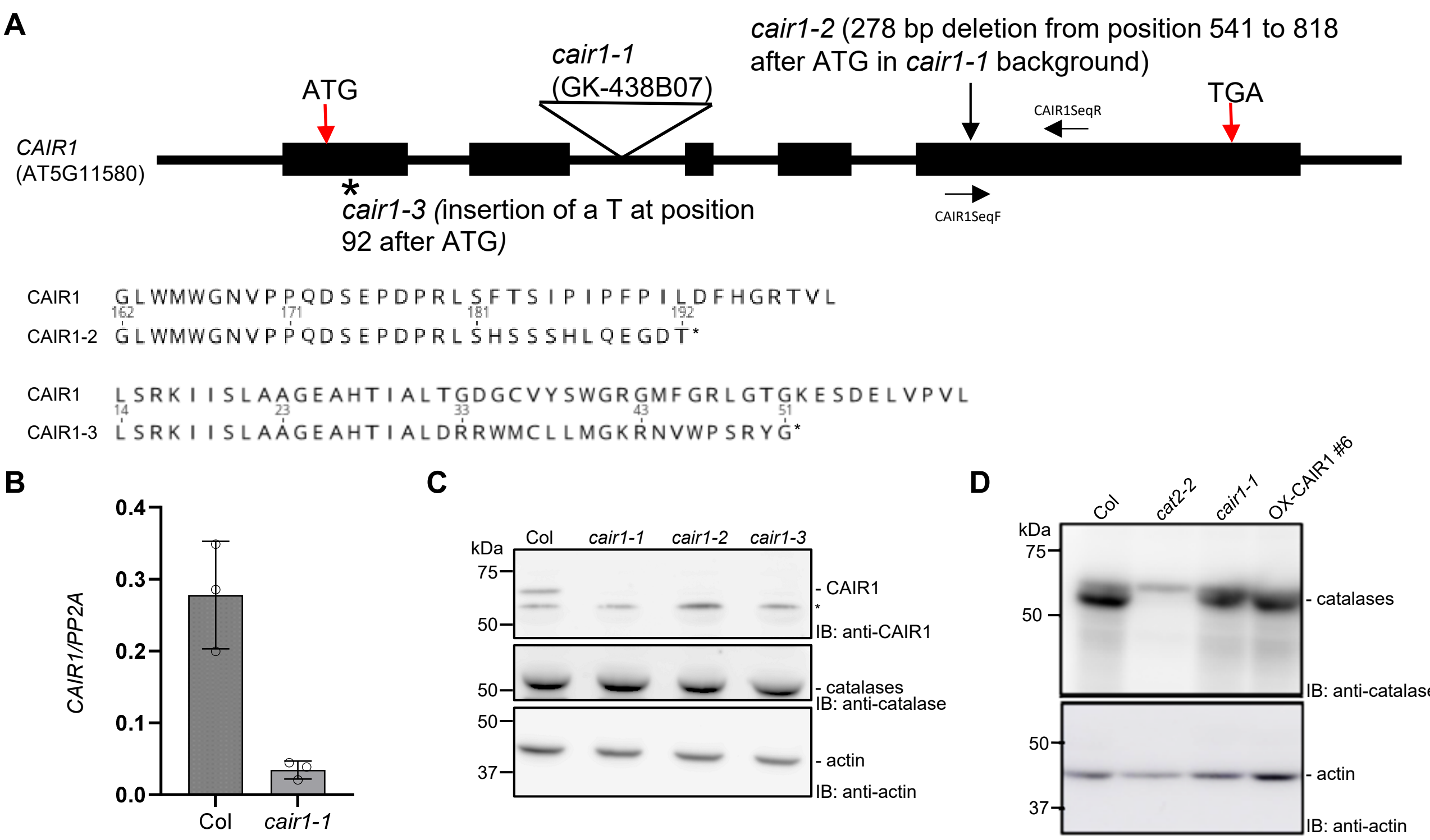

Supplementary Fig. 3. *cair1* mutant alleles.

(A) Schematic structure of the *CAIR1* gene and mutant alleles. Black boxes indicate exons, black lines introns, as well as 5′ and 3′ UTRs. Translational start ATG and stop TGA codons are indicated with red arrows. Amino acid sequences and premature stop codons (*) for CRISPR/Cas9-generated *cair1-2* and *cair1-3* are indicated below adjacent to wild-type CAIR1 sequences. Approximate location of primers CAIR1SeqF and CAIR1SeqR used for RT-qPCR are indicated. **(B)** RT-qPCR analysis of *CAIR1* expression in wild type (Col) and *cair1-1* using primers CAIR1SeqF and CAIR1SeqR. Individual data points and means ± SD are shown (n = 3 biological replicates). **(C)** Immunoblot analysis of CAIR1 and catalase levels in 5-d-old *cair1-1*, *cair1-2*, and *cair1-3* seedlings compared to that in Col. *, unspecific band. **(D)** Immunoblot analysis of catalase levels in 6-d-old *cat2-2*, *cair1-1*, and *cair1-1*/Pro$_{35S}$:CAIR1 line #6 (OX-CAIR1 #6) seedlings compared to that in Col. (C, D) Actin levels are shown as loading control.

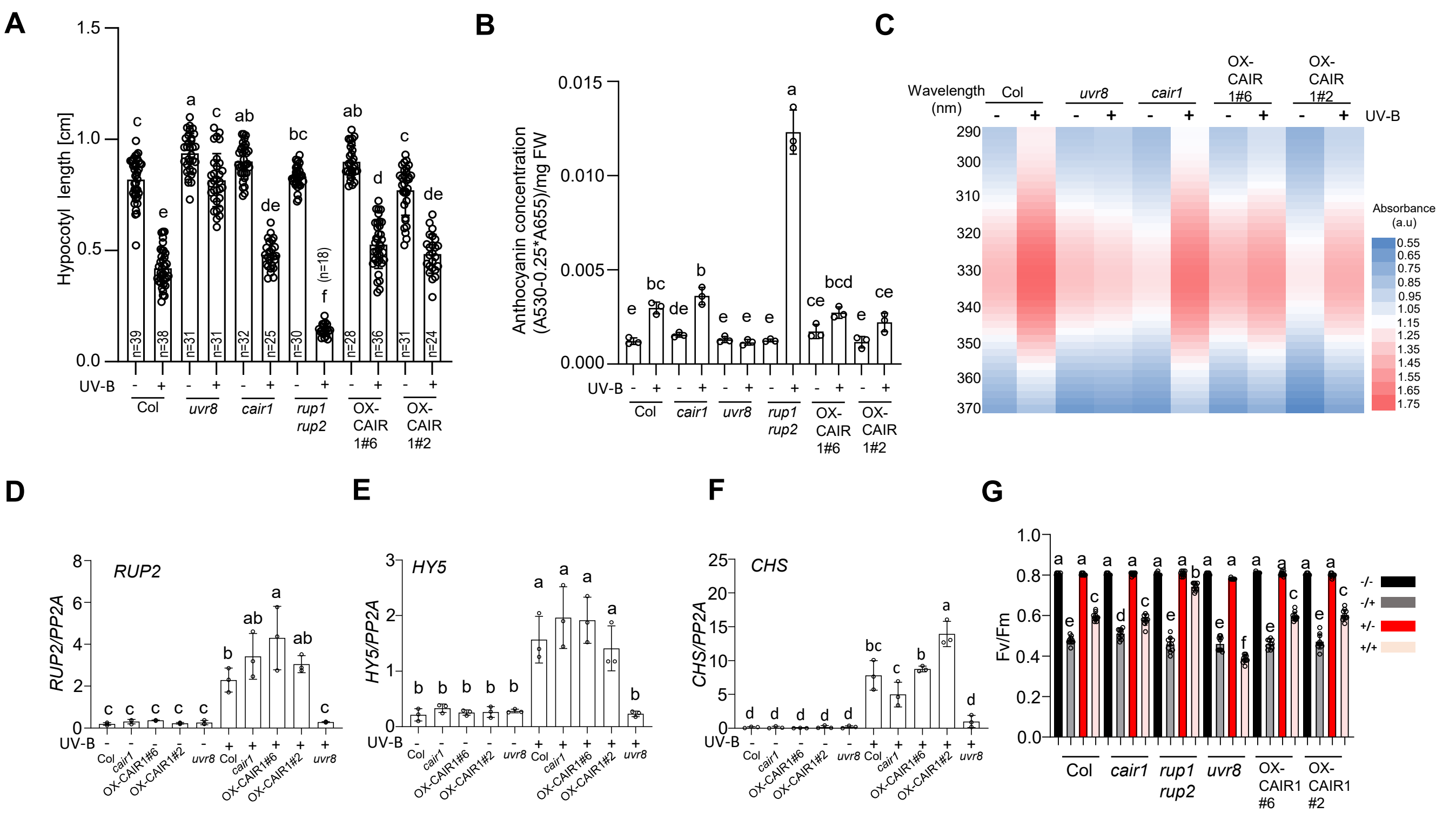

Supplementary Fig. 4. CAIR1 is not required for UV-B-induced photomorphogenic responses and UV-B acclimation.

(A–C) Quantification of hypocotyl length (A), anthocyanin concentration (B), and absorbance spectra (280–400 nm) of methanolic extracts (C; UV-absorbing metabolites) of 5-d-old wild-type (Col), *uvr8-6* (*uvr8*), *rup1 rup2*, *cair1-1* (*cair1*), and two CAIR1 overexpression lines (OX-CAIR1 lines #2 and #6) seedlings grown in white light (3.6 µmol m^{-2} s^{-1}) supplemented with UV-B (1.5 µmol m^{-2} s^{-1}) (+), or not (−). (A) Individual data points and means ± SD are shown (n ≥ 18 seedlings). (B) Individual data points and means ± SD are shown (n = 3 biological replicates). **(D–F)** RT-qPCR analysis of *RUP2*, *HY5*, and *CHS* expression levels after UV-B treatment. Five-d-old Col, *uvr8*, *rup1 rup2*, *cair1*, and OX-CAIR1 lines #2 and #6 seedlings were grown in white light (3.6 µmol m^{-2} s^{-1}), then exposed (+) or not (-) to 3 h of narrowband UV-B (1.5 µmol m^{-2} s^{-1}). Individual data points and means ± SD are shown (n = 3 biological replicates). **(G)** *Fv*/*Fm* measurements of Col, *uvr8*, *rup1 rup2*, *cair1*, and OX-CAIR1 lines #2 and #6 seedlings. Seven-d-old light-grown seedlings were exposed (acclimated, +/−) or not (−/−) to UV-B for 3 d with supplementary narrowband UV-B (0.08 mW cm^{-2}). After 10 d, acclimated and non-acclimated plants were exposed to 2 h of broadband UV stress irradiation (non-acclimated and stressed −/+; acclimated and stressed +/+; 2.2 mW cm^{-2}). Data points and means ± SD are shown (n = 10 individual plants). (A–G) Shared letters indicate no significant differences among means (P > 0.05; two-way ANOVA, Tukey's test).

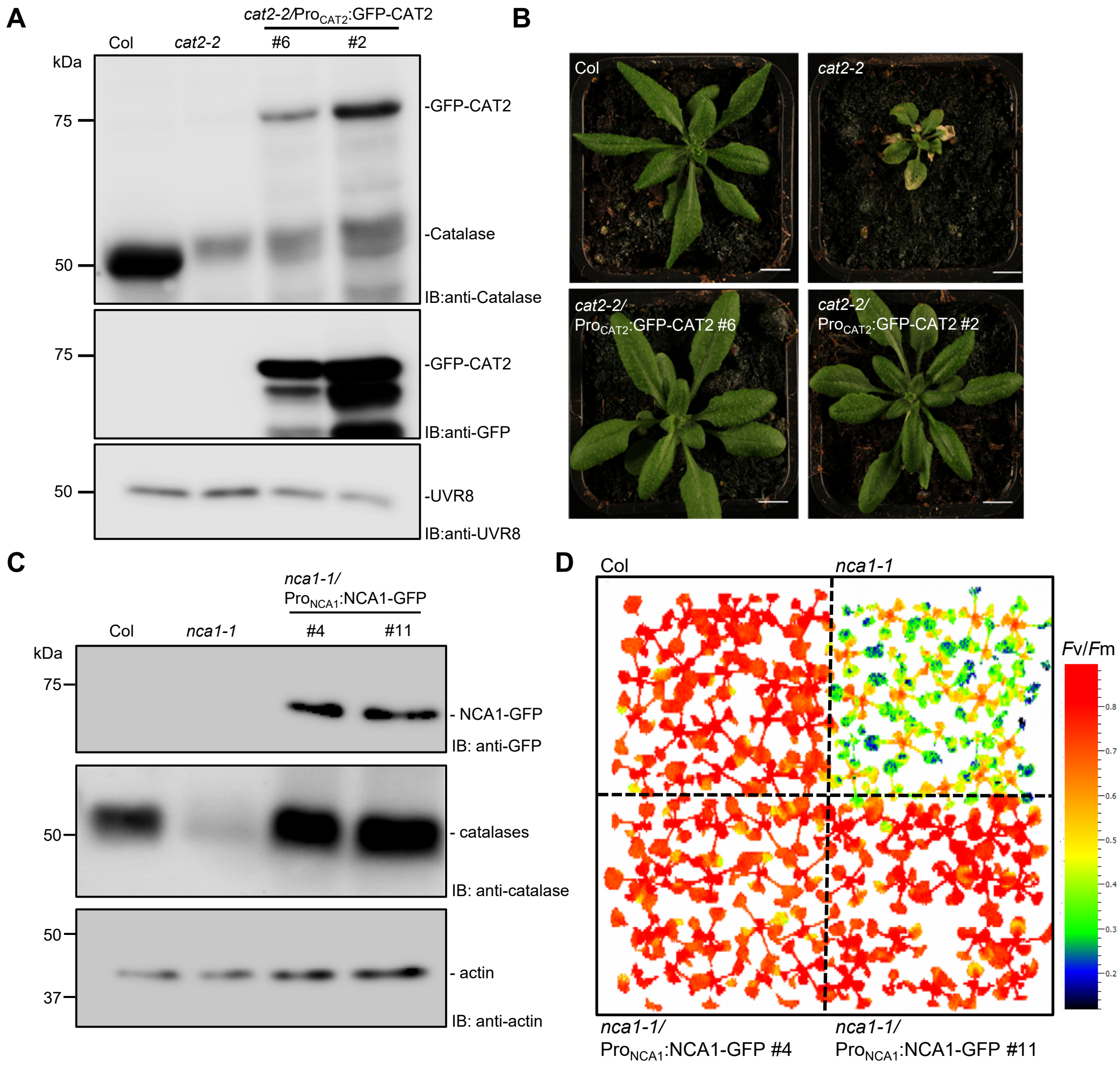

Supplementary Fig. 5. Functional complementation of *cat2-2* and *nca1-1* mutant phenotypes by Pro_{CAT2}:GFP-CAT2 and Pro_{NCA1}:NCA1-GFP genomic clones, respectively.

(A) Immunoblot analysis of 3-week-old *cat2-2*/Pro_{CAT2}:GFP-CAT2 complementation lines #6 and #4 compared to wild type (Col) and *cat2-2* using anti-catalase and anti-GFP antibodies. IB, immunoblotting. *, degradation products; **, unspecific cross-reacting band. **(B)** The transgenic lines expressing GFP-CAT2 fully rescue the small size and leaf lesion phenotypes of *cat2-2* under long-day (LD) conditions. **(C, D)** The transgenic lines expressing NCA1-GFP fully restore catalase protein stability and alleviate the oxidative stress sensitivity of *nca1-1*. (C) Immunoblot analysis of catalase and NCA1-GFP levels in 7-d-old *nca1-1*/Pro_{NCA1}:GFP-CAT2 complementation lines #4 and #11 compared to Col and *nca1-1*. Actin levels are shown as loading control. IB, immunoblotting. (D) *Fv*/*Fm* measurements of 7-d-old seedlings of the indicated genotypes grown for 2 d under gas limitation (enhanced photorespiration).

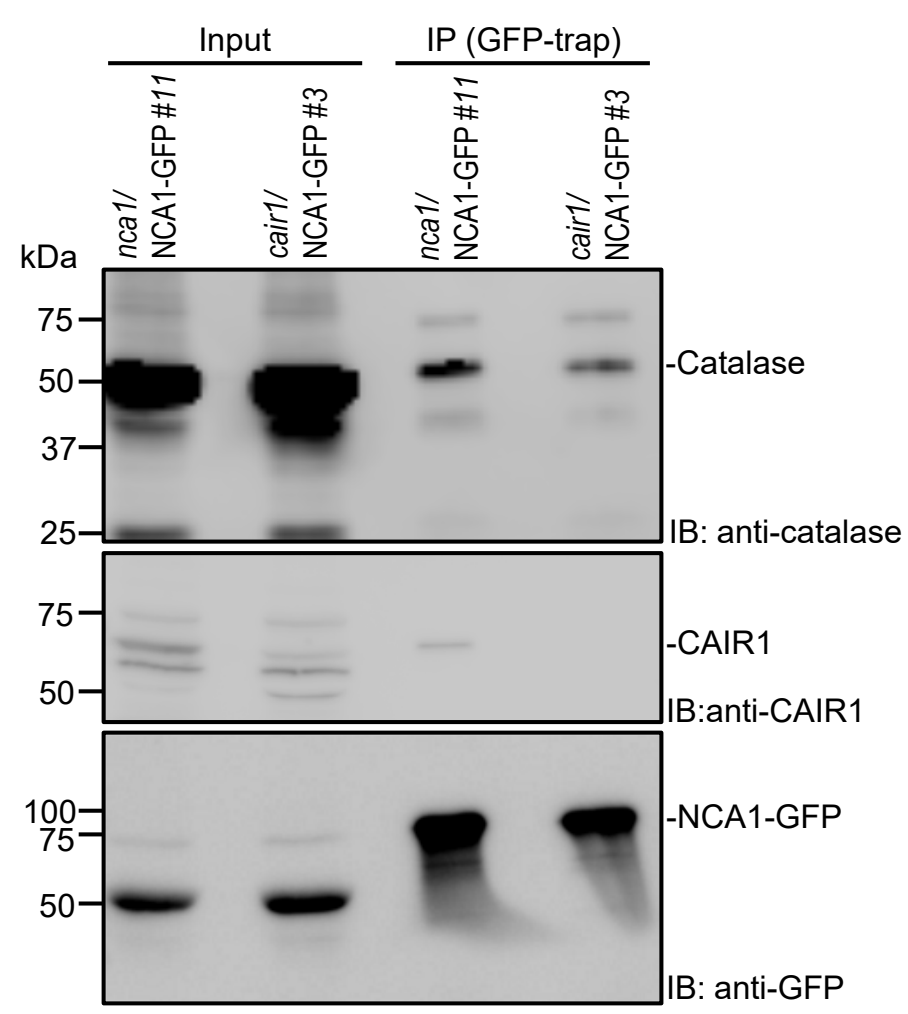

Supplementary Fig. 6. CAIR1 has little effect on the interaction of NCA1 with catalases.
Co-IP of endogenous catalase and CAIR1 using GFP-trap purification from extracts of 15-d-old *nca1-1*/Pro$_{NCA1}$:NCA1-GFP line #11 and *cair1-1*/Pro$_{NCA1}$:NCA1-GFP line #3 seedlings. This experiment was performed two times with similar results.

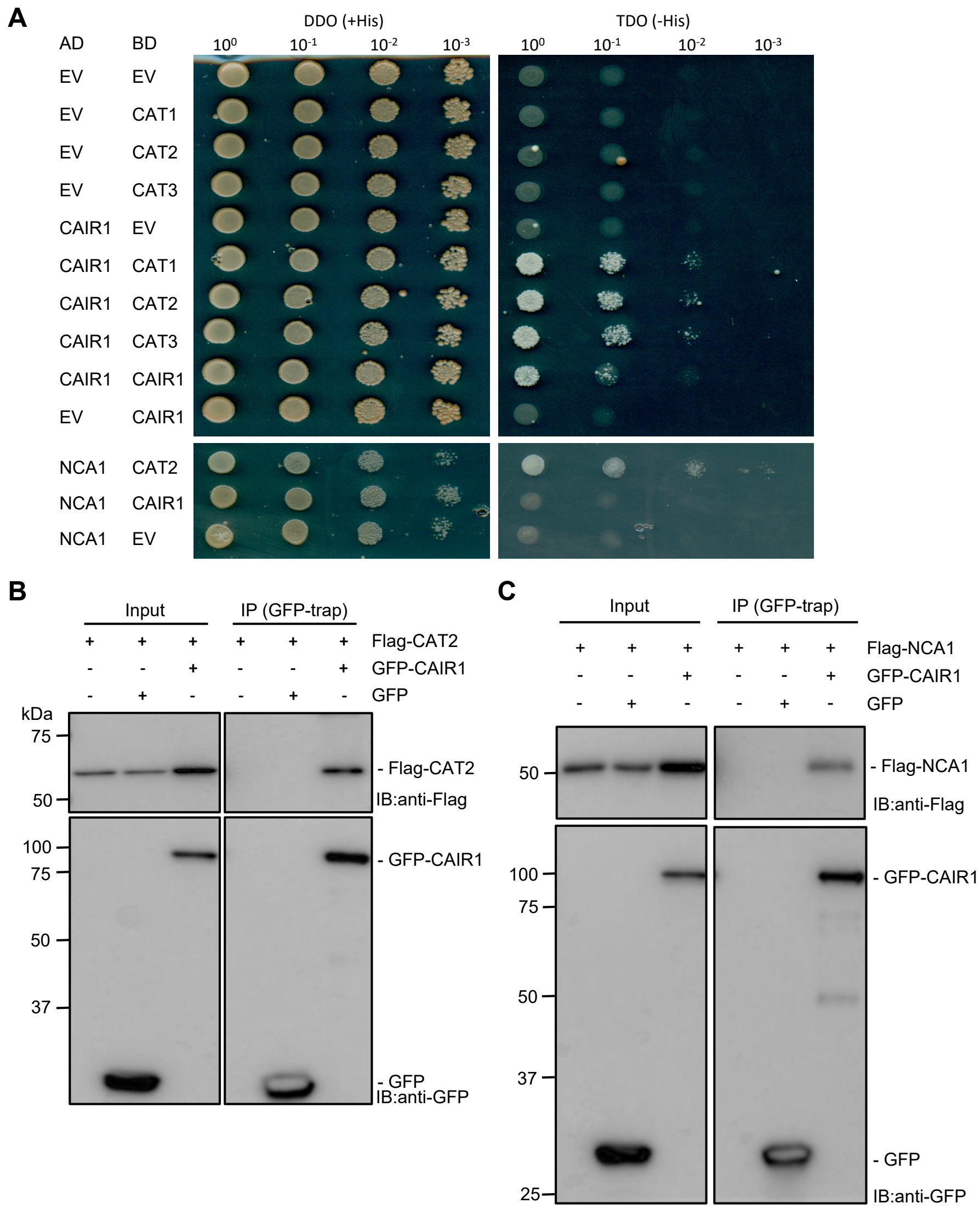

Supplementary Fig. 7. CAIR1 interacts with catalases and NCA1 in yeast and HEK293 cells.
(A) Yeast two-hybrid analysis of CAIR1, catalases CAT1, CAT2, CAT3, and NCA1. Tenfold serial dilutions of transformed yeast spotted on SD/−Trp/−Leu (DDO; nonselective for interaction) and SD/−Trp/−Leu/−His (TDO; selective) plates. AD, activation domain; BD, DNA binding domain; EV, empty vector. **(B, C)** HEK293T cells were co-transfected to express the indicated proteins, and GFP-CAIR1 and GFP (negative control) were immunoprecipitated using GFP-trap nanobodies. The IP signals (GFP-CAIR1 and GFP) and the co-IP signals (Flag-CAT2 and Flag-NCA1) were detected by immunoblots probed with antibodies against GFP- and Flag-epitopes, respectively. IP, immunoprecipitation; IB: immunoblotting.

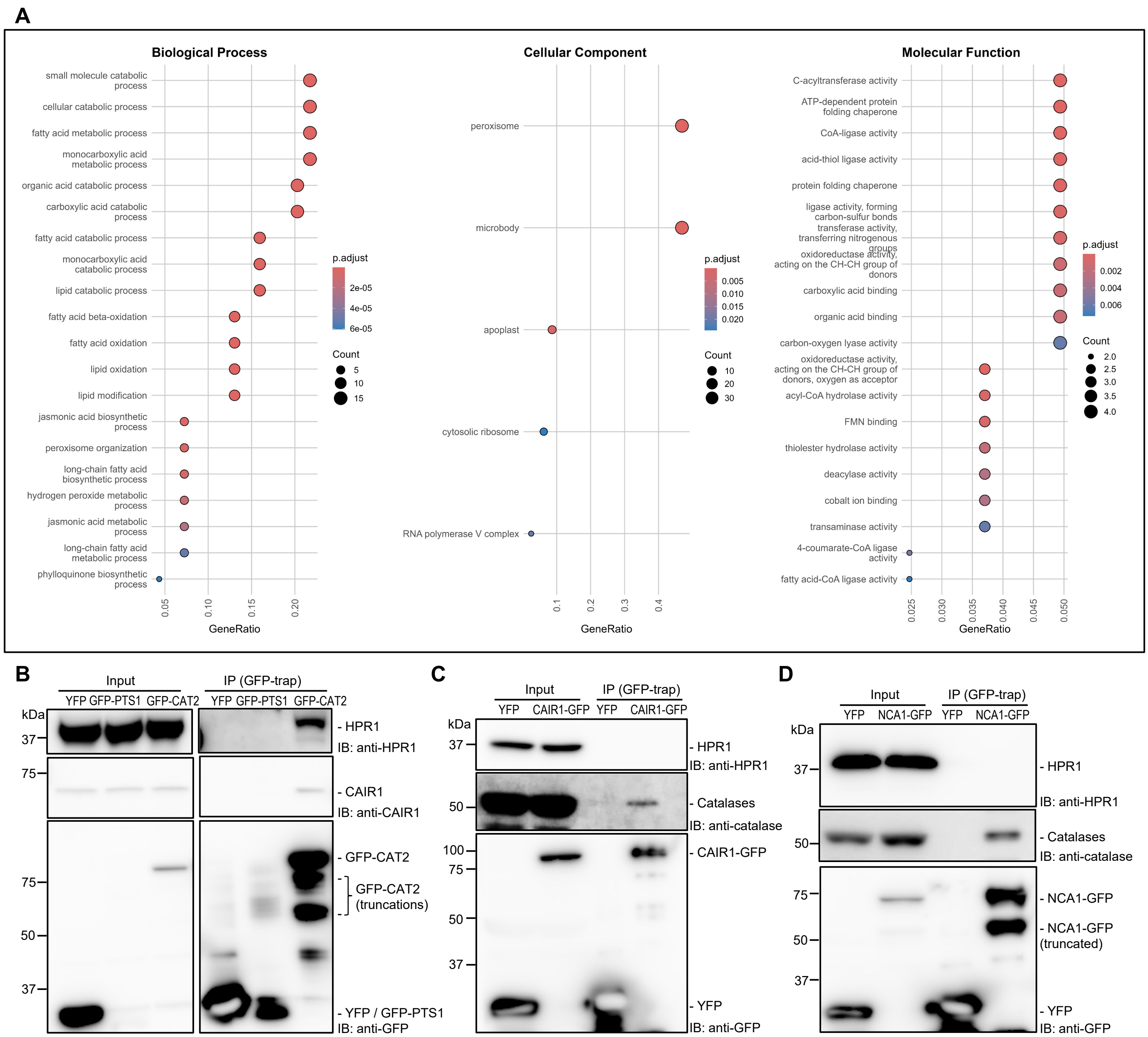

Supplementary Fig. 8. The interactome of CAT2 in Arabidopsis.
(A) Enriched GO terms in Biological Process, Cellular Component, and Molecular Function among GFP-CAT2 interaction candidates (Supplementary Data 1). **(B)** Co-IP of endogenous HPR1 and CAIR1 using GFP-trap purification from extracts of 15-d-old *cat2-2*/Pro$_{CAT2}$:GFP-CAT2 (GFP-CAT2) seedlings. **(C)** Co-IP of endogenous catalases but not HPR1 using GFP-trap purification from extracts of 15-d-old *cair1-1*/Pro$_{CAIR1}$:CAIR1-GFP (CAIR1-GFP) seedlings. **(D)** Co-IP of endogenous catalases but not HPR1 using GFP-trap purification from extracts of 15-d-old *nca1-1*/Pro$_{NCA1}$:NCA1-GFP (NCA1-GFP) seedlings. (A–C) Pro$_{35S}$:StrepII-3xHA-YFP (YFP) and/or Pro$_{35S}$:GFP-PTS1 (GFP-PTS1) lines were used as negative controls. IB, immunoblotting; IP, immunoprecipitation.

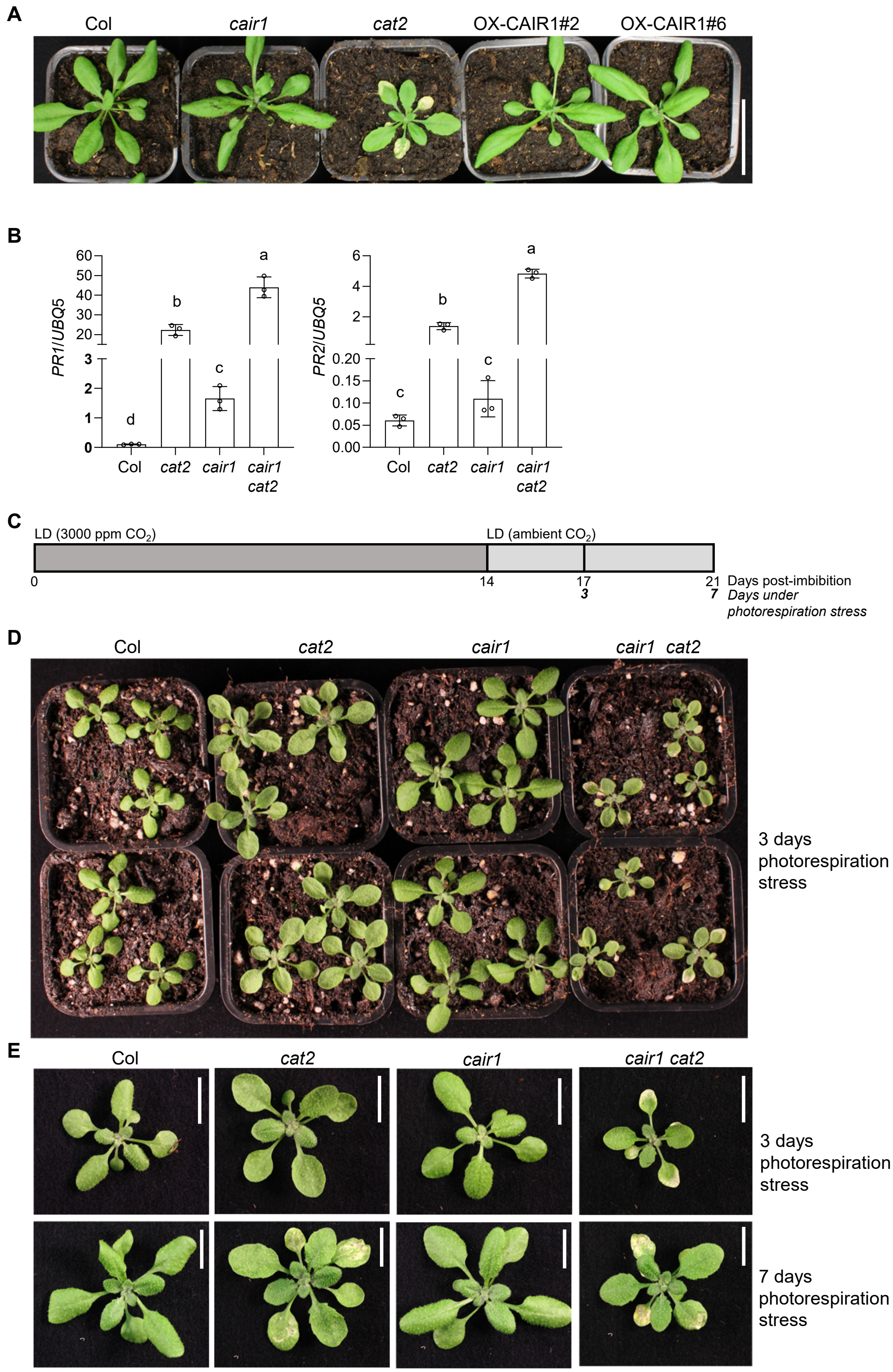

Supplementary Fig. 9. *cair1* enhances *cat2* leaf lesion phenotype under acute photorespiration stress in LD.
(A) Photographs of 15-d-old wild-type (Col), *cair1-1* (*cair1*), *cat2-2* (*cat2*), and two CAIR1 overexpression lines (OX-CAIR1 lines #2 and #6) plants grown on soil under long-day (LD) conditions. Scale bar: 3 cm. **(B)** RT-qPCR analysis of *PR1* and *PR2* gene expression in 15-d-old wild-type (Col), *cair1*, *cat2*, and *cair1 cat2* plants grown on soil under long-day (LD) conditions. Individual data points and means ± SD are shown (n = 3 biological replicates). **(C)** Scheme for acute photorespiration stress treatment. Plants were grown for two weeks in LD conditions under 3,000 ppm CO_2 and then transferred to a standard LD chamber with ambient CO_2 levels. **(D, E)** Photographs of Col, *cat2*, *cair1*, and *cair1-1 cat2-2* (*cair1 cat2*) plants 3 and 7 d after the transfer of 14-d-old plants to acute photorespiration stress as outlined in panel B. (C) Overview of plants 3 d after photorespiration stress. (D) Leaf lesions on individual plants on black background. Scale bars: 1 cm.

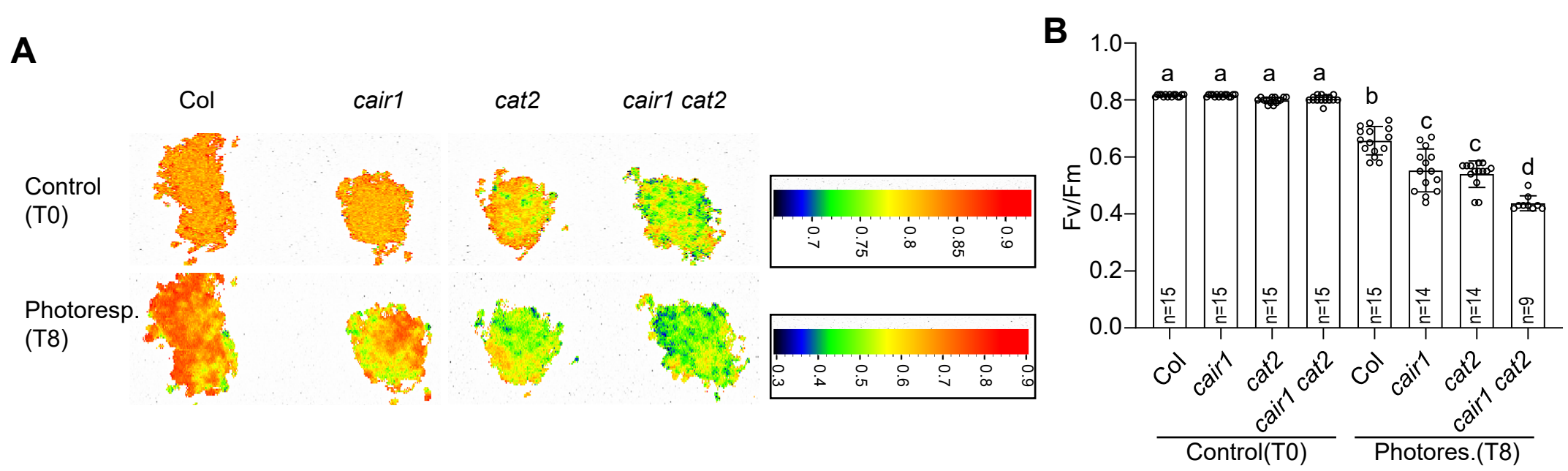

Supplementary Fig. 10. *cair1* exhibits sensitivity to oxidative stress.

(A) Response to photorespiration–promoting growth conditions. False-color images representing *F*v/*F*m values of 10-d-old wild-type (Col), *cair1-1* (*cair1*), *cat2-2* (*cat2*), and *cair1-1 cat2-2* (*cair1 cat2*) seedlings before and after 8-h exposure to photorespiration-promoting growth conditions. Triplicate replicate experiments were performed, and a representative plate is shown. **(B)** Quantitative *F*v/*F*m measurements of individual plants grown as described in panel (A). Individual data points and means ± SD are shown (n ≥ 9 plants). Shared letters indicate no significant differences among means (P > 0.05; two-way ANOVA, Tukey's test)..

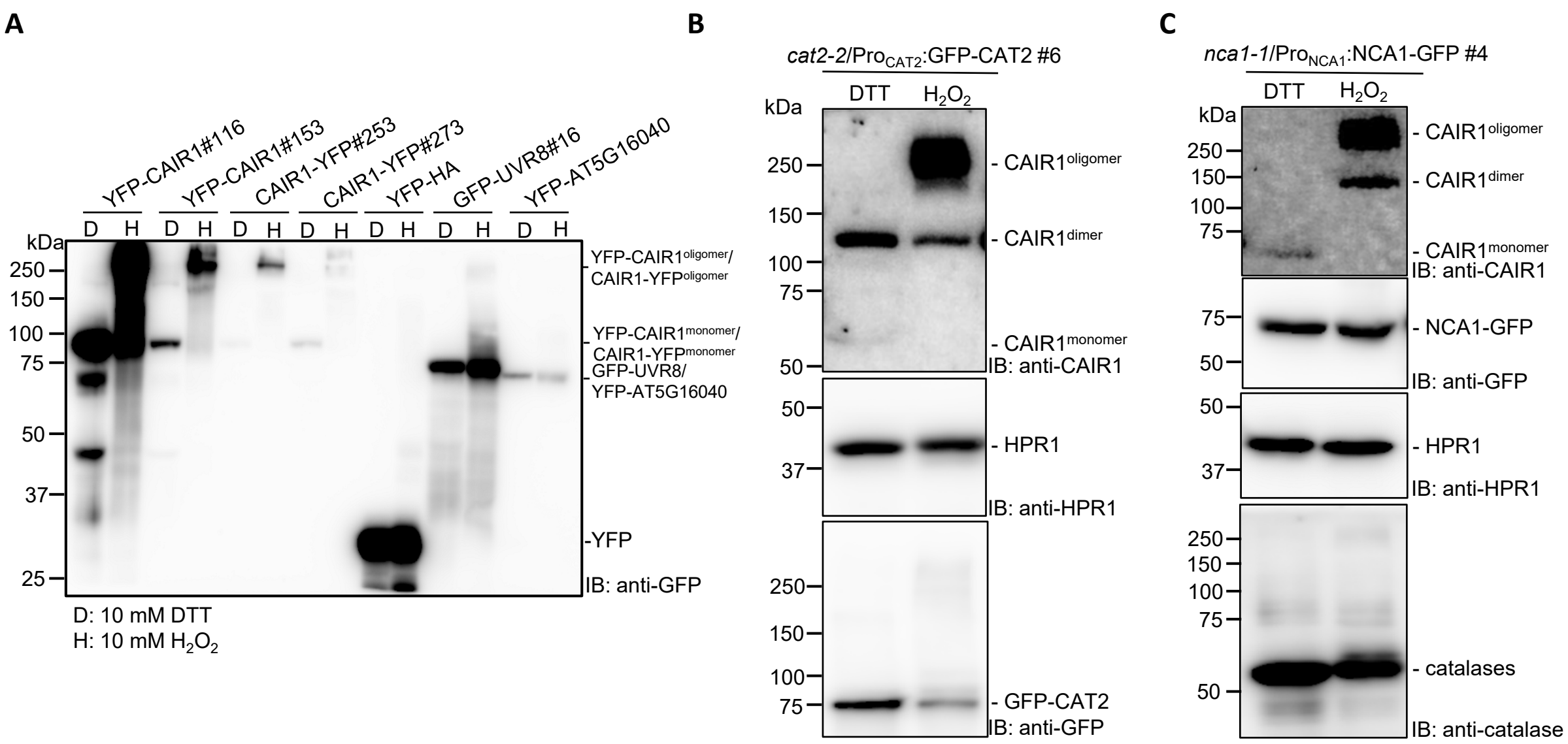

Supplementary Fig. 11. Specificity of redox–mediated CAIR1 oligomerization.

(A) YFP-CAIR1 shows oligomerization under oxidative conditions (10 mM H_2O_2), unlike the other tested YFP- or GFP-tagged proteins. Proteins were extracted from 5-d-old *cair1-1*/Pro_{35S}:YFP-CAIR1 #116 and #153, *cair1-1*/Pro_{35S}:CAIR1-YFP #253 and #273, Col/Pro_{35S}:YFP-HA, *uvr8-6*/Pro_{UVR8}:GFP-UVR8 #16, Col/Pro_{35S}:YFP-AT5G16040 seedlings. Then, the protein extracts were divided into two tubes to which 10 mM DTT (D) or 10 mM H_2O_2 (H) was added, respectively. **(B, C)** In contrast to CAIR1, no redox-dependent oligomerization of HPR1, GFP-CAT2, NCA1-GFP, or endogenous catalases was detected. Protein extracts from 5-d-old *cat2-2*/Pro_{CAT2}:GFP-CAT2 #6 and *nca1-1*/Pro_{NCA1}:NCA1-GFP #4 seedlings were treated with 10 mM DTT or 10 mM H_2O_2.

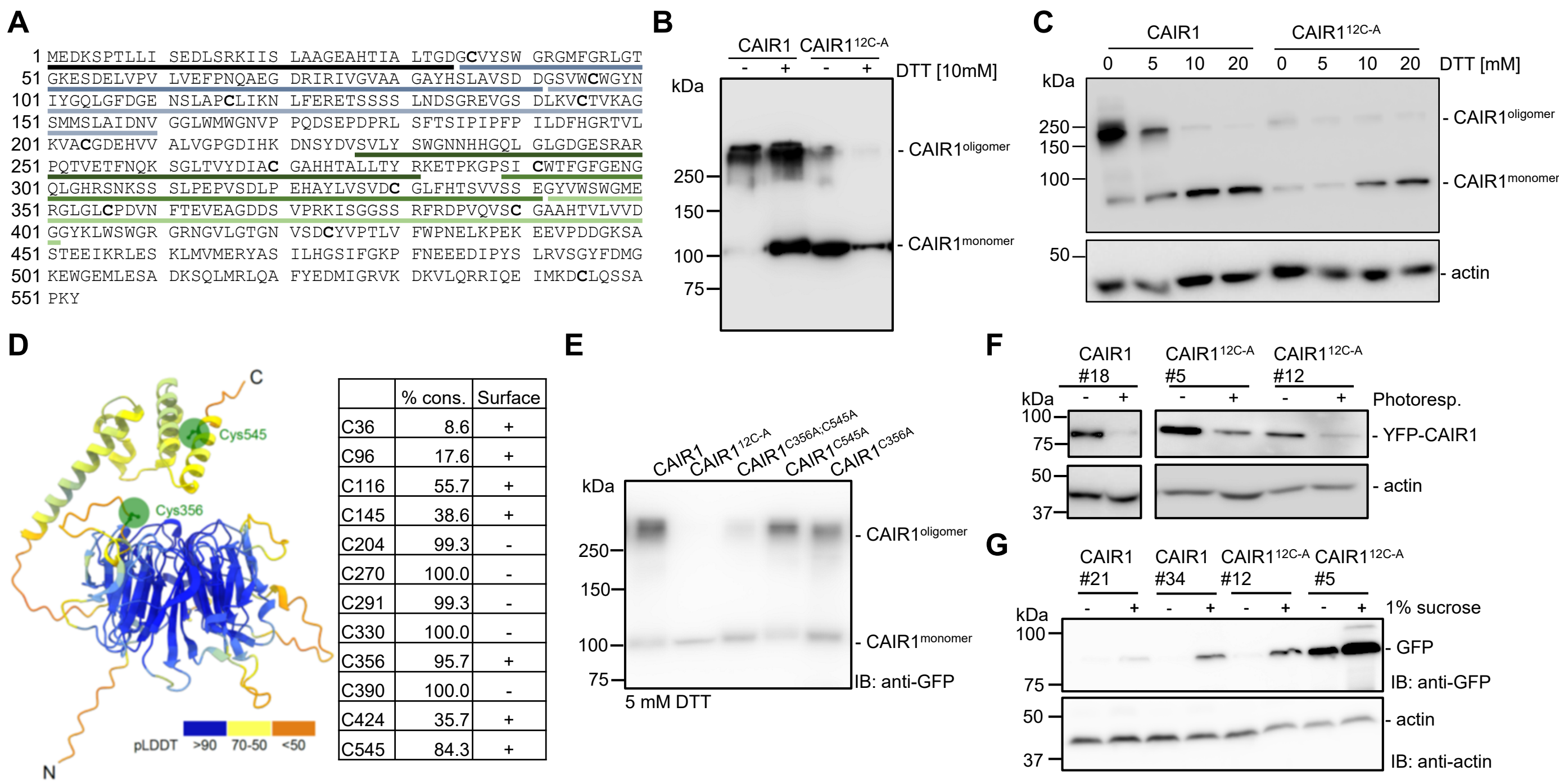

Supplementary Fig. 12. Redox-regulated oligomerization of CAIR1 depends on cysteines 356 and 545.
(A) CAIR1 contains 12 cysteine residues highlighted in bold. The six RLDs are underlined in different colors (see also Supplementary Fig. 2B). **(B)** YFP-CAIR1 (CAIR1) and YFP-CAIR1$^{12C\text{-}A}$ (CAIR1$^{12C\text{-}A}$) were transiently expressed in *N. benthamiana* and their oligomeric state in protein extracts in the presence of absence of 10 mM DTT was analyzed by anti-GFP immunoblot. **(C)** Proteins were extracted from 5-d-old *cair1-1*/Pro$_{35S}$:YFP-CAIR1 #33 (CAIR1) and *cair1-1*/Pro$_{35S}$:YFP-CAIR1$^{12C\text{-}A}$ #25 (CAIR1$^{12C\text{-}A}$) seedlings and CAIR1 oligomeric state in protein extracts containing different concentrations of DTT was analyzed by anti-GFP immunoblot. Actin levels are shown as loading control. **(D)** A ribbon diagram of the AlphaFold3 model of CAIR1 with surface-exposed cysteine 356 and 545 residues highlighted, alongside the % conservation of the 12 cysteine residues in plants. **(E)** YFP-CAIR1 variants were transiently expressed in *N. benthamiana* and their oligomeric state in protein extracts in the presence of 5 mM DTT was analyzed by anti-GFP immunoblot. **(F)** Mutation of the cysteine residues in CAIR1 did not alter ROS-mediated degradation of the protein. Six-d-old *cair1-1*/Pro$_{35S}$:YFP-CAIR1 #18, *cair1-1*/Pro$_{35S}$:YFP-CAIR1$^{12C\text{-}A}$ #12, and *cair1-1*/Pro35S:YFP-CAIR1$^{12C\text{-}A}$ #5 seedlings were transferred from a high CO_2 chamber to conditions promoting enhanced photorespiration for 4 d. **(G)** The cysteine residues in CAIR1 were not essential for its sucrose-dependent stability. Total proteins were extracted from 6-d-old *cair1-1*/Pro$_{35S}$:YFP-CAIR1 #21, *cair1-1*/Pro$_{35S}$:YFP-CAIR1 #34, *cair1-1*/Pro$_{35S}$:YFP-CAIR1$^{12C\text{-}A}$ #12, and *cair1-1*/Pro$_{35S}$:YFP-CAIR1$^{12C\text{-}A}$ #5 seedlings grown in the absence (-) or presence of 1% sucrose (+) in the growth medium.

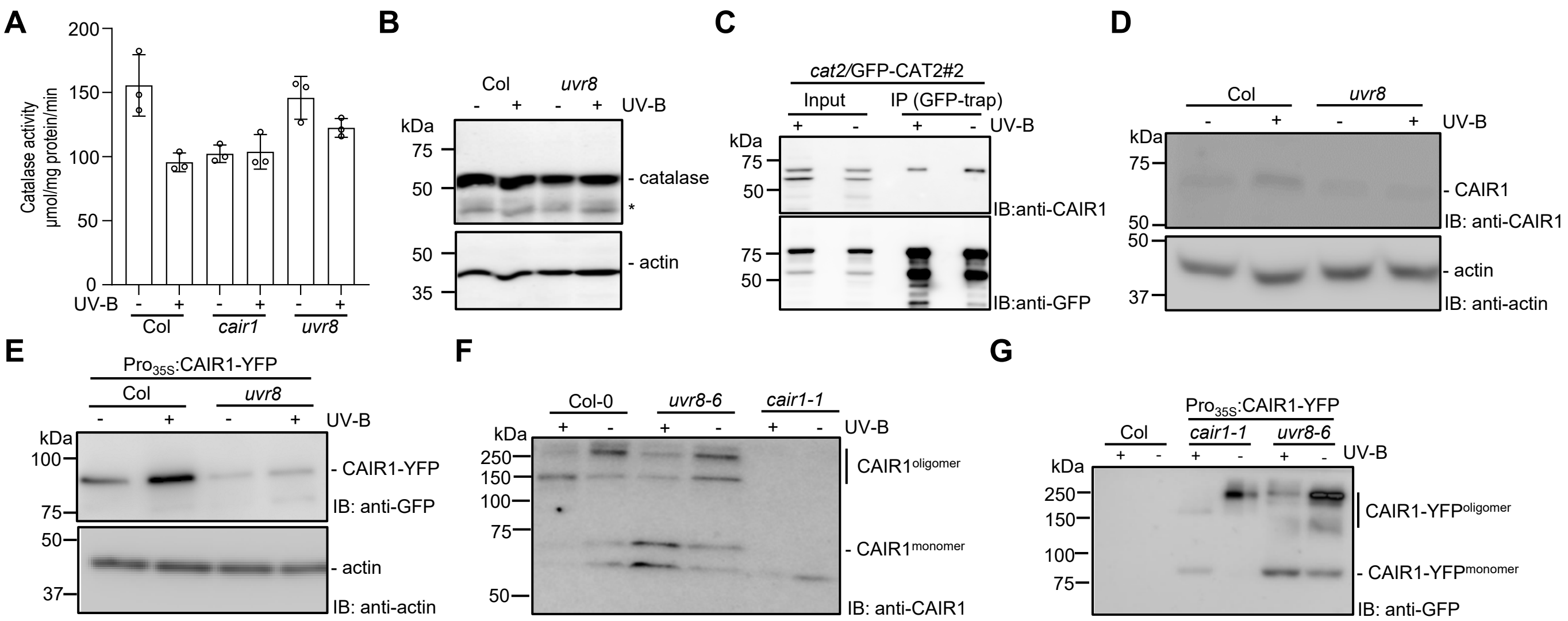

Supplementary Fig. 13. UV-B inhibits catalase activity through decreasing interaction of CAIR1 and catalase.

(A) Catalase activity was measured in protein extracts from 5-d-old wild-type (Col), *cair1-1* (*cair1*), and *uvr8-6* (*uvr8*) seedlings. Data shown are means ± SD (n = 3 independent experiments). Shared letters indicate no significant differences among means (P > 0.05; two-way ANOVA, Tukey's test). **(B)** Immunoblot analysis of catalase and actin (loading control) protein levels in 5-d-old Col and *uvr8-6* seedlings grown with or without supplementary UV-B. Asterisk (*) indicates an unspecific band. **(C)** UV-B decreases interaction of catalase and CAIR1 in planta. Proteins were extracted from 5-d-old *cat2-2*/Pro$_{CAT2}$:GFP-CAT2 #2 (*cat2*/GFP-CAT2#2) seedlings. **(D, E)** UV-B stabilizes CAIR1 in a UVR8-dependent manner. Proteins were extracted from 5-d-old (D) Col, *uvr8-6*, and (E) Col/Pro$_{35S}$:CAIR1-YFP and *uvr8-6*/Pro$_{35S}$:CAIR1-YFP seedlings. **(F)** Proteins were extracted from 5-d-old (D) Col, *uvr8-6* and *cair1-1* with lysis buffer with 1mM DTT. **(G)** Proteins were extracted from 5-d-old Col, *uvr8-6*/Pro$_{35S}$:CAIR1-YFP and *cair1-1*/Pro$_{35S}$:CAIR1-YFP with lysis buffer with 1mM DTT. (A–G) Seedlings were grown in white light (3.6 µmol m^{-2} s^{-1}) supplemented with UV-B (0.06 mW cm^{-2}) (**+**), or not (−).
