# Supplementary material for: A redox-regulated RCC1-like protein controls catalase activity in Arabidopsis
Source: Nat Commun. 2026 Aug 15;17:9807. doi: 10.1038/s41467-026-76792-x (PMC13575196; doi:10.1038/s41467-026-76792-x)
Supplement: Supplementary file 2 [file 41467_2026_76792_MOESM2_ESM.pdf]

Description of Additional Supplementary Files

Supplementary Data 1. List of CAIR1-, CAT2-, NCA1-, and UVR8-interacting proteins as identified in affinity purification–mass spectrometry.

Supplementary Data 2. Primers used in this study.

Supplementary Data 3. Summary of GFP-CAT2 affinity purification–mass spectrometry.
