# Supplementary material for: A redox-regulated RCC1-like protein controls catalase activity in Arabidopsis
Source: Nat Commun. 2026 Aug 15;17:9807. doi: 10.1038/s41467-026-76792-x (PMC13575196; doi:10.1038/s41467-026-76792-x)
Supplement: Supplementary file 3 [file 41467_2026_76792_MOESM6_ESM.pdf]

nature portfolio

Corresponding author(s): Roman Ulm

Last updated by author(s): Jun 15, 2026

Reporting Summary

Nature Portfolio wishes to improve the reproducibility of the work that we publish. This form provides structure for consistency and transparency in reporting. For further information on Nature Portfolio policies, see our Editorial Policies and the Editorial Policy Checklist.

Statistics

For all statistical analyses, confirm that the following items are present in the figure legend, table legend, main text, or Methods section.

n/a	Confirmed	
☐	☒	The exact sample size (*n*) for each experimental group/condition, given as a discrete number and unit of measurement
☐	☒	A statement on whether measurements were taken from distinct samples or whether the same sample was measured repeatedly
☐	☒	The statistical test(s) used AND whether they are one- or two-sided<br>*Only common tests should be described solely by name; describe more complex techniques in the Methods section.*
☒	☐	A description of all covariates tested
☒	☐	A description of any assumptions or corrections, such as tests of normality and adjustment for multiple comparisons
☐	☒	A full description of the statistical parameters including central tendency (e.g. means) or other basic estimates (e.g. regression coefficient) AND variation (e.g. standard deviation) or associated estimates of uncertainty (e.g. confidence intervals)
☐	☒	For null hypothesis testing, the test statistic (e.g. *F*, *t*, *r*) with confidence intervals, effect sizes, degrees of freedom and *P* value noted<br>*Give P values as exact values whenever suitable.*
☒	☐	For Bayesian analysis, information on the choice of priors and Markov chain Monte Carlo settings
☒	☐	For hierarchical and complex designs, identification of the appropriate level for tests and full reporting of outcomes
☒	☐	Estimates of effect sizes (e.g. Cohen's *d*, Pearson's *r*), indicating how they were calculated

Our web collection on statistics for biologists contains articles on many of the points above.

Software and code

Policy information about availability of computer code

Data collection	Fv/Fm values were collected with a FluorCam using FluorCam7 (version 1.2.5.3) software.<br>Root and hypocotyl length, as well as band intensities of western blots, were measured with ImageJ (1.50i).<br>Ct values in qRT-PCR experiments were calculated using Quantstudio Design and Analysis Software v1.4.3 (ThermoFisher).
Data analysis	Statistical analyses were performed using GGraphPad Prism (version 11.0.0).<br>MS raw data were processed with MaxQuant software (version 1.6.3.4) followed by statistical analysis with Perseus (version 1.5.8.5).<br>LB985 NightShade used indiGo software (version 2.0.5.0) for instrument control, scheduling, and image processing.<br>Phylogenetic analysis used MAFFT Multiple Sequence Alignment Software (version 7) and IQ-TREE 2. The phylogenetic tree was visualized using iTOL (version 7).<br>Visualization of domain structure and structure modeling used TBtools-II (v2.485), AlphaFold 3, and PyMOL (v2.5.2) .

For manuscripts utilizing custom algorithms or software that are central to the research but not yet described in published literature, software must be made available to editors and reviewers. We strongly encourage code deposition in a community repository (e.g. GitHub). See the Nature Portfolio guidelines for submitting code & software for further information.

Data

Policy information about availability of data

All manuscripts must include a data availability statement. This statement should provide the following information, where applicable:

- Accession codes, unique identifiers, or web links for publicly available datasets
- A description of any restrictions on data availability
- For clinical datasets or third party data, please ensure that the statement adheres to our policy

The data supporting the findings in this study are available and described within the paper and its supplementary files. The MS data have been deposited in the ProteomeXchange Consortium via the PRIDE partner repository with the dataset identifier PXD073328. Source data are provided with this paper.

Research involving human participants, their data, or biological material

Policy information about studies with human participants or human data. See also policy information about sex, gender (identity/presentation), and sexual orientation and race, ethnicity and racism.

Reporting on sex and gender	*Use the terms sex (biological attribute) and gender (shaped by social and cultural circumstances) carefully in order to avoid confusing both terms. Indicate if findings apply to only one sex or gender; describe whether sex and gender were considered in study design; whether sex and/or gender was determined based on self-reporting or assigned and methods used. Provide in the source data disaggregated sex and gender data, where this information has been collected, and if consent has been obtained for sharing of individual-level data; provide overall numbers in this Reporting Summary. Please state if this information has not been collected. Report sex- and gender-based analyses where performed, justify reasons for lack of sex- and gender-based analysis.*
Reporting on race, ethnicity, or other socially relevant groupings	*Please specify the socially constructed or socially relevant categorization variable(s) used in your manuscript and explain why they were used. Please note that such variables should not be used as proxies for other socially constructed/relevant variables (for example, race or ethnicity should not be used as a proxy for socioeconomic status). Provide clear definitions of the relevant terms used, how they were provided (by the participants/respondents, the researchers, or third parties), and the method(s) used to classify people into the different categories (e.g. self-report, census or administrative data, social media data, etc.) Please provide details about how you controlled for confounding variables in your analyses.*
Population characteristics	*Describe the covariate-relevant population characteristics of the human research participants (e.g. age, genotypic information, past and current diagnosis and treatment categories). If you filled out the behavioural & social sciences study design questions and have nothing to add here, write "See above."*
Recruitment	*Describe how participants were recruited. Outline any potential self-selection bias or other biases that may be present and how these are likely to impact results.*
Ethics oversight	*Identify the organization(s) that approved the study protocol.*

Note that full information on the approval of the study protocol must also be provided in the manuscript.

Field-specific reporting

Please select the one below that is the best fit for your research. If you are not sure, read the appropriate sections before making your selection.

☒ Life sciences ☐ Behavioural & social sciences ☐ Ecological, evolutionary & environmental sciences

For a reference copy of the document with all sections, see nature.com/documents/nr-reporting-summary-flat.pdf

Life sciences study design

All studies must disclose on these points even when the disclosure is negative.

Sample size	No statistical method was used to predetermine sample size. Sample sizes were selected based on established standards in the field and prior experience with Arabidopsis phenotypic assays. Sample sizes were chosen as large as possible while still practically doable in terms of data collection. Exact numbers of sample size n are indicated in the figures or figure legends.
Data exclusions	No data were excluded from analysis.
Replication	All experiments were replicated at least twice, with similar outcomes.
Randomization	No randomization was required for this study as the applied assays were not subject to inherent bias. However, position of plants was randomized when grown in growth rooms.
Blinding	Blinding was not applicable in this study because the experimentalist was responsible for preparing the samples and administering the treatments. Moreover, the study did not involve subjective outcome assessment. However, data analysis was standardized and automated

wherever possible to minimize the potential for bias.

Reporting for specific materials, systems and methods

We require information from authors about some types of materials, experimental systems and methods used in many studies. Here, indicate whether each material, system or method listed is relevant to your study. If you are not sure if a list item applies to your research, read the appropriate section before selecting a response.

Materials & experimental systems

n/a	Involved in the study	
☐	☒	Antibodies
☐	☒	Eukaryotic cell lines
☒	☐	Palaeontology and archaeology
☒	☐	Animals and other organisms
☒	☐	Clinical data
☒	☐	Dual use research of concern
☐	☒	Plants

Methods

n/a	Involved in the study	
☒	☐	ChIP-seq
☒	☐	Flow cytometry
☒	☐	MRI-based neuroimaging

Antibodies

Antibodies used	anti-HPR1 (1:10'000, Agrisera, AS11 1797), anti-Living Colors (1:5'000, Clontech, 632381), anti-HA (1:5'000, Biolegend, 902302), anti-catalase (1:1'000, Agrisera, AS09 501), anti-CAIR1 (1:1'000, Eurogentec, this work), anti-FLAG (1:5'000, Sigma, F3165), anti-UVR8 (1:1'000, Eurogentec, Favory et al., EMBO J. 2009), and anti-actin (1:10'000, Sigma, A0480) were used as primary antibodies in this study. Horseradish peroxidase (HRP)–conjugated anti-rabbit (1:10'000, Dako A/S, 41959817) and anti-mouse (1:10'000, Dako A/S, 41867249) immunoglobulins were used as secondary antibodies.
Validation	For all antibodies the validation data can be found in the manufacturer's website or in the cited reference (UVR8: Favory et al., 2009 - but also this work Supplementary Fig. 1B and E). Validation for the anti-CAIR1 antibody is provided in this work (e.g. Fig. 3B).

Eukaryotic cell lines

Policy information about cell lines and Sex and Gender in Research

Cell line source(s)	Flp-In T-REx HEK 293 cells (R78007, Invitrogen)
Authentication	None of the cell lines we used in this study were authenticated.
Mycoplasma contamination	All the cell lines used in this study tested negative for mycoplasma contamination.
Commonly misidentified lines (See ICLAC register)	Not applicable

Dual use research of concern

Policy information about dual use research of concern

Hazards

Could the accidental, deliberate or reckless misuse of agents or technologies generated in the work, or the application of information presented in the manuscript, pose a threat to:

No	Yes	
☒	☐	Public health
☒	☐	National security
☒	☐	Crops and/or livestock
☒	☐	Ecosystems
☒	☐	Any other significant area

Experiments of concern

Does the work involve any of these experiments of concern:

No	Yes	
☒	☐	Demonstrate how to render a vaccine ineffective
☒	☐	Confer resistance to therapeutically useful antibiotics or antiviral agents
☒	☐	Enhance the virulence of a pathogen or render a nonpathogen virulent
☒	☐	Increase transmissibility of a pathogen
☒	☐	Alter the host range of a pathogen
☒	☐	Enable evasion of diagnostic/detection modalities
☒	☐	Enable the weaponization of a biological agent or toxin
☒	☐	Any other potentially harmful combination of experiments and agents

Plants

Seed stocks

Seeds are available from the corresponding author upon reasonable request by email..

Novel plant genotypes

All transgenic lines (ProUVR8:GFP-UVR8/uvr8-6#16, #2OX-CAIR1, #6OX-CAIR1, OX-CAIR1-YFP#23, OX-YFP-CAIR1#343, OX-CAIR1-YFP#23/uvr8-6, OX-YFP-CAIR1#33, OX-YFP-CAIR1#25, HA-CAIR1, CAIR112C-A#12, CAIR112C-A#4, CAIR1C356545A#6, CAIR1C356545A#7, CAIR1C356A#14, CAIR1C545A#9, YFP-AT5G16040, ProCAT2:GFP-CAT2/cat2-2#2, ProCAT2:GFP-CAT2/cat2-2#6, ProCAT2:GFP-CAT2/cair1-1#1, ProCAT2:GFP-CAT2/cair1-1#2, ProCAIR1:CAIR1-GFP/cair1-1#25, ProCAIR1:CAIR1-GFP/cair1-1#33, ProNCA1:NAC1-GFP/nca1-1#4, ProNCA1:NAC1-GFP/nca1-1#11, PEP105P-CAIR1, PEP101P-CAIR1, PEP102P-CAIR1 and PEP118P-CAIR1) were generated by floral dip (Clough et al, 1998). Transgenic seeds were selected by antibiotics or fluorescent tags (Kalmbach et al, 2023), and at least 10 independent lines were analyzed per construct. Homozygous lines with single-locus insertion were identified and used in the T3 generation.
ProCAT2:GFP-CAT2/cat2 cair1, cair1 cat2, HA-CAIR1/CAIR1-YFP and PTS1/cair1-1 were generated by genetic crossing.
cair1-1 (GABI_438B07) was provided by the Nottingham Arabidopsis Stock Center (NASC). cair1-2 and cair1-3 were generated by CRISPR-Cas9 (Wang, et al 2015).

Authentication:
All novel overexpression and tagged lines were validated by western blotting and, when appropriate, by confocal microscopy. All T-DNA insertion lines were confirmed by PCR following cited references (cair1-1: this study, cat2-2: Queval et al, 2009, nca1-1: Hackenberg et al, 2013, uvr8-6: Favory et al, 2009, rup1rup2: Gruber et al, 2010). cair1-2 and cair1-3 were confirmed by PCR and sequencing. Pro35S:StrepII-3xHA-YFP and Pro35S:GFP-PTS1 were confirmed by western blot and confocal microscopy.

Authentication
