# Supplementary material for: A redox-regulated RCC1-like protein controls catalase activity in Arabidopsis
Source: Nat Commun. 2026 Aug 15;17:9807. doi: 10.1038/s41467-026-76792-x (PMC13575196; doi:10.1038/s41467-026-76792-x)
Supplement: Supplementary file 4 [file 41467_2026_76792_MOESM7_ESM.pdf]

nature portfolio

Peer Review File

A redox-regulated RCC1-like protein controls catalase activity in Arabidopsis

Corresponding Author: Professor Roman Ulm

This file contains all reviewer reports in order by version, followed by all author rebuttals in order by version.

Version 0:

Reviewer comments:

Reviewer #1

(Remarks to the Author)
A redox regulated RCC1-like protein controls catalase activity in Arabidopsis.
Zhoubo Hu et al.
Significance: Catalase is one of the most important enzymic anti-oxidants which protects against excessive oxidation and regulates oxidative signalling. Despite its importance there are still unknowns regarding its biogenesis and regulation of targeting. This is a clearly written paper which identifies and characterises a novel protein interactor of Arabidopsis thaliana catalases that controls their activity in a redox dependent manner.

Key findings:
CAIR1, an unknown member of the RCC1-like protein family, was identified as an interactor of the UVR8 photoreceptor. CAIR1 was shown by pull down to interact in vivo with all 3 Arabidopsis catalases and NCA1, a catalase chaperone. CAIR1:CAT2 interaction was validated by Y2H and CAIR1:CAT2 and CAIR1:NCA1 by split luciferase. Affinity pulldowns with CAT2, NCA1 or CAIR1 as baits pulled down overlapping sets of proteins with the 3 Arabidopsis catalases, CAIR1 and NCA1 as constants, suggesting they form a complex. CAT pulled down many peroxisomal proteins which were not recovered by the other baits. CAIR 1 was shown by sfGFP to be nuclear and cytosolic, and to interact with CAT2 in the cytosol by BiFC. CAIR1mutants showed reduced catalase activity which was rescued by CAIR1 complementation. Reduced activity was not due to reduced protein level but to increased aggregation. CAIR1 mutants show sensitivity to enhanced photorespiratory conditions and upregulation of hydrogen peroxide induced marker genes, as well as enhanced sensitivity to alkaline pH. Cair1 cat2 and nca1 mutants all show reduced root length. CAIR1 protein is degraded under enhanced photorespiratory conditions and by exogenous H_2O_2, and is stabilised by high CO_2 and proteosome inhibitors. CAIR1 self-interacts and this is dependent upon redox conditions. Enhanced photorespiration increased interaction between CAIR1 and CAT and resulted in increased cytosolic localisation of CAT. The redox sensitivity of CAIR1 is driven by its Cys of which C356 and C545 are important and catalase interaction is weakened in these mutants. Fv/Fm values are also reduced in these mutants after enhanced photorespiration. Plants grown under UVB showed reduced CAIR1-CAT2 interaction and reduced catalase activity
The data are high quality with appropriate interpretation and controls. The paper is well organised making it straightforward to follow. I have only a few minor points:
1. Fig 6H why is there so much degradation of GFPCAT2 in this experiment?
2. Line 355 make clear CAIR12C-A is (I presume) mutation of all 12 cys (as there is no Cys12)
3. Line 559 I think the authors mean CAT2 not CAIR1. Please check
4. Is CAIR1 an Arabidopsis specific or plant specific protein? It might be interesting for the authors to comment on this

Reviewer #2

(Remarks to the Author)
This study identifies CAIR1, a previously uncharacterized RCC1-like protein, as a regulator of catalase activity in Arabidopsis. The authors demonstrate that CAIR1 interacts with catalases and their chaperone NCA1, and undergoes redox-dependent oligomerization to modulate CAT2 peroxisomal import and activity. They also show that UV-B signaling regulates catalase activity through CAIR1-UVR8 interaction. The work is comprehensive, well-executed, and provides significant new insights into redox regulation of H_2O_2 homeostasis. However, several issues need to be addressed before

publication.
1. While the manuscript establishes that CAIR1 interacts with NCA1 and CAT2, it lacks critical mechanistic evidence explaining how CAIR1 specifically regulates the assembly, stability, or trafficking of the NCA1-CAT2 complex. Whether CAIR1 influences NCA1's chaperone efficiency or facilitates CAT2 release ?
2. The authors showed that UV-B lowers catalase activity by weakening the CAIR1-CAT2 interaction in a UVR8-dependent way. Whether UV-B affects CAIR1 oligomerization or if UVR8 plays a role in its redox modification?
3.Figure 2C shows that CAIR is localized in both the nucleus and the cytoplasm, so why is only cytoplasmic localization observed in Figure 2B?
4. In Figure 2D, the punctate signals from the HPR1-CAT2 interaction are inferred to be peroxisomes but lack validation with a peroxisomal marker. Besides, Figure 3C also lack peroxisomal marker. Co-expression of a peroxisome marker is recommended to confirm the peroxisomal localization.
5.Figure 3B shows increased catalase protein in both cair1 mutants and CAIR1-OE lines compared to Col-0, despite opposite activity changes. It is recommended to add a brief explanation in the Results or Discussion to clarify this pattern.
6. In Supplemental Figure S9A, under the control condition, the cat2, cair1cat2 mutants appear to differ from the wild type, but no significant difference is observed in the corresponding quantitative analysis. This is rather confusing. Besides, the differences in ROS levels shown in Figure S9C are difficult to distinguish. Please use other methods to quantify the ROS levels.
7. In Figure 4F, cair1 showed reduced primary root growth compared to that in wild type at pH 8.5, but in Figure 4H, the root length of cair1mutant appears to be longer than that of the wild type. It is recommended that the root length need to be statistically analyzed under pH 9.0 conditions.
8.Figure 5C lacks a mock-treated time course as a necessary control to track CAIR1's basal oligomeric state and rule out non-specific effects. The same issue applies to Figure 5B.
9.Figure 6H shows enhanced CAIR1-CAT2 interaction under oxidative stress, but it doesn't directly check CAIR1 oligomerization in the same samples. It is recommended to assess the oligomeric state of CAIR1 in the same samples.
Minor:
1. L165, the lack of CAIR1-NCA1 interaction in yeast two-hybrid assays contradicts the positive interactions observed in LCI and Co-IP assays. Please explain this discrepancy. Is the interaction indirect (e.g., bridged by CAT2)?
2. Since anti-catalase antibody detect all three catalases (CAT1, CAT2, and CAT3), why was no catalase band detected in the cat2-2 mutant in Figure S5?
3. L181, the authors include GFP-PTS1 (a peroxisomal marker line) as a negative control in Figure 1C. It is better to explain the role of GFP-PTS1 when describing Figure 1C in the Results section.
3.Many western blot images appear overexposed of the protein band, possibly due to high protein concentrations, as in Figure 1D, Figure 6C, 6G, 6H and other panels in Supplemental Figures. Please optimize the images.

4. In Figures 5B, D, E,F and 5G, the actin band intensities are inconsistent. It is suggested to perform consistent quantification and conduct statistical analysis.

Reviewer #3

(Remarks to the Author)
The paper by Hu et al. identifies CAIR1 (AT5G11580), a previously uncharacterized RCC1-like protein in Arabidopsis, as a cytosolic regulator of catalase activity. The authors have unequivocally demonstrated using a diverse breadth of orthogonal assays that CAIR1 interacts with all three catalase isoforms and the chaperone NCA1 in the cytosol. Moreover, CAIR1 undergoes reversible, redox-dependent oligomerization via Cys-356 and Cys-545 that enhances its interaction with catalases under oxidative stress. The paper connects CAIR1 function to photorespiration stress, alkaline pH sensitivity, sucrose-dependent root elongation, and UV-B responses.

The mechanistic model for how CAIR1 controls peroxisomal import remains speculative. The central claim is that CAIR1 regulates catalase activity by facilitating peroxisomal import, yet there is no direct measurement of import rates. The evidence that GFP-CAT2 forms aggregates in cair1 is consistent with this model, but aggregation could also reflect misfolding, loss of cytosolic chaperone function, impaired tetramerization and CAT oxidation— none of which are distinguished. As it stands, the import model is plausible but not demonstrated. Along these lines, further exploration of whether CAIR1 affects the redox state of catalases could be informative.

In Figure 6K, after 48 hours of enhanced photorespiration, catalase activity does not show statistically significant differences between WT and cair1. This result may nuance the interpretation of CAIR1's role in photorespiratory catalase regulation. Additionally, the effect of DTT on CAIR1-dependent catalase oligomerization is not examined and could provide useful insight into the redox regulation mechanism.

The UVR8–CAIR1 connection remains poorly resolved. CAIR1 was identified as a UVR8 co-purifying protein, which motivates the study, yet the authors show that CAIR1 does not participate in canonical UVR8 signaling (Supplemental Figure S4). The UV-B section (lines 367–381) demonstrates reduced catalase activity under UV-B and weakened CAIR1–CAT2 interaction, but the mechanistic connection is vague. The authors themselves acknowledge they cannot distinguish

whether UVR8 indirectly suppresses the CAIR1–CAT2 interaction (via altered ROS levels) or directly disrupts it. This section feels underdeveloped relative to its prominence in the narrative.

The AP–MS data show that CAIR1 co-purifies with both UVR8 and catalases, yet UVR8 and CAT never appear in each other's purifications (lines 196–200). If CAIR1 bridges these two complexes, the absence of a ternary complex is important to reconcile. The authors mention this observation but do not discuss its implications for the proposed model.

The CAIR1 12C-A (all 12 cysteines mutated) and CAIR1 C356A,C545A mutants both abolish redox-dependent oligomerization, but the single-cysteine mutants (CAIR1 C356A and CAIR1 C545A) are only tested for oligomerization (Figure 7A) and not for physiological rescue or catalase interaction in planta. This leaves open whether one or both cysteines are individually required for function, or whether additional cysteines contribute.

Total cellular catalase activity is measured from crude extracts, which cannot distinguish between cytosolic and peroxisomal pools. Since the model proposes that CAIR1 enhances catalase activity by promoting peroxisomal import, it would be important to measure catalase activity in fractionated subcellular compartments to confirm that the activity reduction in cair1 specifically reflects loss of peroxisomal catalase. Complementary time-lapse confocal imaging of CAT-GFP import into peroxisomes—using lines expressing a fluorescent peroxisomal marker—could also provide direct evidence supporting the proposed import mechanism.

Minor comments

CAIR1 and NCA1 co-purify and both interact with catalases, yet the Y2H assay shows no direct CAIR1–NCA1 interaction (line 165). Does CAIR1 require NCA1 for its function? Does NCA1 still function in cair1 mutants? The epistatic relationship is barely explored. The cair1 mutants partially phenocopy cat2 and nca1, but the mechanistic distinction between CAIR1 and NCA1 contributions to catalase regulation is left unclear.

Reviewer #4

(Remarks to the Author)
I co-reviewed this manuscript with one of the reviewers who provided the listed reports. This is part of the Nature Communications initiative to facilitate training in peer review and to provide appropriate recognition for Early Career Researchers who co-review manuscripts.

Version 1:

Reviewer comments:

Reviewer #1

(Remarks to the Author)
The authors have satisfactorily addressed my concerns in this revised manuscript

Reviewer #2

(Remarks to the Author)
The authors have addressed most of the concerns raised in the previous round of review and the manuscript has been improved accordingly. However, a few issues remain that require further clarification before the manuscript can be accepted for publication.

1. In the new added Supplementary Figure S6, the Input blots probed with the CAIR1 antibody show numerous non-specific bands, making it difficult to identify the specific CAIR1 signal. Moreover, the co-immunoprecipitated catalase levels appear markedly different between the two groups, which should be explained. Whether CAIR1 affects the NCA1-catalase interaction needs further discussion.

2. In Figures 5B, D and G, the signal intensities of Actin loading control bands are inconsistent. To ensure the reliability of quantitative results, we suggest the authors repeat the Western blot experiments with equal total protein loading across all samples, and then normalize the signal intensities of target protein bands against Actin for quantitative analysis.

Reviewer #3

(Remarks to the Author)
The manuscript can be accepted for publication

Open Access This Peer Review File is licensed under a Creative Commons Attribution 4.0 International License, which permits use, sharing, adaptation, distribution and reproduction in any medium or format, as long as you give appropriate credit to the original author(s) and the source, provide a link to the Creative Commons license, and indicate if changes were made.
In cases where reviewers are anonymous, credit should be given to 'Anonymous Referee' and the source.
The images or other third party material in this Peer Review File are included in the article's Creative Commons license, unless indicated otherwise in a credit line to the material. If material is not included in the article's Creative Commons license and your intended use is not permitted by statutory regulation or exceeds the permitted use, you will need to obtain permission directly from the copyright holder.
To view a copy of this license, visit https://creativecommons.org/licenses/by/4.0/

Point-by-point response to reviewers' comments:

Reviewer #1 (Remarks to the Author)

A redox regulated RCC1-like protein controls catalase activity in Arabidopsis.
Zhoubo Hu et al.
Significance: Catalase is one of the most important enzymic anti-oxidants which protects against excessive oxidation and regulates oxidative signalling. Despite its importance there are still unknowns regarding its biogenesis and regulation of targeting. This is a clearly written paper which identifies and characterises a novel protein interactor of Arabidopsis thaliana catalases that controls their activity in a redox dependent manner.

Key findings:
CAIR1, an unknown member of the RCC1-like protein family, was identified as an interactor of the UVR8 photoreceptor. CAIR1 was shown by pull down to interact in vivo with all 3 Arabidopsis catalases and NCA1, a catalase chaperone. CAIR1:CAT2 interaction was validated by Y2H and CAIR1:CAT2 and CAIR1:NCA1 by split luciferase. Affinity pulldowns with CAT2, NCA1 or CAIR1 as baits pulled down overlapping sets of proteins with the 3 Arabidopsis catalases, CAIR1 and NCA1 as constants, suggesting they form a complex. CAT pulled down many peroxisomal proteins which were not recovered by the other baits. CAIR 1 was shown by sfGFP to be nuclear and cytosolic, and to interact with CAT2 in the cytosol by BiFC. CAIR1mutants showed reduced catalase activity which was rescued by CAIR1 complementation. Reduced activity was not due to reduced protein level but to increased aggregation. CAIR1 mutants show sensitivity to enhanced photorespiratory conditions and upregulation of hydrogen peroxide induced marker genes, as well as enhanced sensitivity to alkaline pH. Cair1 cat2 and nca1 mutants all show reduced root length. CAIR1 protein is degraded under enhanced photorespiratory conditions and by exogenous H_2O_2, and is stabilised by high CO_2 and proteosome inhibitors. CAIR1 self-interacts and this is dependent upon redox conditions. Enhanced photorespiration increased interaction between CAIR1 and CAT and resulted in increased cytosolic localisation of CAT. The redox sensitivity of CAIR1 is driven by its Cys of which C356 and C545 are important and catalase interaction is weakened in these mutants. Fv/Fm values are also reduced in these mutants after enhanced photorespiration. Plants grown under UVB showed reduced CAIR1-CAT2 interaction and reduced catalase activity

The data are high quality with appropriate interpretation and controls. The paper is well organised making it straightforward to follow. I have only a few minor points:

- *Response :*
Many thanks for the positive feedback on our manuscript.

1. Fig 6H why is there so much degradation of GFPCAT2 in this experiment?

- *Response :*
In comparison to the co-IPs shown in Fig. 1C and Supplementary Fig. 8B, which already showed substantial GFP-CAT2 degradation products during IPs, the co-IP experiment shown in Fig. 6H was performed in the absence of DTT in the lysis buffer, which apparently additionally negatively affected GFP-CAT2 stability during the incubation time needed for the IP procedure.

2. Line 355 make clear CAIR12C-A is (I presume) mutation of all 12 cys (as there is no Cys12)

- *Response :*

We clarified this point and lines 378-380 (numbering according to the marked-up version) now read: "However, CAIR1$^{12C\text{-}A}$ with all 12 cysteines mutated to alanines still showed ROS-mediated CAIR1 degradation and sucrose-mediated CAIR1 stabilization (Supplementary Fig. 12F, G).".

3. Line 559 I think the authors mean CAT2 not CAIR1. Please check

- Response :
Line 603: corrected.

4. Is CAIR1 an Arabidopsis specific or plant specific protein? It might be interesting for the authors to comment on this

- Response :
New Supplementary Fig. 2C: We investigated the conservation of CAIR1 in plants (Viridiplantae) and generated a phylogenetic tree of 15 RCC1-family members (excluding the 9 PRAF proteins). Lines 135–140 now read: "Phylogenetic analysis of CAIR1 protein sequences reveals that orthologs are present as one or two copies in angiosperms, gymnosperms, and ferns (Supplementary Fig. 2C). In contrast, no putative orthologs were detected in the chlorophytes, liverworts, mosses, and lycophytes sampled in this study (Supplementary Fig. 2C); however, broader taxonomic sampling and additional genomic resources will be required to fully assess its distribution across early-diverging plant lineages"

Reviewer #2 (Remarks to the Author)

This study identifies CAIR1, a previously uncharacterized RCC1-like protein, as a regulator of catalase activity in Arabidopsis. The authors demonstrate that CAIR1 interacts with catalases and their chaperone NCA1, and undergoes redox-dependent oligomerization to modulate CAT2 peroxisomal import and activity. They also show that UV-B signaling regulates catalase activity through CAIR1-UVR8 interaction. The work is comprehensive, well-executed, and provides significant new insights into redox regulation of H_2O_2 homeostasis. However, several issues need to be addressed before publication.

- Response :
Many thanks for the positive evaluation of our manuscript.

1. While the manuscript establishes that CAIR1 interacts with NCA1 and CAT2, it lacks critical mechanistic evidence explaining how CAIR1 specifically regulates the assembly, stability, or trafficking of the NCA1-CAT2 complex. Whether CAIR1 influences NCA1's chaperone efficiency or facilitates CAT2 release?

- Response :
New Supplementary Fig. 6: We performed a new experiment testing whether CAIR1 affects the NCA1-CAT2 interaction but found no clear evidence for such a mechanism. Lines 174-177 (numbering according to the marked-up version) now read "We further tested whether CAIR1 may affect the NCA1-catalase interaction using a cair1-1/Pro$_{NCA1}$:NCA1-GFP line. However, absence of CAIR1 did not affect co-IP of catalases with NCA1-GFP (Supplementary Fig. 6)."

Regarding whether CAIR1 affects NCA1 activity it should be noted that in contrast to nca1, neither the cair1 mutant nor CAIR1 overexpression (OX-CAIR1) line influenced catalase protein levels, suggesting that CAIR1 has no major role in regulating NCA1 chaperone activity. Lines 439-445 now read "Our data indicate that the CAIR1–CAT2–NCA1 complex localizes to the cytosol, where NCA1 functions as a chaperone required for catalase folding and stability [40-42]. Interestingly, CAIR1 apparently does not affect the interaction of NCA1 with catalases and catalase protein levels are not altered in cair1 or a CAIR1-overexpression line. These observations suggest that CAIR1

plays no major role in NCA1-mediated catalase folding or stability but likely affects peroxisomal import subsequently; however, the exact molecular mechanism remains to be determined.".

2. The authors showed that UV-B lowers catalase activity by weakening the CAIR1-CAT2 interaction in a UVR8-dependent way. Whether UV-B affects CAIR1 oligomerization or if UVR8 plays a role in its redox modification?

- Response :
New Supplementary Fig. 13F and G: CAIR1 indeed showed reduced oligomerization under UV-B and this reduction was partially dependent on UVR8. Lines 404-406 now read "In contrast, CAIR1 and CAIR1-YFP oligomerized less under UV-B and this reduction was partially dependent on UVR8 (Supplementary Fig. 13F, G)."

3.Figure 2C shows that CAIR is localized in both the nucleus and the cytoplasm, so why is only cytoplasmic localization observed in Figure 2B?

- Response :
In Fig. 2C we used a self-assembling split superfolder GFP (sfGFP) system, which is more sensitive to detect subcellular localization than standard CAIR1-GFP confocal microscopy used in Fig. 2B. Thus, the sfGFP approach allowed detection of minor amounts of nuclear CAIR1. This suggests that the nuclear abundance of CAIR1 is low and not readily detectable by CAIR1-GFP fluorescence microscopy. If and how nuclear CAIR1 plays a functional role is out of scope for this work but will be a focus of future research.

4. In Figure 2D, the punctate signals from the HPR1-CAT2 interaction are inferred to be peroxisomes but lack validation with a peroxisomal marker. Besides, Figure 3C also lack peroxisomal marker. Co-expression of a peroxisome marker is recommended to confirm the peroxisomal localization.

- Response :
New Fig. 2D: We repeated the BiFC experiments including a co-expressed peroxisomal marker mCherry-PTS1, clearly confirming that the reconstituted YN-CAT2 + YC-HPR1 signals overlap with the peroxisomal marker, in sharp contrast with the YN-CAT2 + YC-CAIR1 signals.

Fig. 3C: Although we agree with the recommendation of the reviewer, unfortunately we do not have transgenic lines expressing mCherry-PTS1 as peroxisome marker in the cat2/Pro$_{CAT2}$:GFP-CAT2 and cair1 cat2/Pro$_{CAT2}$:GFP-CAT2 backgrounds. To generate such lines by introducing additionally a Pro$_{35S}$:mCherry-PTS1 transgene would delay publication several months, which, also in view of the established peroxisomal localization of CAT2, we consider out of scope for this publication.

5.Figure 3B shows increased catalase protein in both cair1 mutants and CAIR1-OE lines compared to Col-0, despite opposite activity changes. It is recommended to add a brief explanation in the Results or Discussion to clarify this pattern.

- Response :
New Supplementary Fig. 3D: We performed an additional experiment that further confirmed that there is no effect of CAIR1 on catalase protein levels; lines 246-249 read "In contrast to nca1 [41], the reduced catalase activities in the cair1 mutant alleles were not associated with altered catalase protein levels (Fig. 3B, and Supplementary Fig. 3C, D).".

6. In Supplemental Figure S9A, under the control condition, the cat2, cair1cat2 mutants appear to differ from the wild type, but no significant difference is observed in the corresponding quantitative

analysis. This is rather confusing. Besides, the differences in ROS levels shown in Figure S9C are difficult to distinguish. Please use other methods to quantify the ROS levels.

- Response :
The difference in the color scale in Supplementary Fig. 10A (i.e. previous Supplemental Figure S9A) between Control (T0) and Photoresp. (T8) was unfortunately not clear in the previous version and thus potentially misleading. We have now improved the labelling. Also, we clarified now that the quantification was done on individual plants grown separately and not as dense as in panel A, where even control plants may show enhanced photorespiration. Lines 1494-1495 now read: "Quantitative Fv/Fm measurements of individual plants grown as described in panel (A).". Although the difference is robust but very weak under control conditions, it is indeed additionally enhanced in the images due to the scaling.

We have removed the previous Supplemental Figure S9C panel from the new Supplementary Fig. 10 (lines 1497-1500), as well as its description and method section (lines 284-287 & 802-805). Regarding the reviewer's suggestion to use additional methods to quantify ROS levels, we note that accurate quantification of ROS in planta remains technically challenging. Presently we do not have any additional accurate and reliable method to quantify ROS levels established, except the RT-qPCR of broadly used ROS-marker genes (see Fig. 4C and 4D). It is of note that direct ROS measurements are generally associated with methodological limitations and are highly variable and quantification of extracted ROS is far from trivial. These issues have been extensively discussed in the literature: see, e.g., Queval et al (2008) Why are literature data for H_2O_2 contents so variable? A discussion of potential difficulties in the quantitative assay of leaf extracts. J Exp Bot (doi.org/10.1093/jxb/erm193); Veljovic-Jovanovic et al (2002) Are leaf hydrogen peroxide concentrations commonly overestimated? The potential influence of artefactual interference by tissue phenolics and ascorbate. Plant Physiol Biochem. (doi.org/10.1016/S0981-9428(02)01417-1); Noctor et al (2016) Oxidative stress and antioxidative systems: recipes for successful data collection and interpretation. Plant Cell Environ (doi.org/10.1111/pce.12726). In this regard it is of note that the book entitled "Reactive Oxygen Species in Plants. Method and Protocols" (Springer Protocols, Methods in Molecular Biology, 2022; https://link.springer.com/book/10.1007/978-1-0716-2469-2) does not contain any instructions regarding the direct quantification of ROS in plant extracts. This latter resource emphasizes the complexity of ROS detection and does not point to one direct and absolute quantification protocol. E.g. even if genetically encoded ROS sensors exist and might reflect stress levels in vivo they are simply a proxy and still depend on intracellular reductants. They are not straightforward to work with in photorespiratory stress conditions and several months would be needed to generate the relevant reporter lines.

7. In Figure 4F, cair1 showed reduced primary root growth compared to that in wild type at pH 8.5, but in Figure 4H, the root length of cair1mutant appears to be longer than that of the wild type. It is recommended that the root length need to be statistically analyzed under pH 9.0 conditions.

- Response :
The 5-d-old seedlings were transferred from pH 5.7 medium, on which cair1 shows longer primary roots (see Fig. 4E, including statistical analysis), to pH 9.0 medium, on which root growth is blocked. Thus, the slightly longer roots of cair1 on pH 9.0 are due to slightly longer roots to start with upon transfer from pH 5.7. As experiments at pH 9.0 were mainly for Fv/Fm measurements and general sensitivity to pH 9.0, plants were grown denser in Fig. 4G and H than optimal for root growth quantification. We thus prefer not to add root length measurements, as the experiment was not setup for that purpose (plants too dense and seen difference due to block of root growth after pH 5.7).

8.Figure 5C lacks a mock-treated time course as a necessary control to track CAIR1's basal oligomeric state and rule out non-specific effects. The same issue applies to Figure 5B.

- Response :
New parts of Fig. 5B and C (right panels): We have added mock-treated time course data for both experiments, as requested. CAIR1-YFP and CAIR1 protein stabilities were not changed by the mock treatments.

9.Figure 6H shows enhanced CAIR1-CAT2 interaction under oxidative stress, but it doesn't directly check CAIR1 oligomerization in the same samples. It is recommended to assess the oligomeric state of CAIR1 in the same samples.

- Response :
New Fig. 6I: We assessed the CAIR1 oligomeric state in the samples used for Fig. 6H. CAIR1 oligomerization was clearly increased after 48h under conditions of enhanced photorespiration.

Minor:
1. L165, the lack of CAIR1-NCA1 interaction in yeast two-hybrid assays contradicts the positive interactions observed in LCI and Co-IP assays. Please explain this discrepancy. Is the interaction indirect (e.g., bridged by CAT2)?

- Response :
Although our data are clear regarding the existence of a CAIR1-CAT2-NCA1 complex in planta and direct interaction between CAIR1 and CAT2 as well as CAT2 and NCA1 (the later confirming previously published data), we do not have strong evidence for a direct interaction between CAIR1 and NCA1, thus at present we indeed cannot exclude an indirect interaction bridged by CAT2. Even in the co-IPs in HEK293 cells we cannot exclude that human catalase bridges the interaction between Arabidopsis CAIR1 and NCA1 (although it has to be noted that our data suggests CAIR1 to have evolved in ferns – see Supplementary Fig. 2C – and NCA1 is also a plant specific protein).

2. Since anti-catalase antibody detect all three catalases (CAT1, CAT2, and CAT3), why was no catalase band detected in the cat2-2 mutant in Figure S5?

- Response :
New Supplementary Fig. 5A: Levels of CAT1 and CAT3 in cat2 mutants are variable but indeed are usually detectable with the general anti-catalase antibody used, even if rather weakly. We have thus replaced the previous western blot with data from an independent repetition to avoid confusion.

3. L181, the authors include GFP-PTS1 (a peroxisomal marker line) as a negative control in Figure 1C. It is better to explain the role of GFP-PTS1 when describing Figure 1C in the Results section.

- Response :
Lines 172-174 now read: "In contrast, CAIR1 was not co-immunoprecipitated with YFP alone and a peroxisomally localized GFP (GFP-PTS1), both providing negative controls (Fig. 1C, D, and Supplementary Table 1)."

3.Many western blot images appear overexposed of the protein band, possibly due to high protein concentrations, as in Figure 1D, Figure 6C, 6G, 6H and other panels in Supplemental Figures. Please optimize the images.

- Response :
Figure 6J: We have replaced the anti-GFP IP blot with a less exposed one.
To maintain consistency, the IP controls (e.g. anti-GFP western of GFP-trap purification) were obtained after reprobing the same membranes after stripping off co-IP bands, rather than on separate blots with diluted samples for IP testing. Due to efficient IP, the enrichments of the bait proteins appear overexposed. In addition, for most co-IP experiments, we used 35S-promoter-driven

YFP-HA as negative control. As a result, the YFP band for this negative control often appears overexposed in the IP western blots. Although we are convinced it would not alter the interpretation of our results, unfortunately, we do not have less exposed versions of other co-IPs than the one of Fig. 6J and cannot optimize the available images in an acceptable manner.

4. In Figures 5B, D, E, F and 5G, the actin band intensities are inconsistent. It is suggested to perform consistent quantification and conduct statistical analysis.

- Response :
Figs 5B, D, E, F and G: We have now added the quantifications of the band intensities normalized to the actin loading control band of the shown representative blots.

Reviewer #3 (Remarks to the Author)

The paper by Hu et al. identifies CAIR1 (AT5G11580), a previously uncharacterized RCC1-like protein in Arabidopsis, as a cytosolic regulator of catalase activity. The authors have unequivocally demonstrated using a diverse breadth of orthogonal assays that CAIR1 interacts with all three catalase isoforms and the chaperone NCA1 in the cytosol. Moreover, CAIR1 undergoes reversible, redox-dependent oligomerization via Cys-356 and Cys-545 that enhances its interaction with catalases under oxidative stress. The paper connects CAIR1 function to photorespiration stress, alkaline pH sensitivity, sucrose-dependent root elongation, and UV-B responses.

The mechanistic model for how CAIR1 controls peroxisomal import remains speculative. The central claim is that CAIR1 regulates catalase activity by facilitating peroxisomal import, yet there is no direct measurement of import rates. The evidence that GFP-CAT2 forms aggregates in cair1 is consistent with this model, but aggregation could also reflect misfolding, loss of cytosolic chaperone function, impaired tetramerization and CAT oxidation— none of which are distinguished. As it stands, the import model is plausible but not demonstrated. Along these lines, further exploration of whether CAIR1 affects the redox state of catalases could be informative.

- Response :
NCA1, a catalase chaperone, is essential for catalase protein stability and activity. However, our new data (Supplementary Fig. 6) show that the cair1 mutant does not affect the interaction between NCA1 and catalase, suggesting that CAIR1 is unlikely to affect cytosolic chaperone function and catalase folding. Together with our finding that CAIR1 regulates CAT2 localization to the cytosol under high redox conditions, these results support a model in which CAIR1 interacts with folded catalase in the cytosol to balance CAT2 distribution between the cytosol and peroxisomes under different physiological condition. Regarding the redox state of catalase, we did not obtain clear evidence that catalase activity is directly modified by oxidation. As shown in Supplementary Fig. 11B and C, no obvious band shift of catalase was observed under non-reducing conditions (without DTT). These results indicate that, unlike CAIR1, the oligomeric state of catalase is likely not substantially altered by ROS under our experimental conditions.

In Figure 6K, after 48 hours of enhanced photorespiration, catalase activity does not show statistically significant differences between WT and cair1. This result may nuance the interpretation of CAIR1's role in photorespiratory catalase regulation. Additionally, the effect of DTT on CAIR1-dependent catalase oligomerization is not examined and could provide useful insight into the redox regulation mechanism.

- Response :
The strongest difference in catalase activity between WT and cair1 is indeed observed under control conditions, where CAIR1-dependent regulation contributes to maintaining catalase activity. Under 48h prolonged photorespiratory stress, however, catalase activity is influenced by other mechanisms

that apparently do not require a functional CAIR1. Our data indicate that enhanced photorespiration simultaneously promotes CAIR1 oligomerization and interaction with catalases while also altering CAIR1 function. In addition, prolonged photorespiratory stress triggers extensive metabolic, redox, and transcriptional reprogramming that is likely to affect catalase activity. Consequently, the contribution of CAIR1 becomes less apparent after 48 h of treatment. Even though CAIR1 may still participate in the regulatory mechanisms, this is not reflected in the total catalase activity.

We have no evidence for CAIR1-dependent catalase oligomerization, and we do not claim such an effect in our work. We thus also have no assay to examine the effect of DTT on such a hypothetical effect.

The UVR8–CAIR1 connection remains poorly resolved. CAIR1 was identified as a UVR8 co-purifying protein, which motivates the study, yet the authors show that CAIR1 does not participate in canonical UVR8 signaling (Supplemental Figure S4). The UV-B section (lines 367–381) demonstrates reduced catalase activity under UV-B and weakened CAIR1–CAT2 interaction, but the mechanistic connection is vague. The authors themselves acknowledge they cannot distinguish whether UVR8 indirectly suppresses the CAIR1–CAT2 interaction (via altered ROS levels) or directly disrupts it. This section feels underdeveloped relative to its prominence in the narrative.

- Response :
We agree with the reviewer that at this stage the UVR8-CAIR1 connection is mechanistically the least resolved issue, and obviously a focus of our future research. It is true that the UVR8-CAIR1 interaction was our main entry point into this research, but it was indeed largely taken over by our growing interest on catalase localization and regulation developing through the discovery and novelty of CAIR1 function in this work – which is also clearly reflected in the manuscripts title and focus. The detailed underlying mechanism of how UVR8 and CAIR1 mediate UV-B repression of catalase activity remains to be investigated in future studies.

The AP–MS data show that CAIR1 co-purifies with both UVR8 and catalases, yet UVR8 and CAT never appear in each other's purifications (lines 196–200). If CAIR1 bridges these two complexes, the absence of a ternary complex is important to reconcile. The authors mention this observation but do not discuss its implications for the proposed model.

- Response :
CAIR1 was identified in both the CAT2 and UVR8 complexes. However, UVR8 and CAT2 were never detected together in the same purification, suggesting that the CAIR1–UVR8 and CAIR1–CAT2 complexes are independent. Notably, the CAIR1–CAT2 complex was predominant under our purification conditions, whereas the CAIR1–UVR8 interaction was relatively weak. Future studies will be required to determine how CAIR1 dynamically balances these interactions and whether specific conditions promote its association with different partners. Now lines 459-462 (numbering according to the marked up version) read: "CAIR1 was identified in both the CAT2 and UVR8 complexes. However, CAT2 did not co-purify with GFP-UVR8 nor did UVR8 with GFP-CAT2, suggesting that CAIR1 interacts with UVR8 and CAT2 in a mutually exclusive manner.".

The CAIR1 12C-A (all 12 cysteines mutated) and CAIR1 C356A, C545A mutants both abolish redox-dependent oligomerization, but the single-cysteine mutants (CAIR1 C356A and CAIR1 C545A) are only tested for oligomerization (Figure 7A) and not for physiological rescue or catalase interaction in planta. This leaves open whether one or both cysteines are individually required for function, or whether additional cysteines contribute.

- Response :
We appreciate the reviewer's suggestion to test the single cysteine mutations CAIR1-C356A and CAIR1-C545A in transgenic complementation and catalase interaction assays. Our findings

indicate that ROS mediates CAIR1 oligomerization, thereby altering the interaction between CAIR1 and catalase. We found that both these cysteine residues are exposed to the surface and are conserved in CAIR1 from different species (Supplementary Fig. 12D). Moreover, in Fig. 7A and Supplementary Fig. S12E, CAIR1-C356A or CAIR1-C545A do not alter CAIR1 oligomerization. Therefore, we just focused on double cysteines mutations instead of single mutations for the complementation assays.

Total cellular catalase activity is measured from crude extracts, which cannot distinguish between cytosolic and peroxisomal pools. Since the model proposes that CAIR1 enhances catalase activity by promoting peroxisomal import, it would be important to measure catalase activity in fractionated subcellular compartments to confirm that the activity reduction in cair1 specifically reflects loss of peroxisomal catalase. Complementary time-lapse confocal imaging of CAT-GFP import into peroxisomes—using lines expressing a fluorescent peroxisomal marker—could also provide direct evidence supporting the proposed import mechanism.

- Response :

We appreciate the reviewer's suggestion. To address this point, we explored the possibility of measuring catalase activity in subcellular fractions following the approach described by Al-Hajaya et al. (2022). However, we were unable to sufficiently enrich a peroxisomal protein (HPR1) and to exclude a cytosolic maker protein (UGPase) in the peroxisomal fraction based on western blot analysis after subcellular fraction. This was likely due to the fact that peroxisomes are highly fragile, as stated in Mhamdi et al. Arch Biochem Biophys (2012): "As peroxisomes are very fragile, they are easily ruptured during fractionation experiments so reports of catalase in other locations, particularly based on cell fractionation must be treated with scepticism". Unfortunately, to our knowledge, there is currently no widely adopted protocol that allows to efficiently and reliably measure catalase activity in different subcellular fractions from plant extracts. It should also be noted that although catalase activity is high in plant extracts it is not that stable over time and thus any successful fractionation procedure needs to be fast, not to lose catalase activity during purification. It is to be expected that reliable and reproducible catalase activity assays to quantify the differences between compartments in different genotypes will not be easy to achieve, even if reducing sample number (replicates) per experiment. At present we base our conclusion on robust and clear interaction data, effect of CAIR1 on catalase localization, and cair1 mutant phenotypes, and we consider establishing a reliable and robust fractionation protocol to compare different genotypes – bearing in mind the high fragility of peroxisomes and the instability of catalase activity over time – as out of scope for this work.

As GFP-CAT2 localizes to peroxisomes, we do not think that its import into peroxisomes can be studied reliably by time-lapse confocal imaging. A more suitable approach would be FRAP or related assays, which can directly measure protein trafficking dynamics. Establishing such assays requires additional transgenic lines and appropriate controls that are not currently available. We will attempt to reliably and quantitatively measure GFP-CAT2 peroxisomal import using FRAP assays in the future, when additional control lines have been generated.

Minor comments

CAIR1 and NCA1 co-purify and both interact with catalases, yet the Y2H assay shows no direct CAIR1–NCA1 interaction (line 165). Does CAIR1 require NCA1 for its function? Does NCA1 still function in cair1 mutants? The epistatic relationship is barely explored. The cair1 mutants partially phenocopy cat2 and nca1, but the mechanistic distinction between CAIR1 and NCA1 contributions to catalase regulation is left unclear.

- Response :

New Supplementary Fig. 6: we performed a new experiment testing whether CAIR1 affects the NCA1-CAT2 interaction but found no clear evidence for such a mechanism (see also related response to Reviewer #2).

Regarding whether NCA1 still functions in cair1 mutants it should be noted that in contrast to nca1, the cair1 mutant does not show reduced catalase levels, suggesting that NCA1 chaperone activity is not affected. Whether CAIR1 requires NCA1 for its function is less clear at present. Unlike NCA1, as a catalase chaperone required for catalase protein stability, cair1 mutant or OX-CAIR1 did not change catalase protein levels. Combined with our new data (Supplementary Fig. 6) that loss-of CAIR1 does not change interaction of NCA1 with catalases, we propose that CAIR1 probably directly regulates catalase function by affecting its subcellular distribution.

Reviewer #4 (Remarks to the Author)

I co-reviewed this manuscript with one of the reviewers who provided the listed reports. This is part of the Nature Communications initiative to facilitate training in peer review and to provide appropriate recognition for Early Career Researchers who co-review manuscripts.

- Response :
Thank you very much for co-reviewing our manuscript; highly appreciated.

<u>*Additional changes during revision:*</u>

- *We introduced several minor changes in accordance with the Nature Communications 'Author Guidelines'.*

Point-by-point response to reviewers' comments:

Reviewer #1 (Remarks to the Author):

The authors have satisfactorily addressed my concerns in this revised manuscript

- *Response :*
We thank the reviewer for the positive evaluation of our revised manuscript.

Reviewer #2 (Remarks to the Author):

The authors have addressed most of the concerns raised in the previous round of review and the manuscript has been improved accordingly. However, a few issues remain that require further clarification before the manuscript can be accepted for publication.

- *Response :*
We thank the reviewer for the positive assessment and appreciate the additional constructive comments, which we address below.

1. In the new added Supplementary Figure S6, the Input blots probed with the CAIR1 antibody show numerous non-specific bands, making it difficult to identify the specific CAIR1 signal. Moreover, the co-immunoprecipitated catalase levels appear markedly different between the two groups, which should be explained. Whether CAIR1 affects the NCA1-catalase interaction needs further discussion.

- *Response :*
We agree that our anti-CAIR1 antibody, while reliably detecting CAIR1, also recognizes several non-specific bands, including one migrating close to the CAIR1 signal in the input samples. However, following co-immunoprecipitation with NCA1-GFP, the anti-CAIR1 immunoblot shows a clear and specific CAIR1 signal, supporting the specificity of the interaction. Importantly, the interaction between NCA1 and CAIR1 is also supported by several independent experiments presented in the manuscript (see co-IP in Fig. 1D, the AP-MS data analysis, and co-IP following heterologous expression in HEK cells, Fig. S7C). Because the primary purpose of Supplementary Fig. 6 is to assess the interaction between NCA1 and catalases rather than to demonstrate the NCA1-CAIR1 interaction, the CAIR1 immunoblot is not essential for the main conclusion and could be removed if the reviewer or editor considers this preferable.

Regarding the apparent reduction in catalase co-immunoprecipitation in the cair1 background, we agree that the first experiment shows a modest decrease. However, this is accompanied by a similar reduction in the amount of immunoprecipitated NCA1-GFP bait, making it difficult to attribute the difference specifically to altered NCA1-catalase interaction. Furthermore, the independent biological replicate shown below does not reveal a clear reduction in catalase co-immunoprecipitation in the cair1 background. Taken together, these data do not provide evidence for a substantial effect of CAIR1 on the interaction between NCA1 and catalases, although we agree that a subtle effect cannot be excluded.

To better reflect this interpretation, we have moderated our wording in the revised manuscript. Lines 176–177 now read: "However, absence of CAIR1 did not substantially affect the co-IP of catalases with NCA1-GFP (Supplementary Fig. 6).". In addition, the heading of Supplementary Fig. 6 (also lines 1489–1490 in the marked-up copy) has been changed to: "CAIR1 has little effect on the interaction of NCA1 with catalases.".

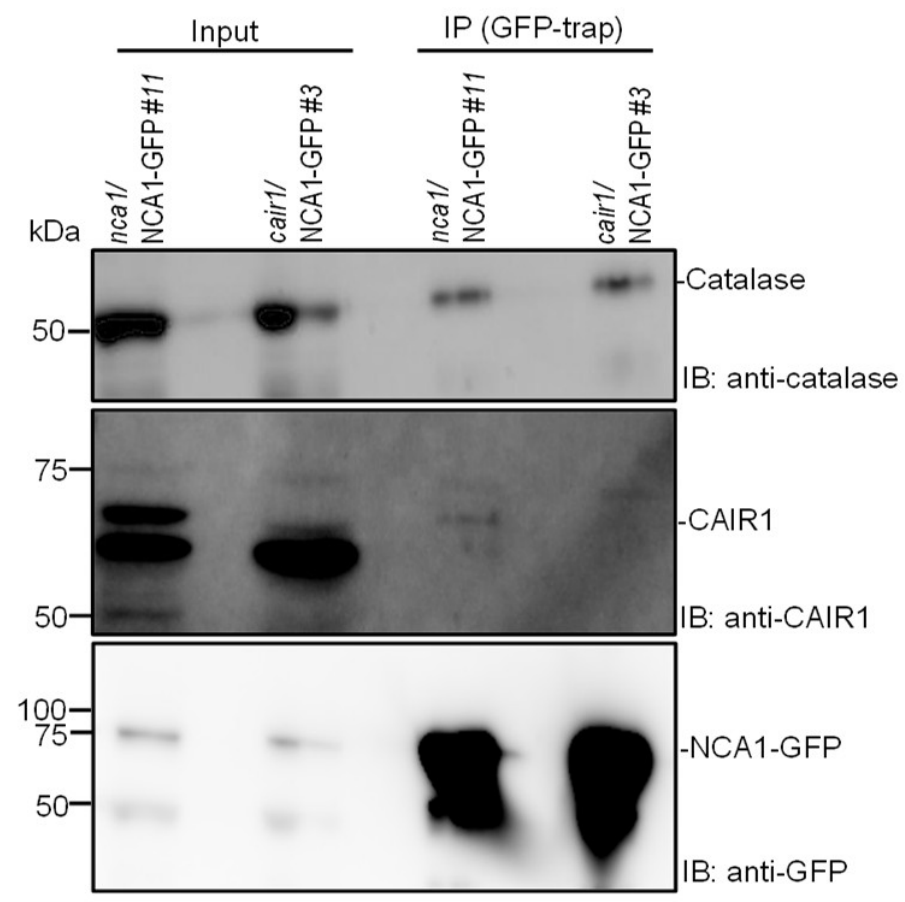

Independent repetition of the experiment shown in Supplementary Fig. 6.

2. In Figures 5B, D and G, the signal intensities of Actin loading control bands are inconsistent. To ensure the reliability of quantitative results, we suggest the authors repeat the Western blot experiments with equal total protein loading across all samples, and then normalize the signal intensities of target protein bands against Actin for quantitative analysis.

- Response :
The Western blot experiments were repeated, as suggested, and the blots in Fig. 5B, D, and G, have been exchanged.

Reviewer #3 (Remarks to the Author):

The manuscript can be accepted for publication

- Response :
We thank the reviewer for the positive evaluation of our revised manuscript.
